# Supplementary material for: Mechanism of beta-arrestin 1 mediated Src activation via Src SH3 domain revealed by cryo-electron microscopy
Source: Nat Commun. 2026 Feb 20;17:2973. doi: 10.1038/s41467-026-69884-1 (PMC13035853; doi:10.1038/s41467-026-69884-1)
Supplement: Supplementary file 1 — Supplementary Information [file 41467_2026_69884_MOESM1_ESM.pdf]

## Supplementary Information

# Mechanism of beta-arrestin 1 mediated Src activation via Src SH3 domain revealed by cryo-electron microscopy

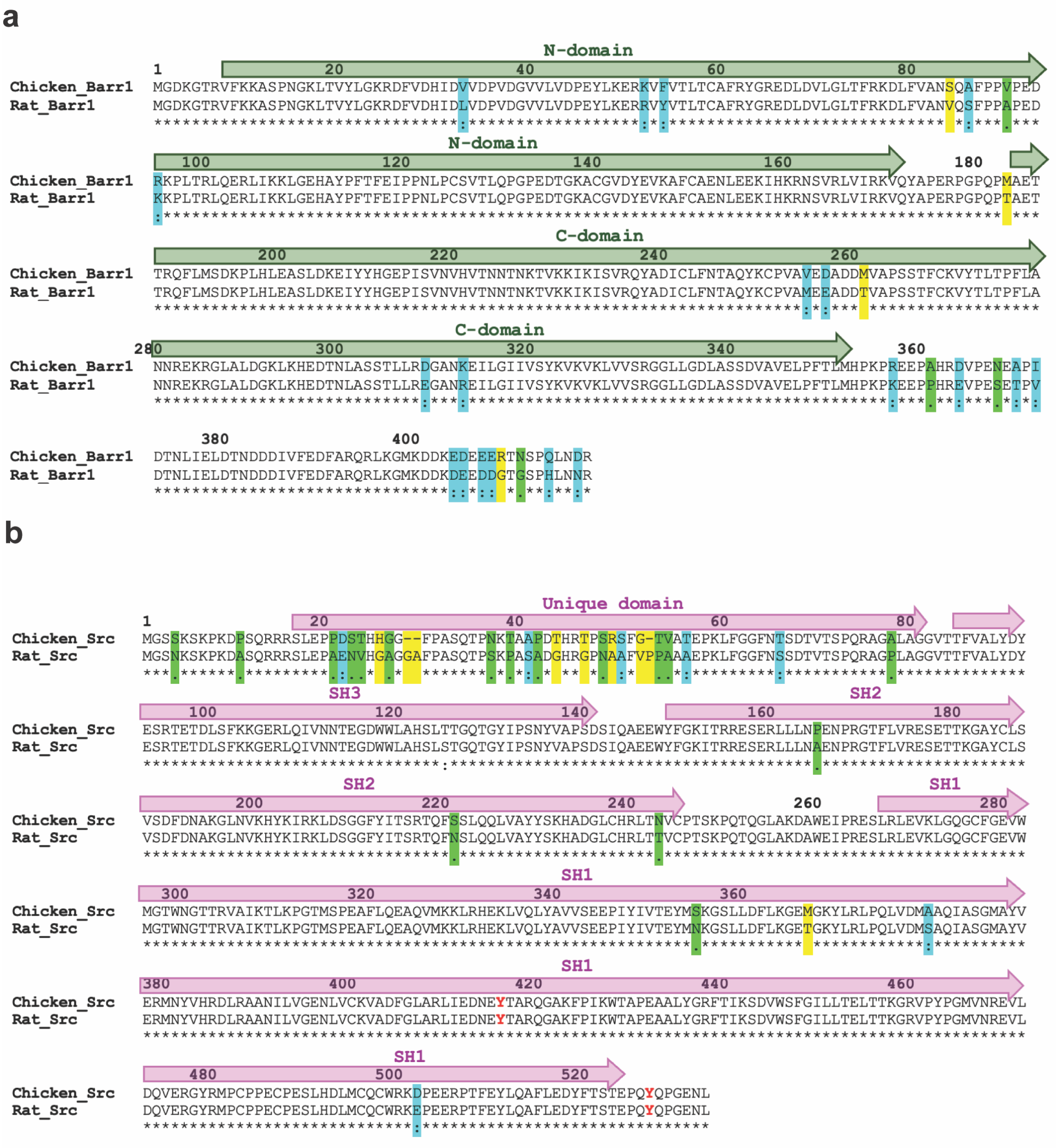

**Supplementary Fig. 1. | Alignment of rat (*Rattus norvegicus*) and chicken (*Gallus gallus*) sequences of β-arrestin 1 (βarr1) (a) and Src (b).** Fully conserved residues are marked with asterisk (*); strongly conserved residues are marked with colon (:) and shaded in cyan; weakly conserved residues are marked with period (.) and shaded in green; non-conserved residues are shaded in yellow. Src active loop tyrosine (Y416) and C-tail tyrosine (Y527) are colored in red. Domains are indicated with arrows and labeled. Sequence alignment was performed in Clustal Omega (https://www.ebi.ac.uk/jdispatcher/msa/clustalo).

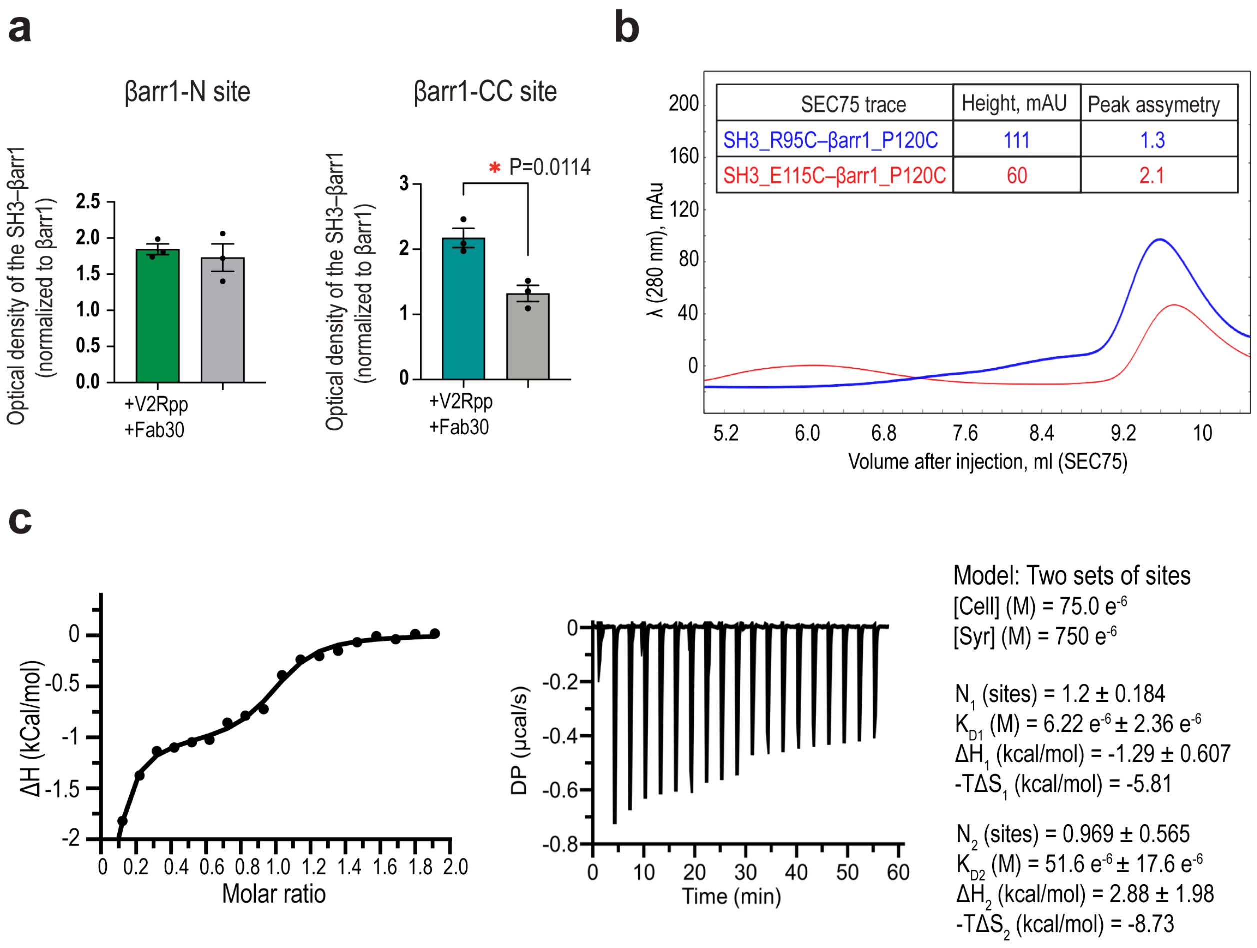


**Supplementary Fig. 2. | βarr1 uses two distinct sites to bind SH3**. **a,** Densitometry analysis of SH3–βarr1 disulfide trapping with and without synthetic phosphopeptide mimicking the C-tail of vasopressin 2 receptor (V2Rpp) and stabilizing antibody Fab30 (mean ± standard error of mean (SEM), n=3 independent experiments). Statistical analysis was performed using two-tailed Student's t-test. **b,** Analytical size-exclusion chromatography profile of SH3_R95C–βarr1_P120C and SH3_E115C–βarr1_P120C disulfide trapped complexes (mAU, milli-absorbance units). Data are representative of n=3 independent experiments. **c,** Isothermal titration calorimetry of SH3 binding to βarr1–V2Rpp. Left panel: integrated heat (after deducting heat of dilution) per injection of SH3 into βarr1–V2Rpp solution in the cell based on the molar ratio of each injection. Middle panel: raw titration data showing the heat rate associated with each dilution per injection versus time into βarr1–V2Rpp solution in the cell. Right panel: thermodynamic parameters of SH3–βarr1–V2Rpp. Data are representative of n=3 independent experiments.

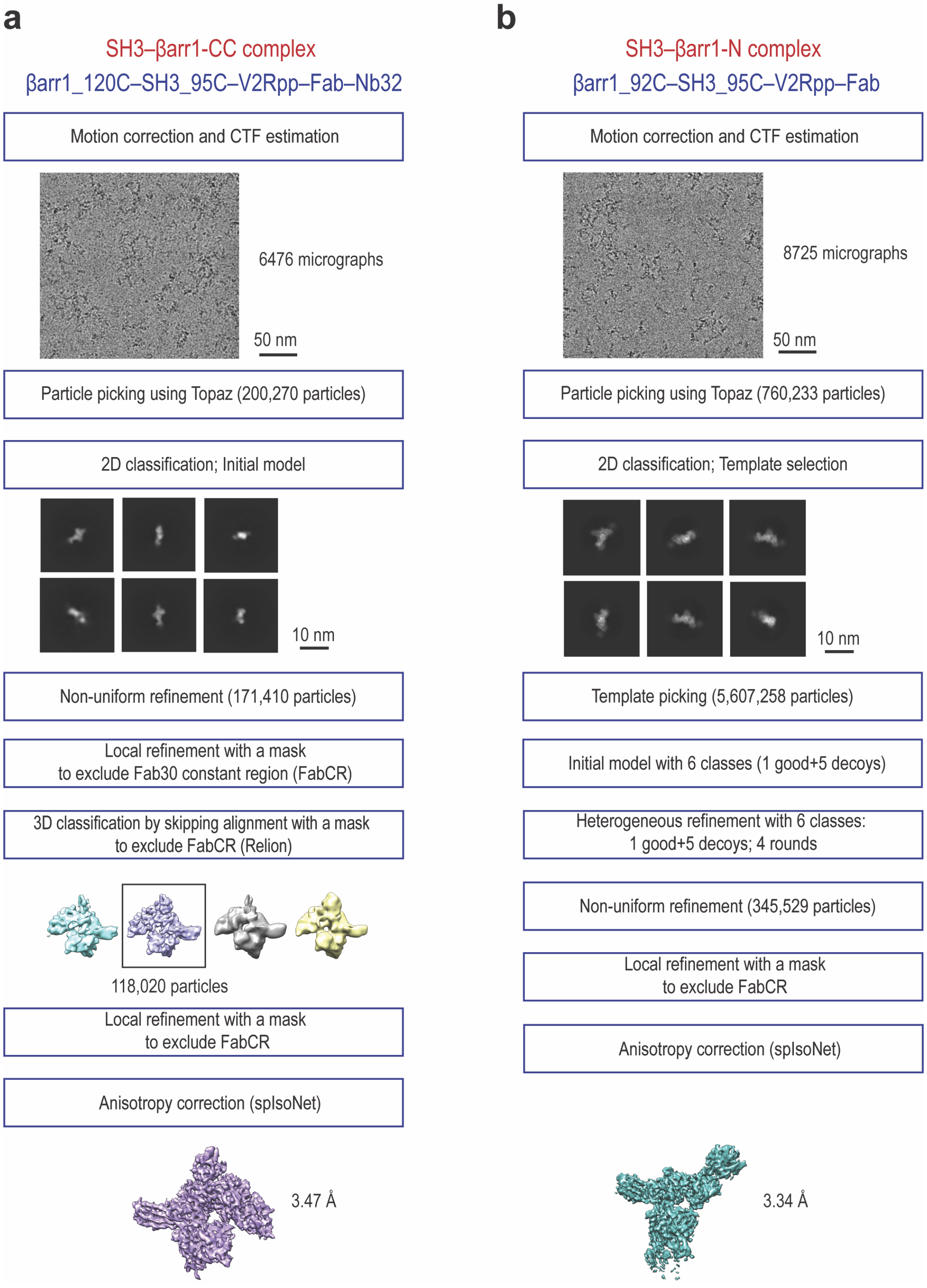


**Supplementary Fig. 3. | Flow chart of cryo-EM data processing of SH3–βarr1-CC (a) and SH3–βarr1-N (b) complexes.**

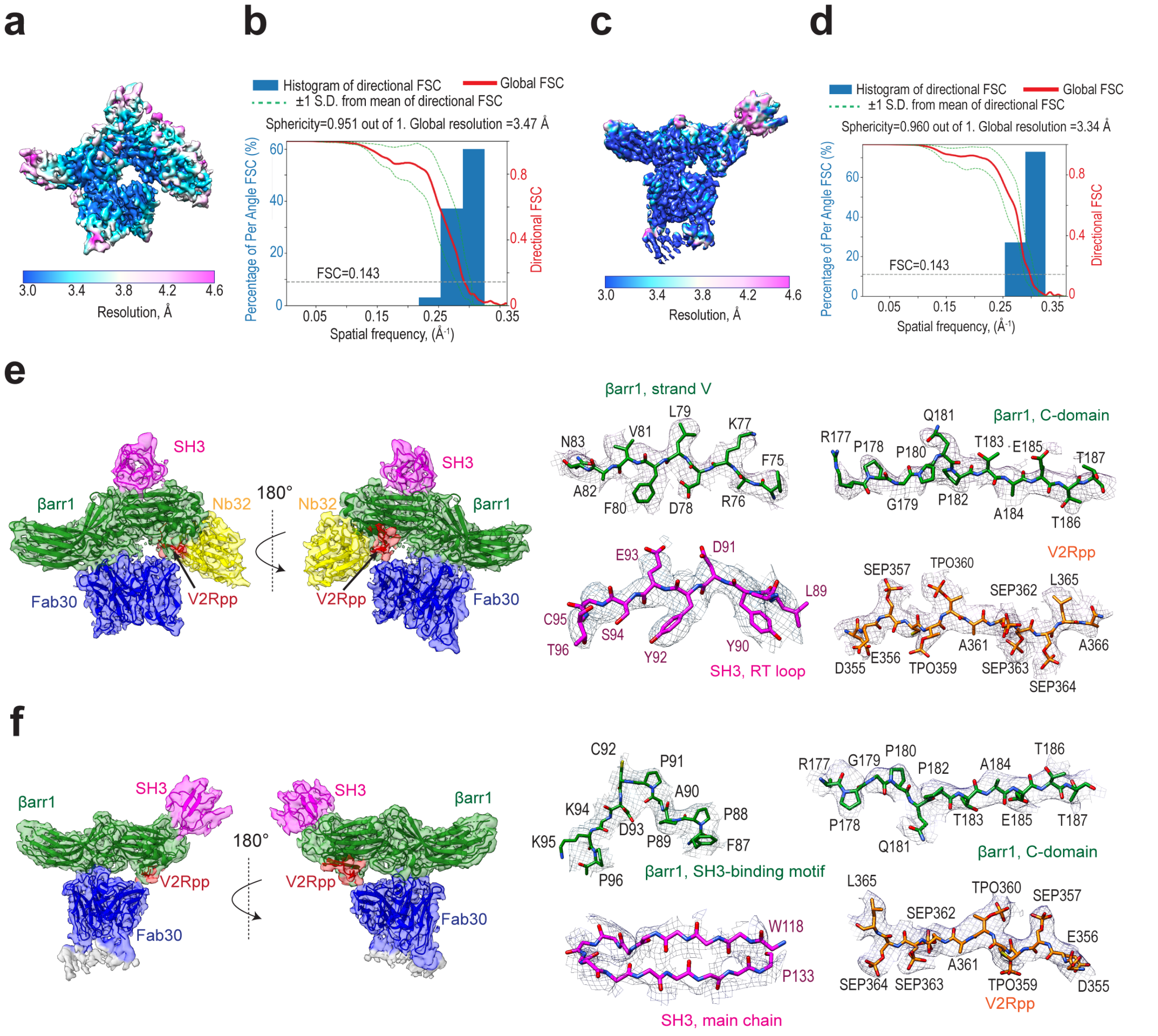


**Supplementary Fig. 4. | Cryo-electron microscopy of SH3–βarr1 complexes. a**, Cryo-EM map of SH3–βarr1-CC coloured by local resolution; resolution is reported at the Fourier Shell Correlation (FSC) threshold of 0.143. Map contour level is 0.83. **b**, Histogram and directional FSC plot measuring directional resolution anisotropy for SH3–βarr1-CC. Sphericity values were determined at an FSC threshold of 0.5 with the 3DFSC software.1 **c**, Cryo-EM map of SH3–βarr1-N coloured by local resolution; resolution is reported at the Fourier Shell Correlation (FSC) threshold of 0.143. Map contour level is 0.50. **d**, Histogram and directional FSC plot measuring directional resolution anisotropy for SH3–βarr1-N. **e**, Left panel: Two viewing angles of model fit to map and cryo-EM density at different parts of the SH3–βarr1-CC (green, βarr1; magenta, SH3; red, V2Rpp; blue, Fab30; yellow, Nb32). Map contour level is 0.83. Right panel: cryo-EM density at different parts of the SH3–βarr1-CC complex (green, βarr1; magenta, SH3; orange, V2Rpp). The upsample map is used (0.69 Å/pix); map contour level is 0.3. **f**, Left panel: Two viewing angles of model fit to map and cryo-EM density at different parts of the SH3–βarr1-N (green, βarr1; magenta, SH3; red, V2Rpp; blue, Fab30). Map contour level is 0.50. Right panel: cryo-EM density at different parts of the SH3–βarr1-N complex (green, βarr1; magenta, SH3; orange, V2Rpp). The upsample map is used (0.69 Å/pix); map contour level is 0.2.

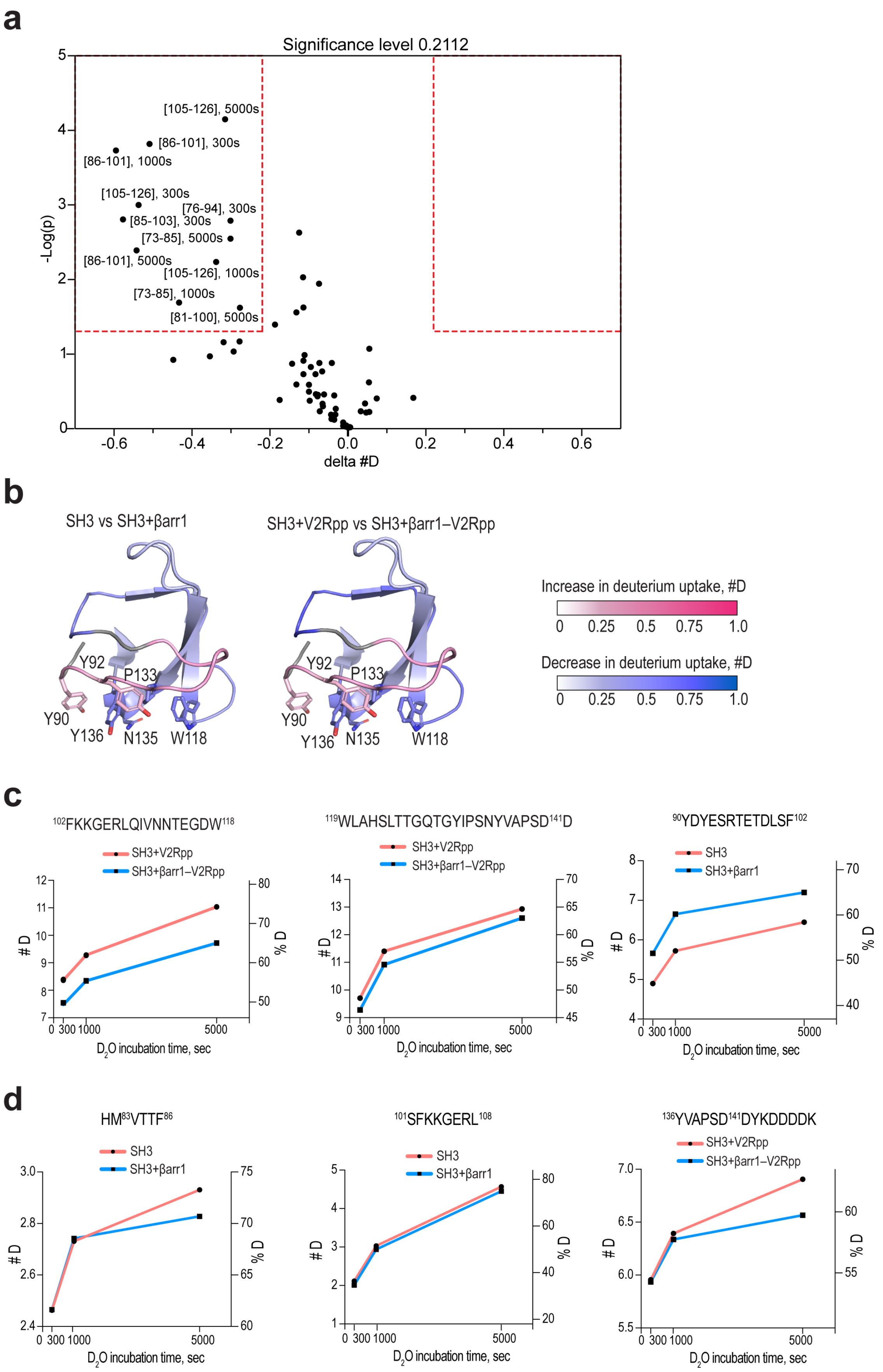
a
Significance level 0.2112
[105-126], 5000s
[86-101], 300s
[86-101], 1000s
[105-126], 300s
[76-94], 300s
[85-103], 300s
[73-85], 5000s
[86-101], 5000s
[105-126], 1000s
[73-85], 1000s
[81-100], 5000s
-Log(p)
delta #D
b
SH3 vs SH3+βarr1
SH3+V2Rpp vs SH3+βarr1–V2Rpp
Y92
P133
Y90
Y136
N135
W118
Increase in deuterium uptake, #D
0 0.25 0.5 0.75 1.0
Decrease in deuterium uptake, #D
0 0.25 0.5 0.75 1.0
c
102FKKGERLQIVNNTEGDW118
SH3+V2Rpp
SH3+βarr1–V2Rpp
119WLAHSLTTGQTGYIPSNYVAPSD141D
SH3+V2Rpp
SH3+βarr1–V2Rpp
90YDYESRTETDLSF102
SH3
SH3+βarr1
# D
% D
D2O incubation time, sec
d
HM83VTTF86
SH3
SH3+βarr1
101SFKKGERL108
SH3
SH3+βarr1
136YVAPSD141DYKDDDDK
SH3+V2Rpp
SH3+βarr1–V2Rpp
# D
% D
D2O incubation time, sec

**Supplementary Fig. 5. | HDX profile changes in βarr1 and SH3 following co-incubation. a,** Volcano plot of βarr1 peptides (residues 66–126) near the SH3 interface; peptides with significantly reduced deuterium uptake and corresponding time points are labelled. Results for free βarr1 are shown. **b,** Structure of SH3 (PDB: 2PTK) upon co-incubation with free βarr1 (left panel) or V2Rpp-activated βarr1 (right panel). Regions with decreased and increased deuterium uptake are shaded in dark-blue and magenta, respectively. Only regions that showed differences between the states in deuterium uptake (#D) > 0.2 Da in at least two overlapping peptides and at all time points are indicated. **c,** HDX profile of representative peptides in SH3 in the proximity to βarr1 interface (salmon, SH3 or SH3 with V2Rpp; blue, SH3 in the presence of βarr1 or βarr1–V2Rpp). # D and % D – deuterium uptake, Da and %, respectively. **d,** Examples of peptides showing no consistent changes in deuterium uptake upon co-incubation with βarr1 or βarr1–V2Rpp (salmon, SH3 or SH3 with V2Rpp; blue, SH3 in the presence of βarr1 or βarr1–V2Rpp). The data presented in **b**–**d** are based on single HDX experiment (n=1).

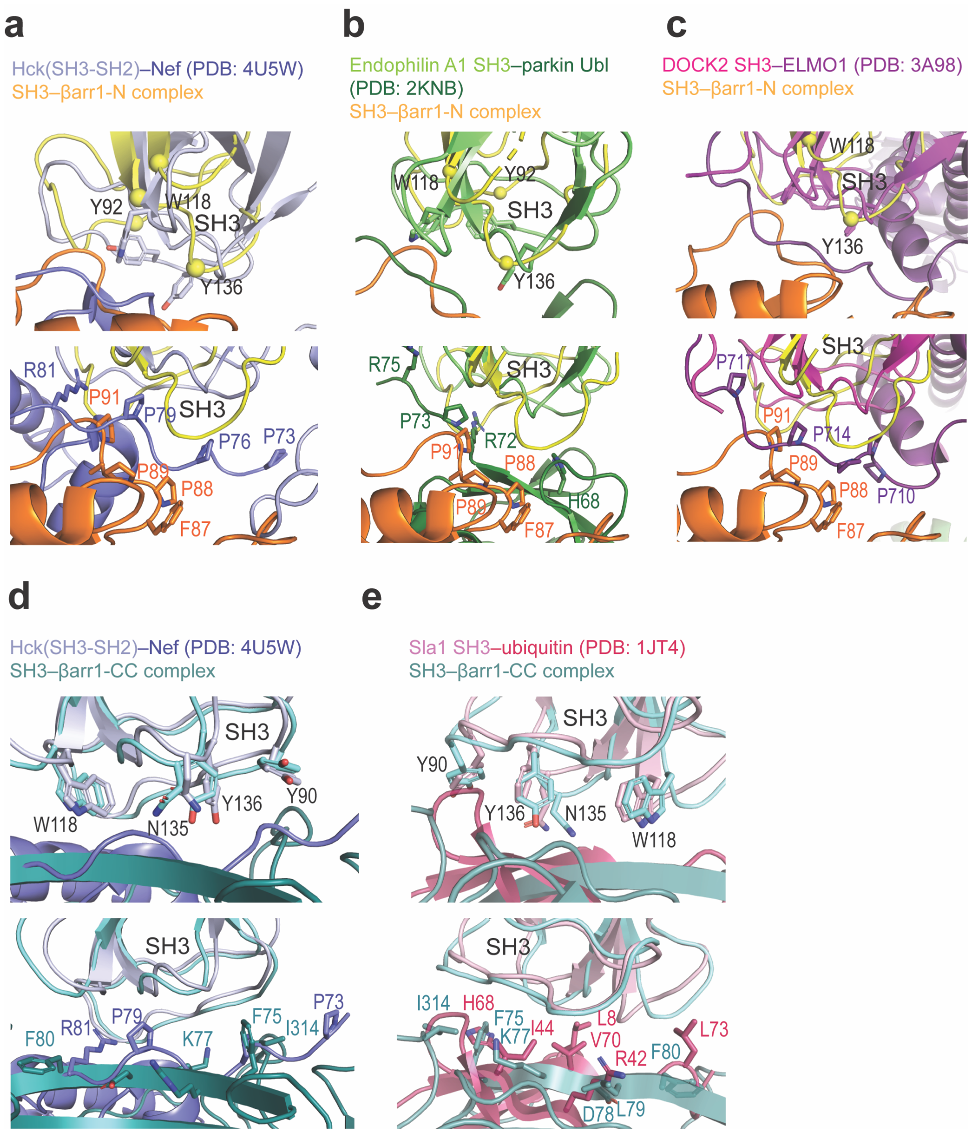


**Supplementary Fig. 6. | Structural superpositions of SH3 domains in SH3–βarr1-N (yellow), SH3–βarr1-CC (cyan) and other SH3-binding complexes. a,** SH3–βarr1-N (yellow) and Hck(SH3-SH2)-Nef (slate) (PDB: 4U5W). **b,** SH3–βarr1-N (yellow) and endophilin A1 SH3–parkin Ubl (green) (PDB: 2KNB). **c,** SH3–βarr1-N (yellow) and DOCK2 SH3–ELMO 1 (magenta) (PDB: 3A98). **d,** SH3–βarr1-CC (cyan) and Hck(SH3-SH2)-Nef (slate) (PDB: 4U5W). **e,** SH3–βarr1-CC (cyan) and Sla1 SH3-ubiquitin (pink) (PDB: 1JT4). The top panel shows the interacting residues in SH3 (Src SH3 numbering is used), the bottom panel shows interacting residues in βarr1 and the SH3-binding protein.

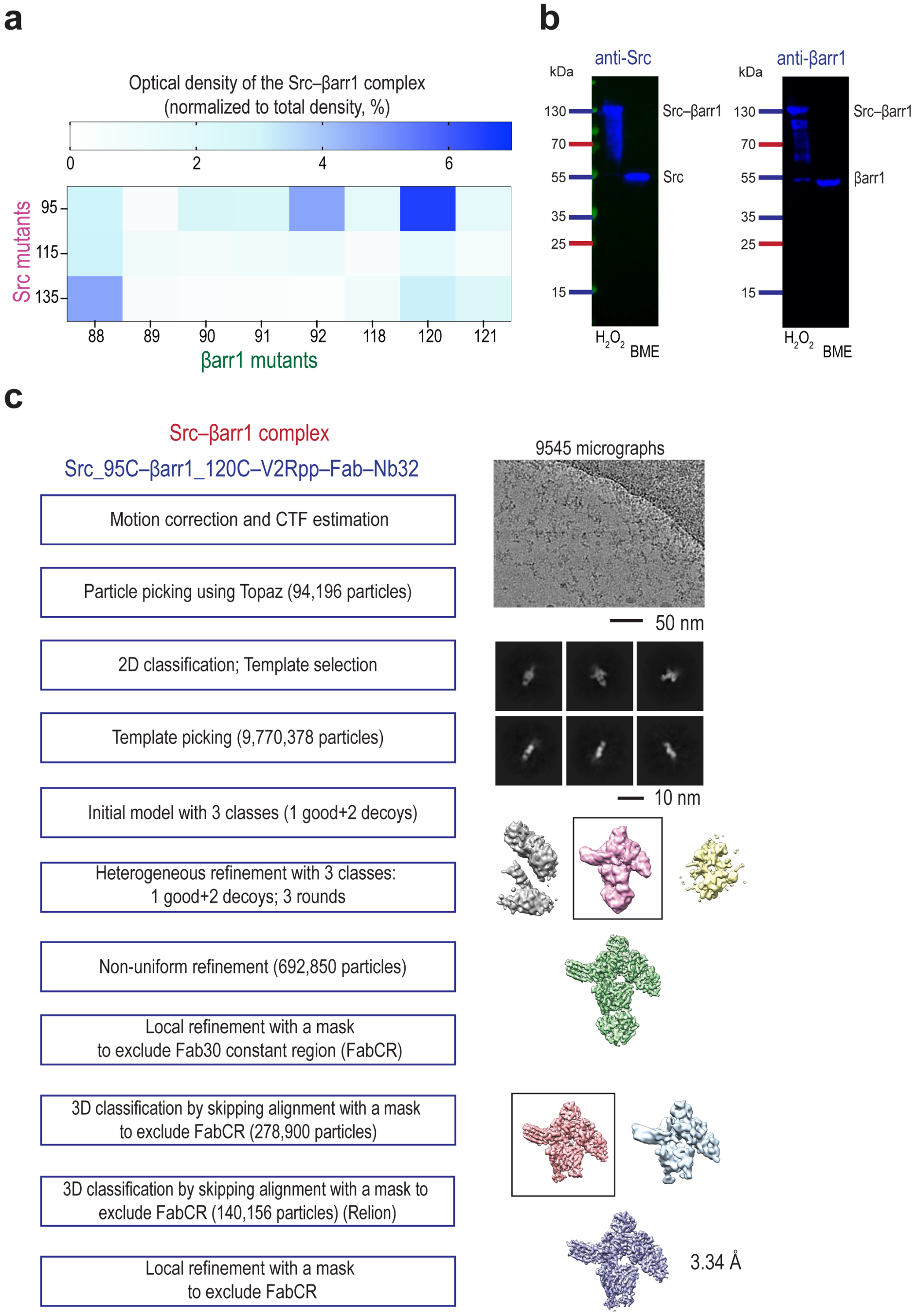


**Supplementary Fig. 7. | Complex formation and cryo-EM data processing of Src–βarr1-CC. a,** Complex formation between Src and βarr1 mutants revealed by disulfide trapping; densitometry analysis of Coomassie blue gels. The Src–βarr1-CC complex band was normalized to the total density of all bands in each sample. Prior to disulfide trapping reactions, βarr1 was activated by V2Rpp (Mean values; n=3, independent experiments). **b,** Western blot of disulfide trapping reaction with βarr1_120C and Src_R95C. 130-kDa band is detected by both anti-βarr1 (A1CT) and anti-Src antibodies, confirming that the band is the covalent Src–βarr1-CC complex (BME - β-mercaptoethanol). Data are representative of n=3 independent experiments. **c,** Flow chart of cryo-EM data processing of the Src–βarr1-CC complex.

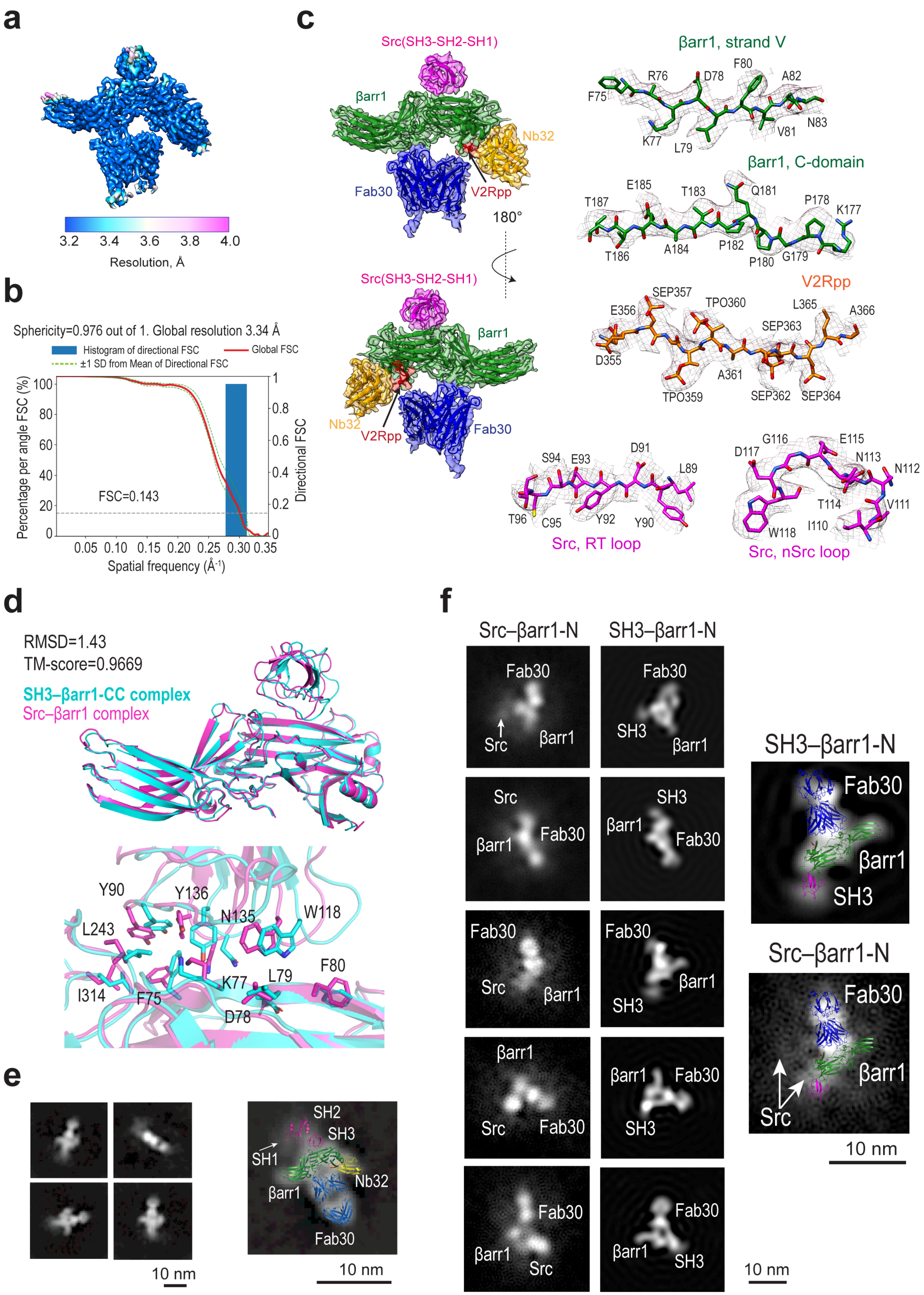
a
3.2
3.4
3.6
3.8
4.0
Resolution, Å
b
Sphericity=0.976 out of 1. Global resolution 3.34 Å
Histogram of directional FSC
Global FSC
±1 SD from Mean of Directional FSC
Percentage per angle FSC (%)
Directional FSC
FSC=0.143
Spatial frequency (Å-1)
c
Src(SH3-SH2-SH1)
βarr1
Nb32
Fab30
V2Rpp
180°
βarr1, strand V
βarr1, C-domain
V2Rpp
Src, RT loop
Src, nSrc loop
d
RMSD=1.43
TM-score=0.9669
SH3–βarr1-CC complex
Src–βarr1 complex
Y90
Y136
N135
W118
L243
I314
F75
K77
L79
F80
D78
e
SH2
SH3
SH1
βarr1
Nb32
Fab30
10 nm
f
Src–βarr1-N
SH3–βarr1-N
Fab30
Src
βarr1
SH3
10 nm

**Supplementary Fig. 8. | Cryo-electron microscopy of Src–βarr1 complexes. a,** Cryo-EM map of the Src–βarr1-CC complex coloured by local resolution; resolution is reported at the FSC threshold of 0.143. Map contour level is 0.55. **b,** Histogram and directional FSC plot. A sphericity of 0.976 determined at an FSC threshold of 0.5 indicates isotropic angular distribution. Directional FSC determination was performed with the 3DFSC software.[1] **c,** Left panel: Two viewing angles of model fit to map and cryo-EM density at different parts of the Src–βarr1-CC (green, βarr1; magenta, Src; red, V2Rpp; blue, Fab30; yellow, Nb32). Map contour level is 0.55. Right panel: cryo-EM density at different parts of the Src–βarr1-CC complex (green, βarr1; magenta, Src; orange, V2Rpp). The upsample map is used (0.72 Å/pix); map contour level is 0.1. **d,** Structural superposition of the Src–βarr1-CC (magenta) and SH3–βarr1-CC (cyan) complexes (Fab30, V2Rpp and Nb32 are not shown). Root mean square deviation (RMSD) and the template modeling score (TM-score) of the structures was calculated using the TM-score function[2]. **e,** Representative 2D classes of Src–βarr1-CC (left) and the 2D class with the fitted model (right). **f,** Matching of the 2D classes of the Src–βarr1-N complex with the projections of the SH3–βarr1-N map low-pass filtered to 25 Å using cluster selection mode in Reference Based Auto Select 2D module in CryoSPARC (left panel) and representative 2D classes of the complexes with the fitted model of SH3–βarr1-N (right panel); green, βarr1; magenta, SH3; blue, Fab30; red, V2Rpp).

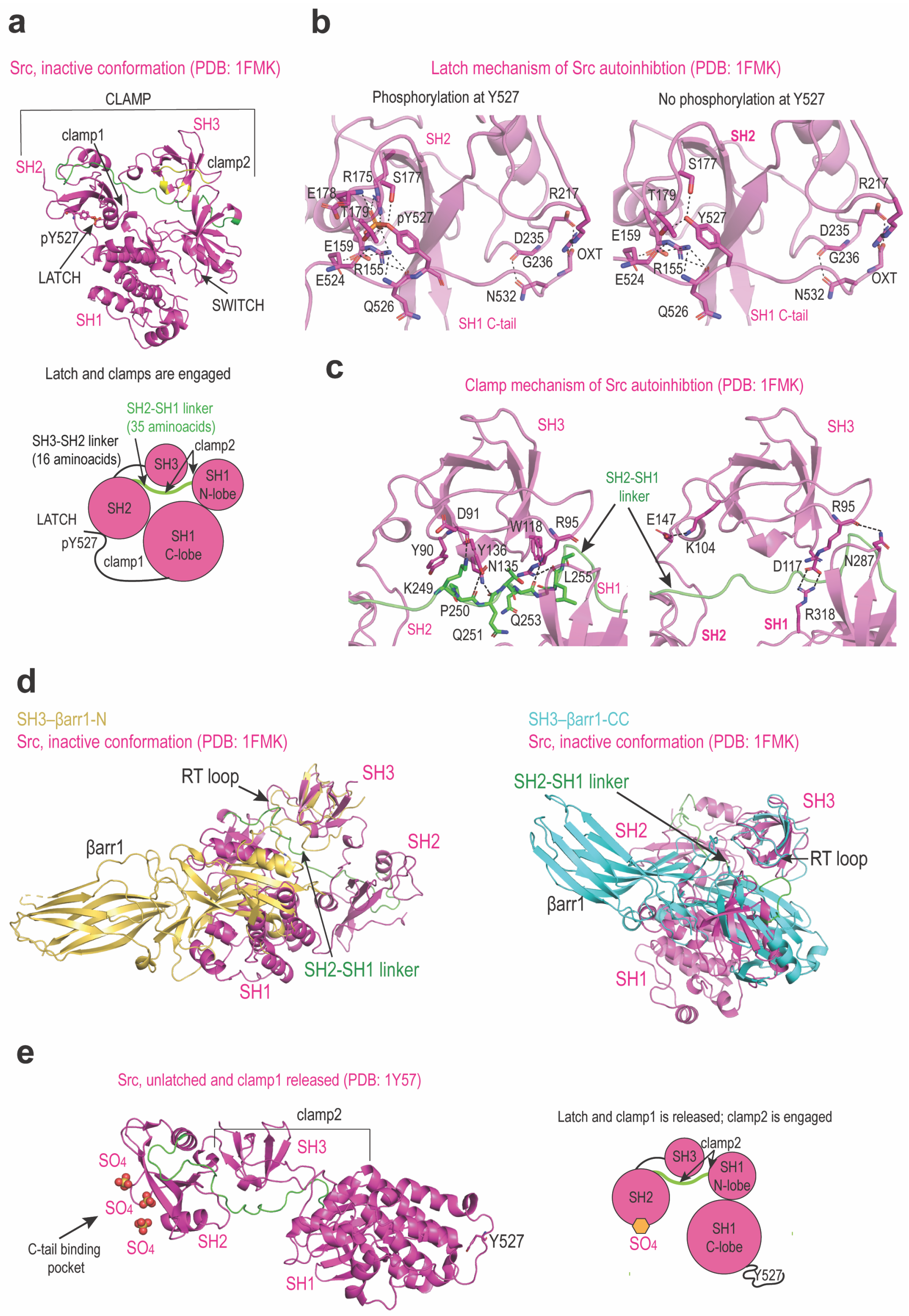


**Supplementary Fig. 9. | Autoinhibition of Src. a,** Structure of autoinhibited Src (PDB: 1FMK), magenta. Parts of SH3 interacting with βarr1 are colored in yellow. SH2-SH1 linker is colored in green; phosphorylated Y527 is labeled. **b-c,** Latch (**b**) and clamp (**c**) mechanisms of Src autoinhibition (PDB: 1FMK), cartoon representation (magenta). The hydrogen bonds are depicted as dashed lines; interacting residues are labeled. SH2-SH1 linker is colored in green. **d,** Structural superpositions of SH3–βarr1-N (yellow) and SH3–βarr1-CC (cyan) with inactive Src (PDB: 1FMK, magenta). **e,** Structure of unphosphorylated Src (PDB: 1Y57), magenta. SH2-SH1 linker is colored in green; phosphorylated Y527 is labeled. Note that the C-tail binding pocket on SH2 is occupied by sulfate ions originating from crystallization solution.

## Supplementary Table 1. Cryo-EM data collection, refinement and validation statistics

| | **SH3_95C–βarr1_120C–V2Rpp–Fab30–Nb32 (SH3–βarr1-CC)** (EMD-45977) (PDB 9CX3) | **SH3_95C–βarr1_92C–V2Rpp–Fab30 (SH3–βarr1-N)** (EMD-45982) (PDB 9CX9) | **Src_95C–βarr1_120C–V2Rpp–Fab30–Nb32 (Src–βarr1-CC)** (EMD-44881) (PDB 9BT8) |
|---|---|---|---|
| **Data collection and processing** | | | |
| Magnification | 81,000 | 81,000 | 81,000 |
| Voltage (kV) | 300 | 300 | 300 |
| Electron exposure (e–/Å$^2$) | 58.5 | 53.8 | 54.6 |
| Defocus range (μm) | -0.8 to -2.5 | -0.8 to -2.5 | -0.8 to -2.5 |
| Pixel size (Å) | 1.08 (collection) 1.3824 (final) | 1.08 (collection) 1.3824 (final) | 1.08 (collection) 1.44 (final) |
| Symmetry imposed | C1 | C1 | C1 |
| Initial particle projections (no.) | 200,270 | 5,607,258 | 9,770,378 |
| Final particle projections (no.) | 118,020 | 345,529 | 140,156 |
| Map resolution (Å) | 3.47 | 3.34 | 3.32 |
| FSC threshold | 0.143 | 0.143 | 0.143 |
| Map resolution range (Å) | 3.10-6.97 | 3.05-6.90 | 3.23-11.33 |
| **Refinement** | | | |
| Initial model used (PDB code) | 6NI2 | 4JQI | 8U7A |
| Model resolution (Å) | 3.47 | 3.3 | 3.2 |
| FSC threshold | 0.143 | 0.143 | 0.143 |
| Map sharpening *B* factor (Å$^2$) | -80.3 | -83.2 | -77.5 |
| Model composition | | | |
| Non-hydrogen atoms | 5627 | 4580 | 5713 |
| Protein residues | 720 | 609 | 728 |
| Ligands | 0 | 0 | 0 |
| *B* factors (Å$^2$) | | | |
| Protein (min/max/mean) | 20.22/103.43/47.54 | 8.88/113.35/37.29 | 10.51/115.11/53.64 |
| Ligand | --- | --- | |
| R.m.s. deviations | | | |
| Bond lengths (Å) | 0.005 | 0.004 | 0.004 |
| Bond angles (°) | 0.709 | 0.988 | 0.572 |
| Validation | | | |
| MolProbity score | 1.93 | 1.56 | 1.55 |
| Clashscore | 11.22 | 7.59 | 6.88 |
| Poor rotamers (%) | 0.16 | 0.21 | 0.32 |
| Ramachandran plot | | | |
| Favored (%) | 94.78 | 97.25 | 97.00 |
| Allowed (%) | 5.22 | 2.75 | 3.00 |
| Disallowed (%) | 0.00 | 0.00 | 0.00 |

## Supplementary Table 2. HDX data summary

| Data quality metric | βarr1 states | | | | SH3 states | | | |
|---|---|---|---|---|---|---|---|---|
| | βarr1 | βarr1 + SH3 | βarr1–V2rpp | βarr1–V2rpp+ SH3 | SH3 | SH3+ βarr1 | SH3+ V2rpp | SH3+ βarr1–V2rpp |
| HDX reaction details | 94% D2O, pH=7.5, 15°C | | | | 94% D2O, pH=7.5, 15°C | | | |
| HDX time course, sec | 300, 1000, 5000, MAX | | | | 300, 1000, 5000 | | | |
| HDX control sample | Maximally labeled βarr1 | | | | n/a | | | |
| Back-exchange level, Mean (Min; Max) | 31 (10; 53) | | | | n/a | | | |
| Number of peptides analyzed | 165 | 163 | 165 | 161 | 46 | 43 | 45 | 35 |
| Sequence coverage, % | 98.50 | 98.50 | 98.50 | 98.50 | 92.47 | 92.47 | 92.47 | 91.40 |
| Average peptide length | 12.65 | 12.65 | 12.65 | 12.61 | 11.13 | 10.91 | 10.93 | 11.11 |
| Peptide redundancy | 5.21 | 5.17 | 5.21 | 5.06 | 5.51 | 5.04 | 5.29 | 4.18 |
| Number of replicates | 3 (technical) | | | | 1 | | | |
| Repeatability (average standard deviation) | 0.084 | 0.097 | 0.119 | 0.108 | n/a | | | |
| Significant differences in HDX (delta HDX > X D) | 0.3 D | | 0.36 D | | n/a | | | |

## Supplementary Table 3. List of βarr1 peptides showing statistically significant differences in deuterium uptake* in the presence of SH3.

| βarr1 | βarr1–V2rpp |
|---|---|
| **Decrease in deuterium uptake in the presence of SH3 (cumulative ΔD over three time points, Da)** | |
| $^{34}$VDPVDGVVLVDPEY$^{47}$ **(-0.82)** | $^{86}$S**FPPAPE**DKKPLTRLQ$^{101}$ **(-1.3)** |
| $^{76}$**RKDLF**VANVQS**FPPAPE**DK$^{94}$ **(-0.87)** | $^{105}$IKKLGEHAYPFTFEI**PPNLP**SS$^{126}$ **(-2.0)** |
| $^{85}$QS**FPPAPE**DKKPLTRLQER$^{103}$ **(-1.1)** | $^{257}$EADDTVAPSSTFSKVYTLTPFLANNREKRGLALDGKLKHEDTNL$^{300}$ **(-3.5)** |
| $^{86}$S**FPPAPE**DKKPLTRLQ$^{101}$ **(-1.7)** | $^{282}$REKRGLALDGKLKHEDTNL$^{300}$ **(-1.63)** |
| $^{105}$IKKLGEHAYPFTFEI**PPNLP**SS$^{126}$ **(-1.2)** | $^{352}$MHPKPKEEPPHREVPESETPVDTNLI$^{377}$ **(-3.4)** |
| $^{155}$EEKIHKRNSVRLVIRKVQYAPERPGPQPTAET$^{186}$ **(-2.00)** | $^{353}$HPKPKEEPPHREVPESETPVDTNL$^{376}$ **(-1.60)** |
| $^{217}$NVHVTNNTNKTVKKIKISVRQYAD$^{240}$ **(-0.83)** | |
| $^{282}$REKRGLALDGKLKHEDTNL$^{300}$ **(-1.3)** | |
| $^{285}$RGLALDGKLKHEDTNLASSTLLREGA$^{310}$ **(-1.14)** | |
| **Increase in deuterium uptake in the presence of SH3 (cumulative ΔD over three time points, Da)** | |
| | $^{9}$FKKASPNGKL$^{18}$ **(1.3)** |

* Only regions that exhibited statistically significant differences in deuterium uptake (>0.3 Da for βarr1; >0.36 Da for βarr1–V2rpp) were considered relevant. Values shown in parentheses correspond to the cumulative ΔD measured over three time points (Da). Statistical analysis was performed using unpaired, two-tailed Welch's t-test. Residues interacting with SH3 in the structures are shown in bold, underlined. Residue numbers corresponding to native βarr1 numbering are shown as superscripts.

## Supplementary Table 4. List of SH3 peptides showing differences in deuterium uptake* in the presence of βarr1.

| SH3 | SH3+V2rpp |
|---|---|
| **Decrease in deuterium uptake in the presence of βarr1 or βarr1–V2rpp (cumulative ΔD over three time points, Da)** | |
| $^{101}$SFKKGERLQIVNNT**EGDW**$^{118}$ **(-1.01)** | $^{89}$L**YDYESR**TETDLSFKKGER$^{107}$**(-2.30)** |
| $^{102}$FKKGERLQIVNNT**EGDW**$^{118}$ **(-1.87)** | $^{101}$SFKKGERLQIVNNT**EGDW**$^{118}$ **(-2.61)** |
| $^{119}$WLAHSLTTGQTGYI**PSNY**VAPSD$^{141}$D **(-1.24)** | $^{102}$FKKGERLQIVNNT**EGDW**$^{118}$ **(-3.15)** |
| | $^{103}$KKGERLQIVNNT**EGDW**$^{118}$ **(-1.59)** |
| | $^{119}$WLAHSLTTGQTGYI**PSNY**VAPSD$^{141}$D **(-0.98)** |
| **Increase in deuterium uptake in the presence of βarr1 or βarr1–V2rpp (average ΔD; cumulative ΔD over three time points, Da)** | |
| $^{90}$**YDYESR**TETDLSF$^{102}$ **(2.44)** | $^{90}$**YDYESR**TETDLSF$^{102}$ **(1.76)** |
| $^{91}$**DYESR**TET$^{98}$ **(1.12)** | $^{91}$**DYESR**TET$^{98}$ **(1.11)** |
| | |

* Only regions that exhibited differences in deuterium uptake greater than 0.3 Da, supported by at least two overlapping peptides and observed at all time points, are shown. Values shown in parentheses correspond to the cumulative ΔD measured over three time points (Da). Residues in the proximity to βarr1 interface in the structures are shown in bold and underlined. Residue numbers corresponding to native Src numbering are shown as superscripts. The data presented are based on a single HDX experiment.

## Supplementary Table 5. Oligonucleotides and plasmids

| Original construct | Mutation | Forward and reverse oligonucleotides (5’→3’) | Generated plasmids |
|---|---|---|---|
| pGEX-4T1_βarr1-393_MinCys | A82C | gggaaggactgcacgttacacacaaacaggtctttgcg<br>cgcaaagacctgtttgtgtgtaacgtgcagtccttccc | pGEX-4T1_βarr1-393_MinCys_A82C |
| pGEX-4T1_βarr1-393_MinCys | F87C | gtggctaacgtgcagtcctgcccaccggcccctgaggacaag<br>cttgtcctcaggggccggtgggcaggactgcacgttagccac | pGEX-4T1_βarr1-393_MinCys_F87C |
| pGEX-4T1_βarr1-393_MinCys | P88C | gtcctcaggggccgggcagaaggactgcacgtt<br>aacgtgcagtccttctgcccggcccctgaggac | pGEX-4T1_βarr1-393_MinCys_P88C |
| pGEX-4T1_βarr1-393_MinCys | P89C | gtggctaacgtgcagtccttcccatgcgcccctgaggacaagaagccactgact<br>agtcagtggcttcttgtcctcaggggcgcatgggaaggactgcacgttagccac | pGEX-4T1_βarr1-393_MinCys_P89C |
| pGEX-4T1_βarr1-393_MinCys | A90C | ttcttgtcctcagggcacggtgggaaggactg<br>cagtccttcccaccgtgccctgaggacaagaa | pGEX-4T1_βarr1-393_MinCys_A90C |
| pGEX-4T1_βarr1-393_MinCys | P91C | gcttcttgtcctcacaggccggtgggaagg<br>ccttcccaccggcctgtgaggacaagaagc | pGEX-4T1_βarr1-393_MinCys_P91C |
| pGEX-4T1_βarr1-393_MinCys | E92C | tcagtggcttcttgtcgcaaggggccggtgggaag<br>cttcccaccggccccttgcgacaagaagccactga | pGEX-4T1_βarr1-393_MinCys_E92C |
| pGEX-4T1_βarr1-393_MinCys | D93C | gtcagtggcttcttgcactcaggggccggtgg<br>ccaccggcccctgagtgcaagaagccactgac | pGEX-4T1_βarr1-393_MinCys_D93C |
| pGEX-4T1_βarr1-393_MinCys | K94C | ccgagtcagtggcttgcagtcctcaggggccgg<br>ccggcccctgaggactgcaagccactgactcgg | pGEX-4T1_βarr1-393_MinCys_K94C |
| pGEX-4T1_βarr1-393_MinCys | K95C | gtagccgagtcagtgggcacttgtcctcaggggcc<br>ggcccctgaggacaagtgcccactgactcggctac | pGEX-4T1_βarr1-393_MinCys_K95C |
| pGEX-4T1_βarr1-393_MinCys | P96C | ccaccggcccctgaggacaagaagtgcctgactcggctacaagagcgactc<br>gagtcgctcttgtagccgagtcaggcacttcttgtcctcaggggccggtgg | pGEX-4T1_βarr1-393_MinCys_P96C |
| pGEX-4T1_βarr1-393_MinCys | Y113C | gtgaaggggcaggcatgctcgcccagc<br>gctgggcgagcatgcctgccccttcac | pGEX-4T1_βarr1-393_MinCys_Y113C |
| pGEX-4T1_βarr1-393_MinCys | P114C | gatctcaaaggtgaagcagtaggcatgctcgccc<br>gggcgagcatgcctactgcttcacctttgagatc | pGEX-4T1_βarr1-393_MinCys_P114C |
| pGEX-4T1_βarr1-393_MinCys | T116C | gcgagcatgcctaccccttctgctttgagatcccgccaaacc<br>ggtttggcgggatctcaaagcagaaggggtaggcatgctcgc | pGEX-4T1_βarr1-393_MinCys_T116C |
| pGEX-4T1_βarr1-393_MinCys | E118C | ggcgagcatgcctaccccttcaccttttgcatcccgccaaaccttccgagctcagtc<br>gactgagctcggaaggtttggcgggatgcaaaaggtgaagggtaggcatgctcgcc | pGEX-4T1_βarr1-393_MinCys_E118C |
| pGEX-4T1_βarr1-393_MinCys | P120C | tgagctcggaaggtttgggcagatctcaaaggtgaaggg<br>cccttcacctttgagatctgcccaaaccttccgagctca | pGEX-4T1_βarr1-393_MinCys_P120C |
| pGEX-4T1_βarr1-393_MinCys | P121C | tgactgagctcggaaggttgcacgggatctcaaaggtgaag<br>cttcacctttgagatcccgtgcaaccttccgagctcagtca | pGEX-4T1_βarr1-393_MinCys_P121C |
| pGEX-4T1_βarr1-393_MinCys | N122C | ctgagctcggaaggcatggcgggatctcaaagg<br>cctttgagatcccgccatgccttccgagctcag | pGEX-4T1_βarr1-393_MinCys_N122C |
| pET-28a_SH3 | L89C | gggactcgtagtcatagcaagccacgaaagtggtgac<br>gtcaccactttcgtggcttgctatgactacgagtcccg | pET-28a_SH3_L89C |
| pET-28a_SH3 | Y90C | gactcgtagtcacagagagccacgaaagtggtgacc<br>ggtcaccactttcgtggctctctgtgactacgagtc | pET-28a_SH3_Y90C |
| pET-28a_SH3 | Y92C | ccgggactcgcagtcatagagagccacgaa<br>ttcgtggctctctatgactgcgagtcccgg | pET-28a_SH3_Y92C |
| pET-28a_SH3 | R95C | ggacaagtccgtttcagtgcaggactcgtagtcatagag<br>ctctatgactacgagtcctgcactgaaacggacttgtcc | pET-28a_SH3_R95C |
| pET-28a_SH3 | E115C | gagccagccaccagtcaccgcacgtgttgttgacaatctgc<br>gcagattgtcaacaacacgtgcggtgactggtggctggctc | pET-28a_SH3_E115C |
| pET-28a_SH3 | D117C | gaatgagccagccaccagcaaccttccgtgttgttgac<br>gtcaacaacacggaaggttgctggtggctggctcattc | pET-28a_SH3_D117C |
| pET-28a_SH3 | W118C | gaatgagccagccagcagtcaccttccgt<br>acggaaggtgactgctggctggctcattc | pET-28a_SH3_W118C |
| pET-28a_SH3 | Y131C | tagttactggggatgcagcccgtctgtcctg<br>caggacagacgggctgcatccccagtaacta | pET-28a_SH3_Y131C |
| pET-28a_SH3 | P133C | gcgcgacatagttactgcagatgtagcccgtctgtc<br>gacagacgggctacatctgcagtaactatgtcgcgc | pET-28a_SH3_P133C |
| pET-28a_SH3 | S134C | cgcgacatagttacaggggatgtagcccg<br>cgggctacatcccctgtaactatgtcgcg | pET-28a_SH3_P134C |
| pET-28a_SH3 | N135C | gagggcgcgacatagcaactggggatgtagcc<br>ggctacatccccagttgctatgtcgcgccctc | pET-28a_SH3_N135C |

| | | | |
|---|---|---|---|
| pET-28a_SH3 | Y136C | gggcgcgacacagttactggggatgtagcc<br>ggctacatccccagtaactgtgtcgcgccc | pET-28a_SH3_Y136C |
| pET-28a_SH3 | A138C | cctggatctagtctgaggggcagacatagttactggggatg<br>catccccagtaactatgtctgcccctcagactagatccagg | pET-28a_SH3_A138C |
| pET-28a_SH3-FLAG | Y90A | ccgggactcgtagtcagcgagagccacgaaagtg<br>cactttcgtggctctcgctgactacgagtcccgg | pET-28a_SH3-FLAG_Y90A |
| pET-28a_SH3-FLAG_Y90A /<br>pET-28a_SH3-FLAG_Y92C | Y136A | gagggcgcgacagcgttactggggatgtagcc<br>ggctacatccccagtaacgctgtcgcgccctc | pET-28a_SH3-FLAG_Y90A_Y136A/<br>pET-28a_SH3-FLAG_Y92C_Y136A |
| pET-28a_SH3-FLAG_Y92C_Y136A | W118A | ggaatgagccagccacgcgtcaccttccgtgttg<br>caacacggaaggtgacgcgtggctggctcattcc | pET-28a_SH3-FLAG_Y92C_W118A_Y136A |
| pET-28a_Src | C185S | aacggagaggctataggcaccttttgtcgtctc<br>gagacgacaaaaggtgcctatagcctctccgtt | pET-28a_Src_C185S |
| pET-28a_Src_C185S | C238S | gtcaggcggtggctcaagccatcagca<br>tgctgatggcttgagccaccgcctgac | pET-28a_Src_C185S_C238S |
| pET-28a_Src_C185S_C238S | C245V | gcttggacgtggggacgacgttggtcaggc<br>gcctgaccaacgtcgtccccacgtccaagc | pET-28a_Src_C185S_C238S_C245V |
| pET-28a_Src_C185S_C238S_C245V | C277S | cctctccaaagctgccctgccccag<br>ctggggcagggcagctttggagagg | pET-28a_Src_C185S_C238S_C245V_C277S |
| pET-28a_Src_C185S_C238S_C245V_C277S | C400S | gtcagccaccttgctcaccaggttctccc<br>gggagaacctggtgagcaaggtggctgac | pET-28a_Src_C185S_C238S_C245V_C277S_C400S |
| pET-28a_Src_C185S_C238S_C245V_C277S_C400S | R95C | ggacaagtccgtttcagtgcaggactcgtagtcatagag<br>ctctatgactacgagtcctgcactgaaacggacttgtcc | pET-28a_Src_C185S_C238S_C245V_C277S_C400S_R95C |
| pET-28a_Src_C185S_C238S_C245V_C277S_C400S | E115C | gagccagccaccagtcaccgcacgtgttgttgacaatctgc<br>gcagattgtcaacaacacgtgcggtgactggtggctggctc | pET-28a_Src_C185S_C238S_C245V_C277S_C400S_E115C |
| pET-28a_Src_C185S_C238S_C245V_C277S_C400S | N135C | gagggcgcgacatagcaactggggatgtagcc<br>ggctacatccccagttgctatgtcgcgccctc | pET-28a_Src_C185S_C238S_C245V_C277S_C400S_N135C |
| pcDNA3_βarr1-HA /<br>pGEX-4T1_βarr1 | P88G-P91G | cttcttgtcctcaccggccggtccgaaggactgcacg<br>cgtgcagtccttcggaccggccggtgaggacaagaag | pcDNA3_βarr1-HA_P88G_P91G /<br>pGEX-4T1_βarr1_P88G_P91G |
| pcDNA3_βarr1-HA/<br>pGEX-4T1_βarr1 | P121E-P124G | gttgcaatgtgactgagcacccaaggttctccgggatctcaaag<br>gtgaagg<br>ccttcacctttgagatcccggagaaccttgggtgctcagtcacatt<br>gcaac | pcDNA3_βarr1-HA_P121E_P124G /<br>pGEX-4T1_βarr1_P121E_P124G |
| pcDNA3_βarr1-HA /<br>pGEX-4T1_βarr1 | F80A | ggactgcacgttagccacagccaggtctttgcgaaaagtc<br>gacttttcgcaaagacctggctgtggctaacgtgcagtcc | pcDNA3_βarr1-HA_F80A /<br>pGEX-4T1_βarr1_F80A |
| pcDNA3_βarr1-HA_P121E_P124G<br>/<br>pGEX-4T1_βarr1_P121E_P124G | P88G-P91G | cttcttgtcctcaccggccggtccgaaggactgcacg<br>cgtgcagtccttcggaccggccggtgaggacaagaag | pcDNA3_βarr1-HA_ P88G_P91G<br>_P121E_P124G /<br>pGEX-4T1_βarr1_<br>P88G_P91G_P121E_P124G |
| pGEX-4T1_βarr1_P121E_P124G | F75A | caggtctttgcgagcagtcagacccaagacatccaggtct<br>agacctggatgtcttgggtctgactgctcgcaaagacctg | pGEX-4T1_βarr1_P121E_P124G_F75A |
| pGEX-4T1_βarr1_P121E_P124G_F75A | I314A | aaacaatgatacccagggcttcacggttggcgccttcc<br>ggaaggcgccaaccgtgaagccctgggtatcattgttt | pGEX-4T1_βarr1_P121E_P124G_F75A_I314A |
| pGEX-4T1_βarr1_P121E_P124G_F75A | N122A | gactgagcacccaagggcctccgggatctcaaag<br>ctttgagatcccggaggcccttgggtgctcagtc | pGEX-4T1_βarr1_P121E_P124G_F75A_N122A |
| pGEX-4T1_βarr1 | E176stop | ggccaggcctctaaggggcatattgaacc<br>ggttcaatatgccccttagaggcctggcc | pGEX-4T1_βarr1_ E176stop |

# References


1. Tan, Y.Z. et al. Addressing preferred specimen orientation in single-particle cryo-EM through tilting. *Nature Methods* **14**, 793-+ (2017).
2. Zhang, Y. & Skolnick, J. Scoring function for automated assessment of protein structure template quality. *Proteins* **57**, 702-10 (2004).
