# Supplementary material for: Mechanism of beta-arrestin 1 mediated Src activation via Src SH3 domain revealed by cryo-electron microscopy
Source: Nat Commun. 2026 Feb 20;17:2973. doi: 10.1038/s41467-026-69884-1 (PMC13035853; doi:10.1038/s41467-026-69884-1)
Supplement: Supplementary file 2 — Description of Additional Supplementary Files [file 41467_2026_69884_MOESM2_ESM.pdf]

Description of Additional Supplementary Files

File Name: Supplementary Video 1

Description: SH3–βarr1-CC complex, cryo-EM map and model colored by subunit (green, βarr1; magenta, SH3; red, V2Rpp; blue, Fab30; yellow, Nb32). Interacting residues are shown as sticks, hydrogen bonds are indicated as dashed lines. Map contour level is 0.75.

File Name: Supplementary Video 2

Description: SH3–βarr1-N complex, cryo-EM map and model colored by subunit (green, βarr1; magenta, SH3; red, V2Rpp; blue, Fab30). Map contour level is 0.52.

File Name: Supplementary Video 3

Description: βarr1 activates Src by SH3 domain displacement. Inactive Src is constrained by the intramolecular interactions between SH3 (magenta), SH2 (cyan) and SH1 (blue). βarr1 (green, the βarr1-CC site is shown) binds the aromatic surface of SH3 and displaces the SH2-SH1 linker (yellow) releasing the catalytic (SH1) domain of Src. Transparent coloring indicates flexible regions, including the inducible snap lock, C-terminal tail, and liberated SH1 domain. The displaced SH2-SH1 linker is shown as dashed line.

File Name: Supplementary Data 1

Description: HDX data table for βarr1 in the presence of SH3. Residue numbering corresponds to the βarr1 construct used, which is offset by –8 residues relative to the native βarr1 sequence.

File Name: Supplementary Data 2

Description: HDX data table for V2Rpp-activated βarr1 in the presence of SH3. Residue numbering corresponds to the βarr1 construct used, which is offset by –8 residues relative to the native βarr1 sequence.

File Name: Supplementary Data 3

Description: HDX data table for SH3 in the presence of βarr1. Residue numbering corresponds to the SH3 construct used, which is offset by 56 residues relative to the native Src sequence.

File Name: Supplementary Data 4

Description: HDX data table for SH3+V2Rpp in the presence of V2Rpp-activated βarr1. Residue numbering corresponds to the SH3 construct used, which is offset by 56 residues relative to the native Src sequence.

File Name: Supplementary Data 5

Description: The individual deuterium uptake plots for all identified peptides (βarr1; βarr1+SH3; βarr1–V2Rpp; βarr1–V2Rpp+SH3). Residue numbering corresponds to the βarr1 construct used, which is offset by –8 residues relative to the native βarr1 sequence.

File Name: Supplementary Data 6

Description: The individual deuterium uptake plots for all identified peptides (SH3; βarr1+SH3; SH3+V2Rpp; SH3+βarr1–V2Rpp). Residue numbering corresponds to the SH3 construct used, which is offset by 56 residues relative to the native Src sequence.
