# Supplementary material for: Mechanism of beta-arrestin 1 mediated Src activation via Src SH3 domain revealed by cryo-electron microscopy
Source: Nat Commun. 2026 Feb 20;17:2973. doi: 10.1038/s41467-026-69884-1 (PMC13035853; doi:10.1038/s41467-026-69884-1)

BARR1-393_MINCYS 1-16: GSPEFPGRLGDKGTRV (#1)

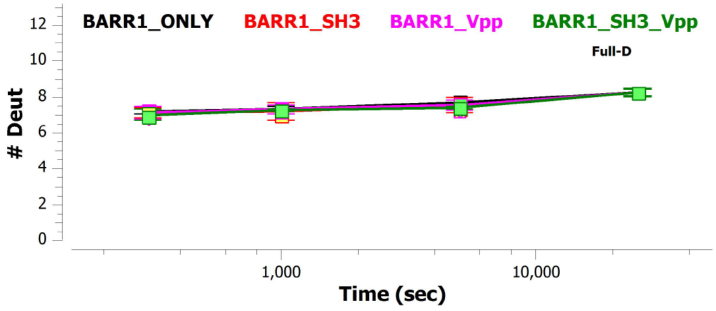

BARR1-393_MINCYS 1-26: GSPEFPGRLGDKGTRVFKKASPNGKL (#2)

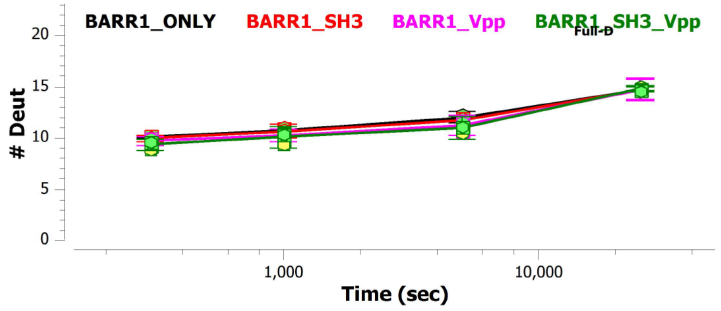

BARR1-393_MINCYS 3-16: PEFPGRLGDKGTRV (#3)

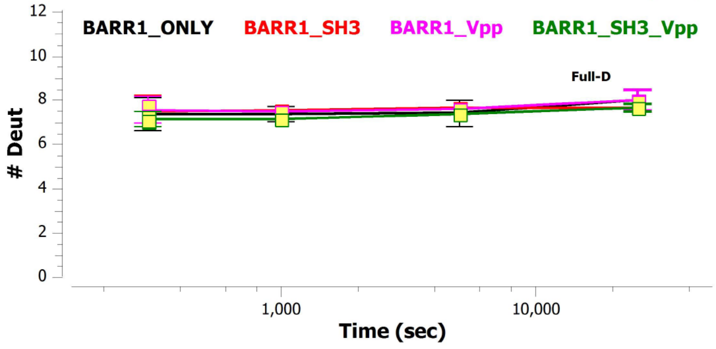

BARR1-393_MINCYS 13-20: GTRVFKKA (#4)

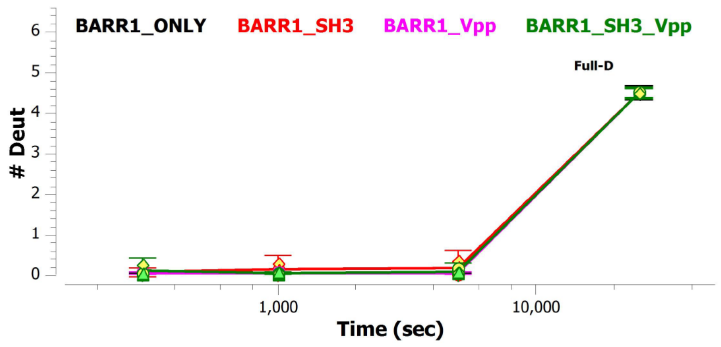

BARR1-393_MINCYS 17-26: FKKASPNGKL (#5)

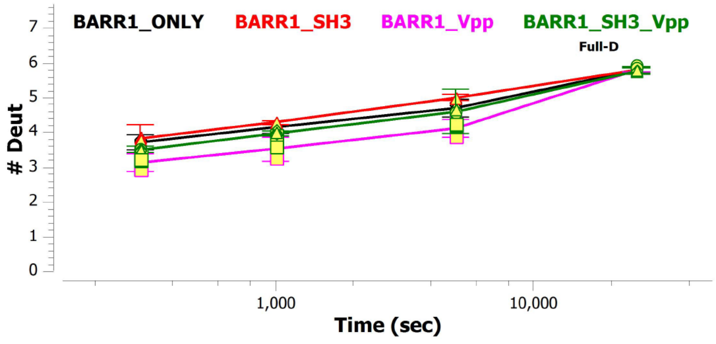

BARR1-393_MINCYS 17-29: FKKASPNGKLTVY (#6)

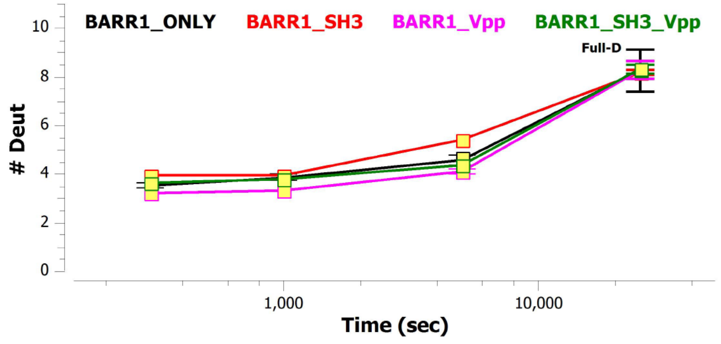

BARR1-393_MINCYS 27-41: TVYLGKRDFVDHIDL (#7)

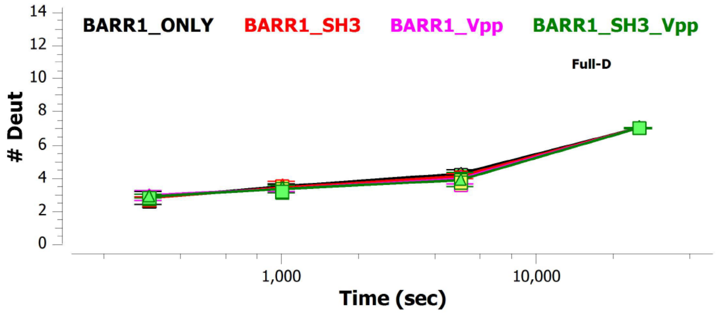

BARR1-393_MINCYS 27-47: TVYLGKRDFVDHIDLVDPVDG (#8)

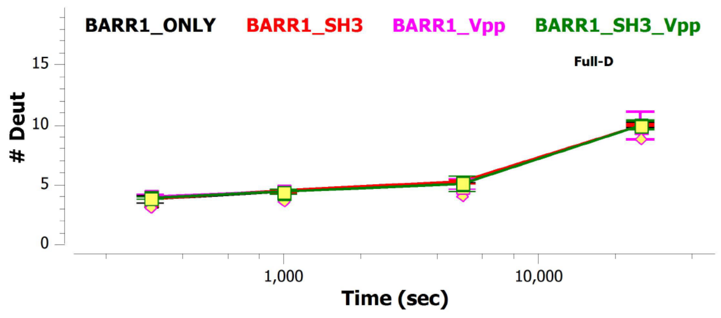

BARR1-393_MINCYS 29-41: YLGKRDFVDHIDL (#9)

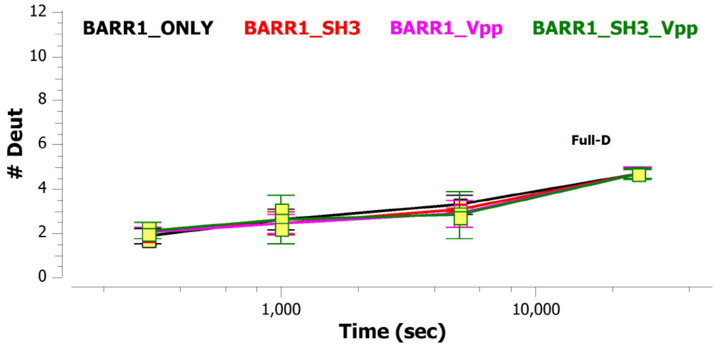

BARR1-393_MINCYS 30-41: LGKRDFVDHIDL (#10)

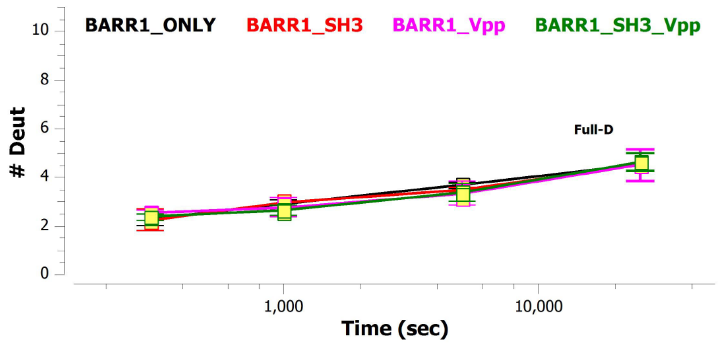

BARR1-393_MINCYS 30-50: LGKRDFVDHIDLVDPVDGVVL (#11)

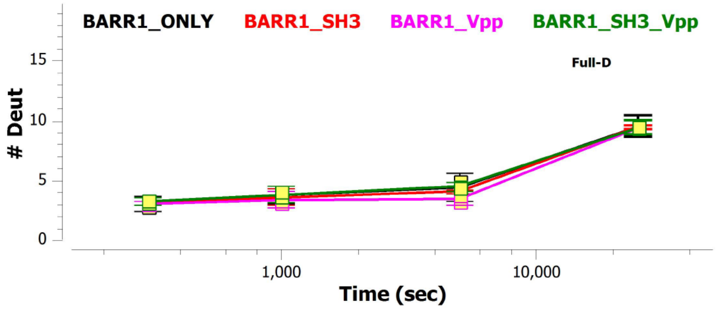

BARR1-393_MINCYS 42-47: VDPVDG (#12)

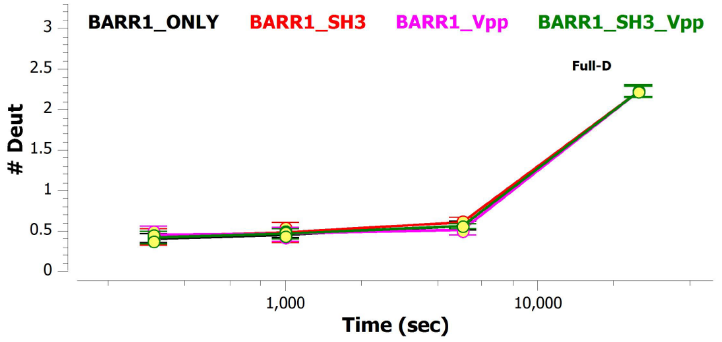

BARR1-393_MINCYS 42-49: VDPVDGVV (#13)

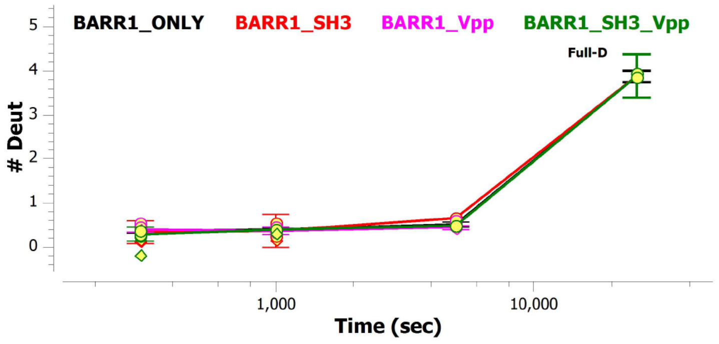

BARR1-393_MINCYS 42-50: VDPVDGVVL (#14)

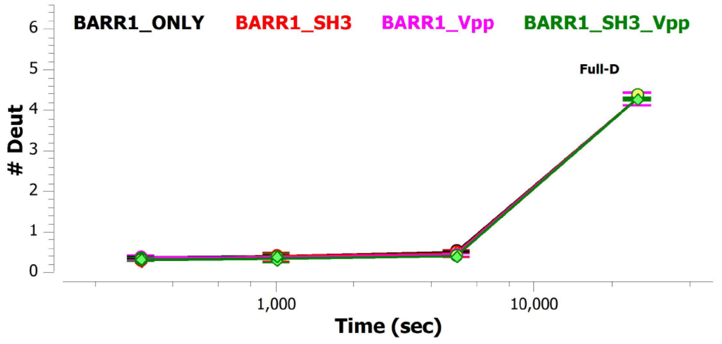

BARR1-393_MINCYS 42-55: VDPVDGVVLVDPEY (#15)

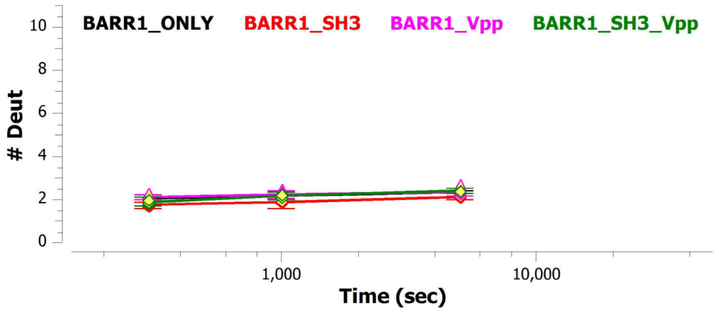

BARR1-393_MINCYS 43-49: DPVDGVV (#16)

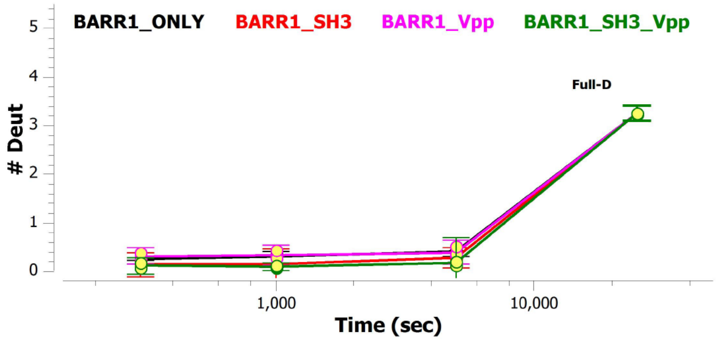

BARR1-393_MINCYS 48-55: VVLVDPEY (#17)

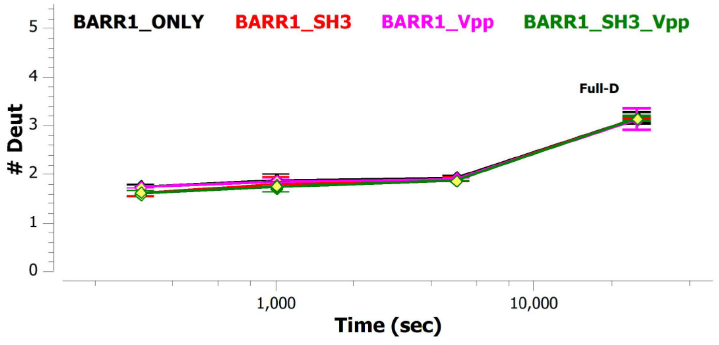

BARR1-393_MINCYS 48-56: VVLVDPEYL (#18)

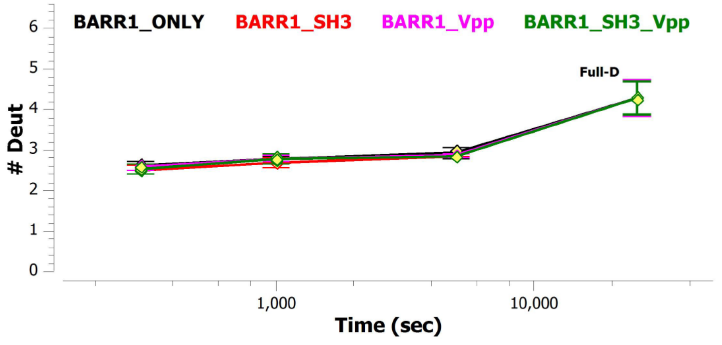

BARR1-393_MINCYS 48-65: VVLVDPEYLKERRVYVTL (#19)

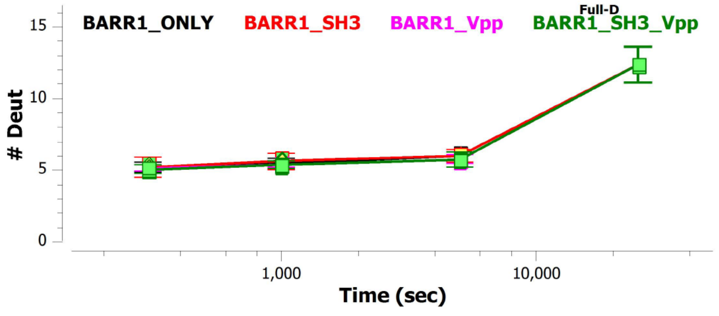

BARR1-393_MINCYS 48-66: VVLVDPEYLKERRVYVTLT (#20)

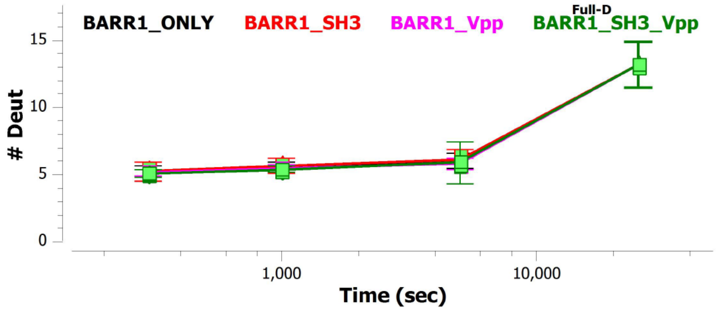

BARR1-393_MINCYS 50-55: LVDPEY (#21)

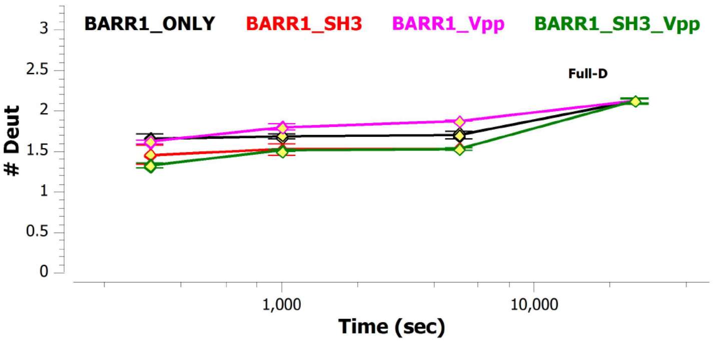

BARR1-393_MINCYS 50-64: LVDPEYLKERRVYVT (#22)

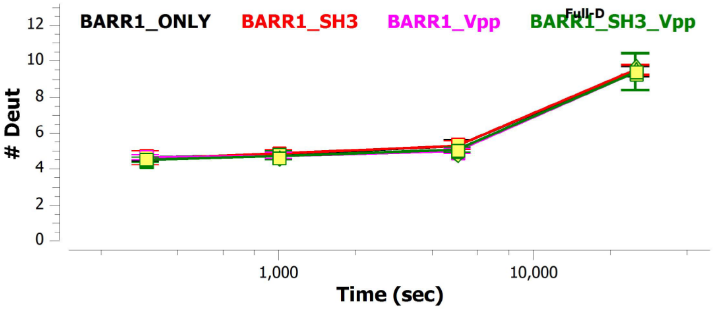

BARR1-393_MINCYS 50-65: LVDPEYLKERRVYVTL (#23)

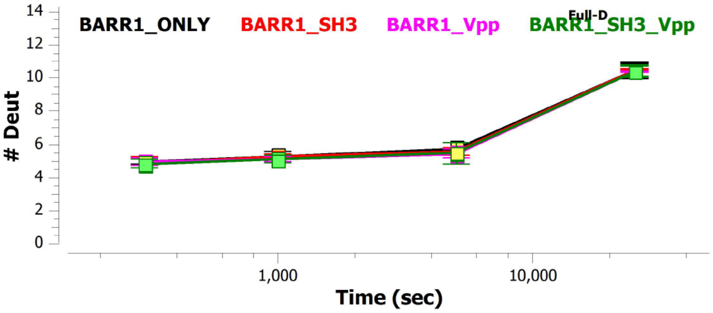

BARR1-393_MINCYS 51-55: VDPEY (#24)

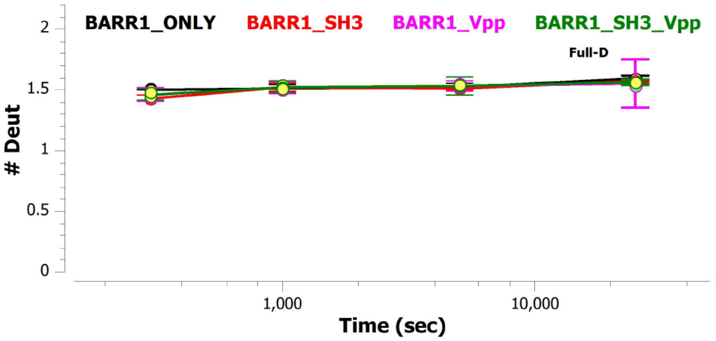

BARR1-393_MINCYS 51-62: VDPEYLKERRVY (#25)

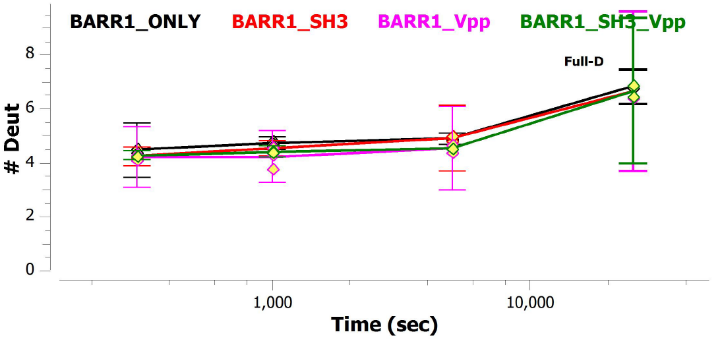

BARR1-393_MINCYS 52-67: DPEYLKERRVYVTLTV (#26)

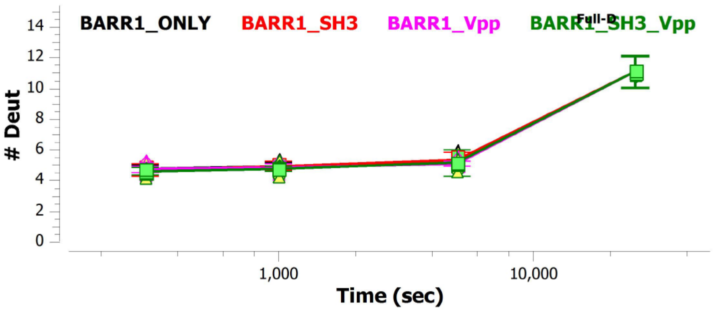

BARR1-393_MINCYS 56-62: LKERRVY (#27)

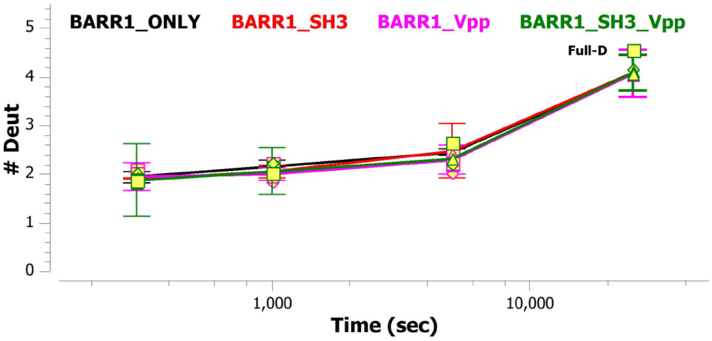

BARR1-393_MINCYS 56-65: LKERRVYVTL (#28)

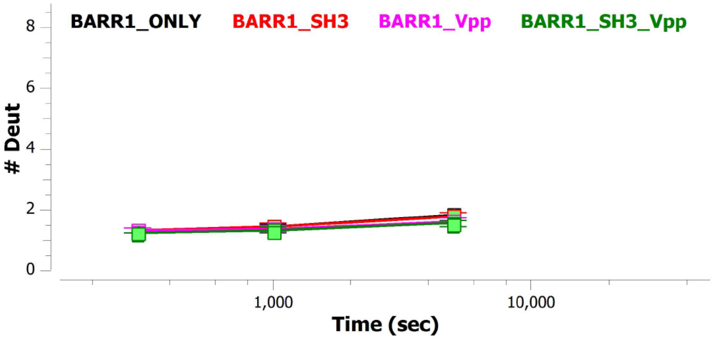

BARR1-393_MINCYS 56-66: LKERRVYVTLT (#29)

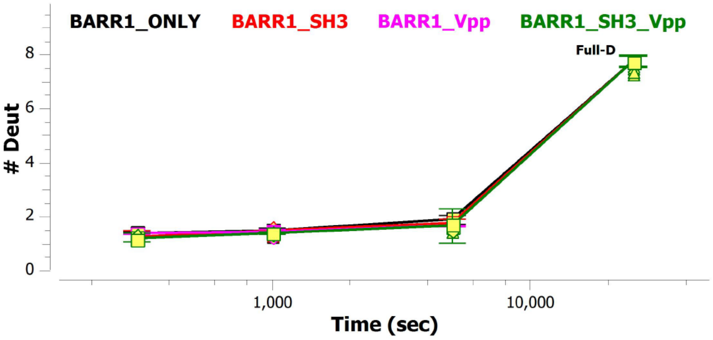

BARR1-393_MINCYS 57-65: KERRVYVTL (#30)

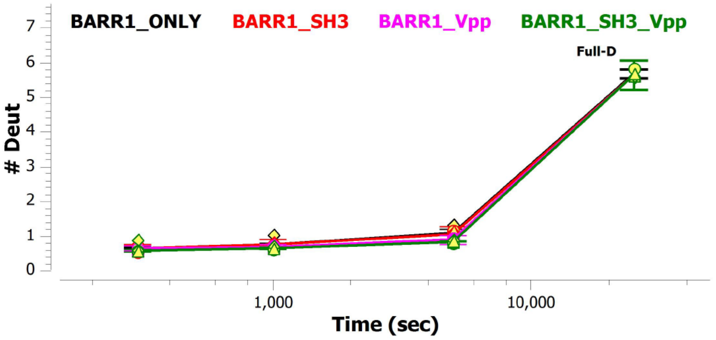

BARR1-393_MINCYS 61-79: VYVTLTVAFRYGREDLDVL (#31)

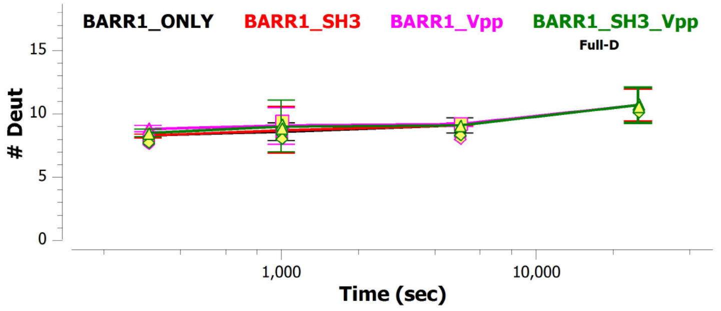

BARR1-393_MINCYS 63-66: VTLT (#32)

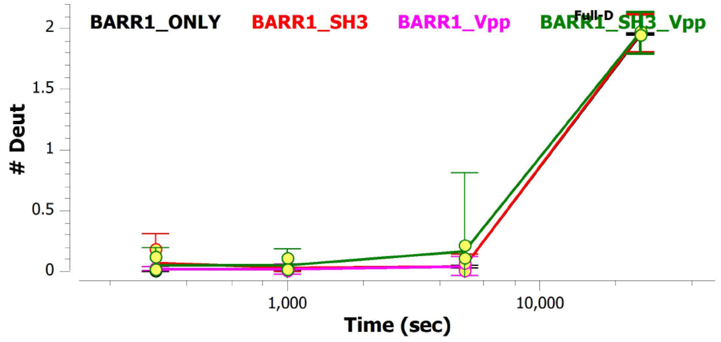

BARR1-393_MINCYS 65-72: LTVAFRYG (#33)

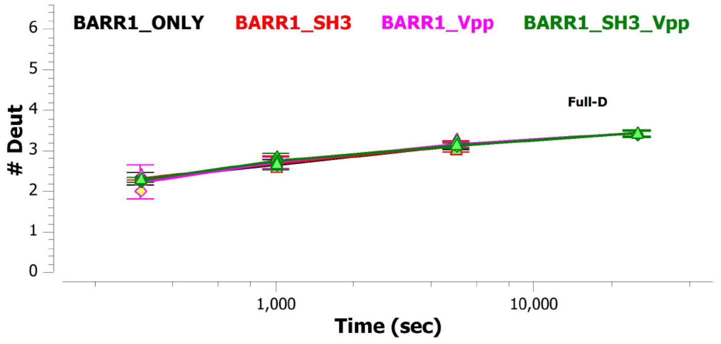

BARR1-393_MINCYS 65-76: LTVAFRYGREDL (#34)

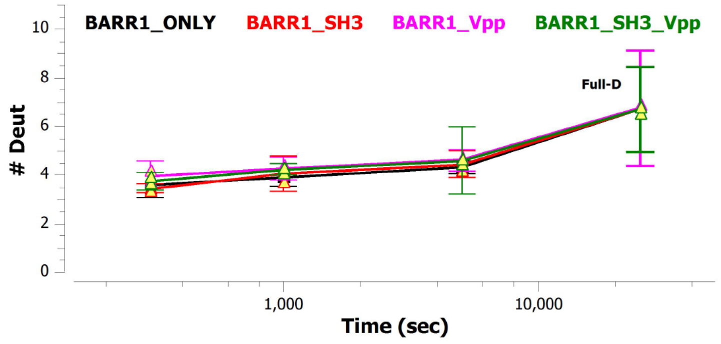

BARR1-393_MINCYS 65-79: LTVAFRYGREDLDVL (#35)

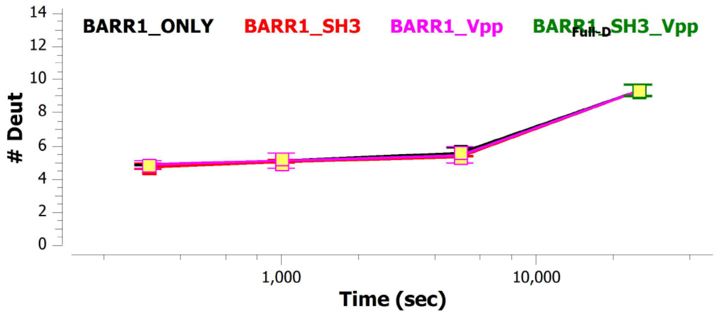

BARR1-393_MINCYS 66-76: TVAFRYGREDL (#36)

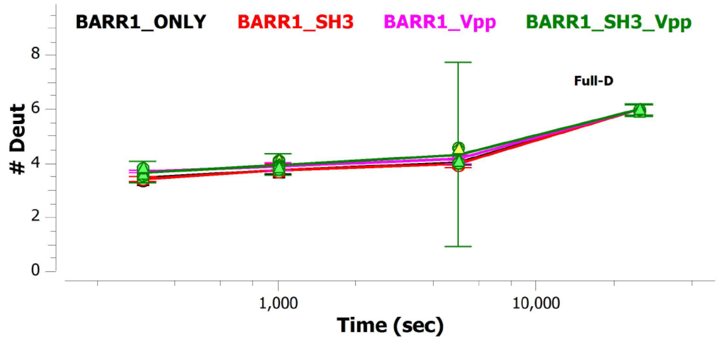

BARR1-393_MINCYS 66-78: TVAFRYGREDLDV (#37)

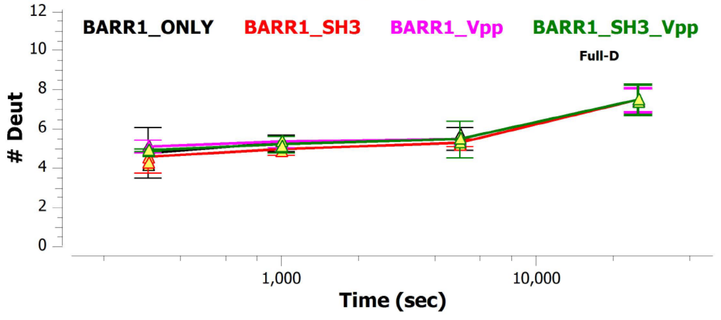

BARR1-393_MINCYS 67-76: VAFRYGREDL (#38)

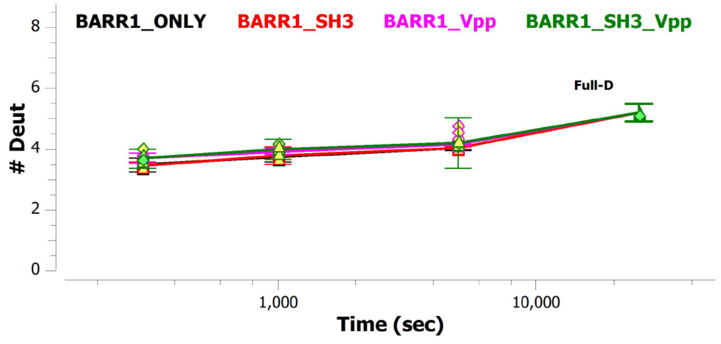

BARR1-393_MINCYS 69-76: FRYGREDL (#39)

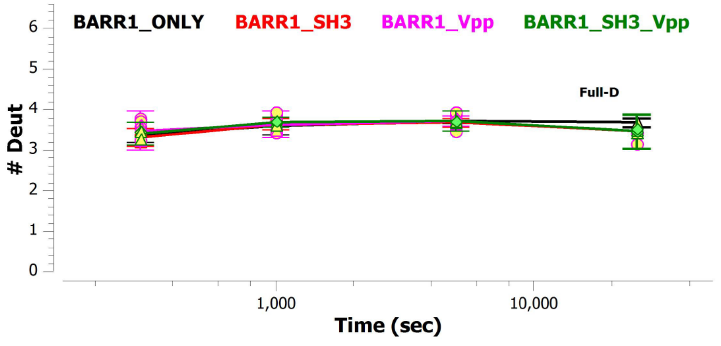

BARR1-393_MINCYS 70-76: RYGREDL (#40)

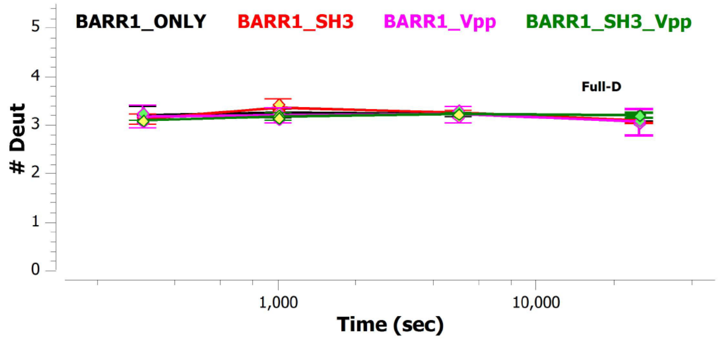

BARR1-393_MINCYS 74-89: EDLDVLGLTFRKDLFV (#41)

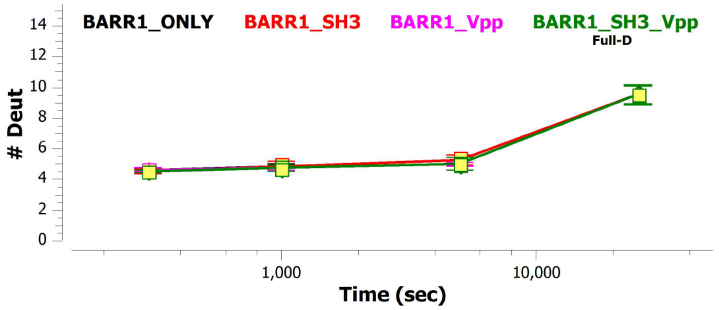

BARR1-393_MINCYS 76-109: LDVLGLTFRKDLFVANVQSFPPAPEDKKPLTRLQ (#42)

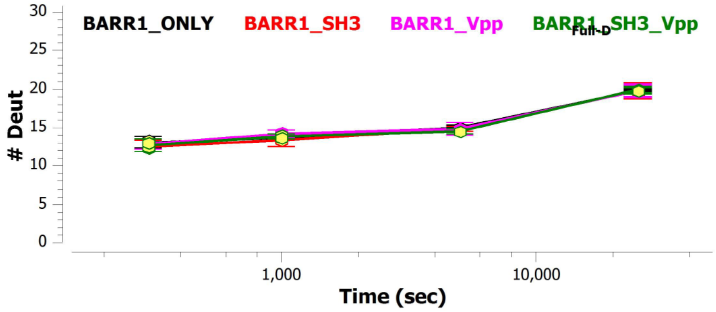

BARR1-393_MINCYS 77-83: DVLGLTF (#43)

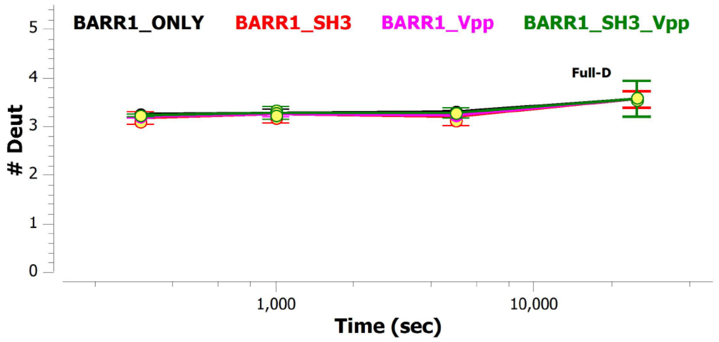

BARR1-393_MINCYS 77-87: DVLGLTFRKDL (#44)

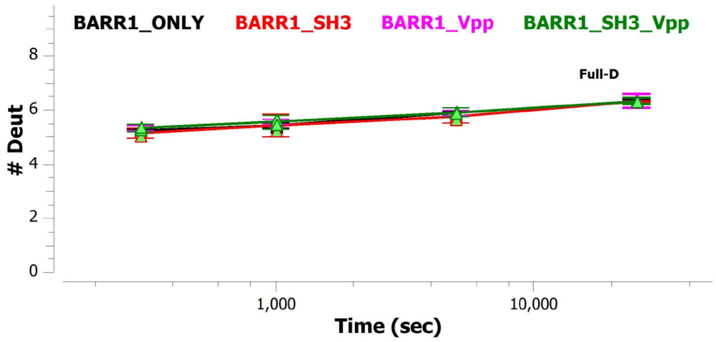

BARR1-393_MINCYS 77-88: DVLGLTFRKDLF (#45)

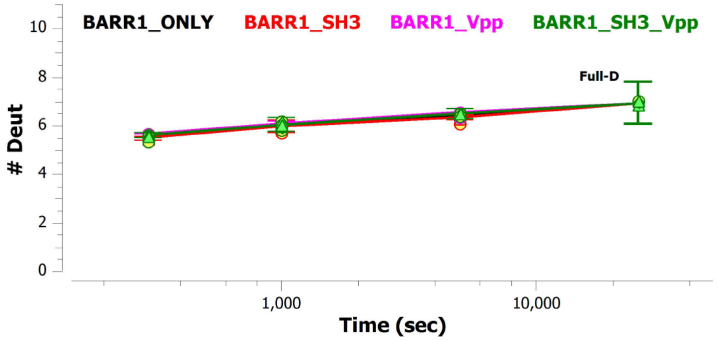

BARR1-393_MINCYS 80-88: GLTFRKDLF (#46)

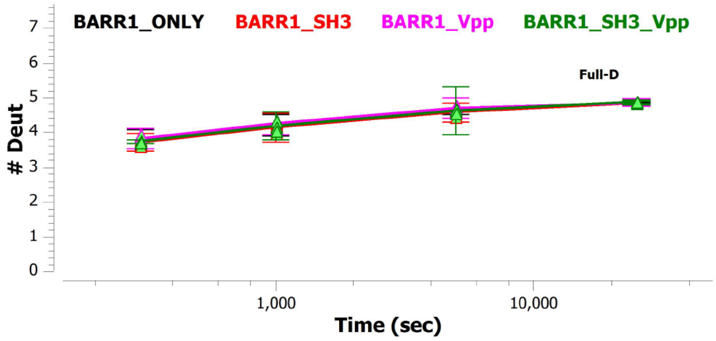

BARR1-393_MINCYS 81-88: LTFRKDLF (#47)

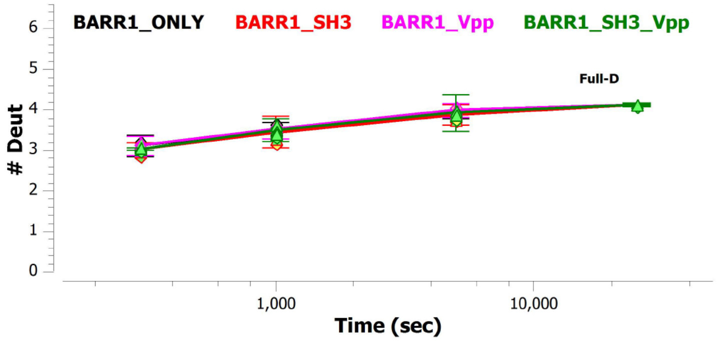

BARR1-393_MINCYS 81-90: LTFRKDLFVA (#48)

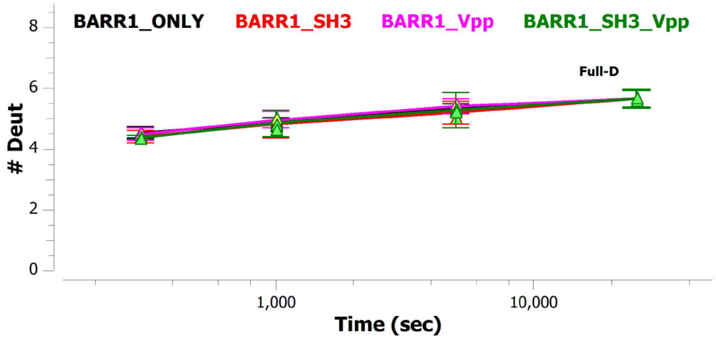

BARR1-393_MINCYS 81-93: LTFRKDLFVANVQ (#49)

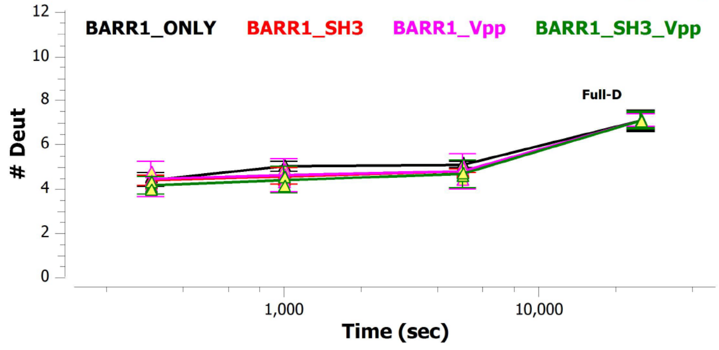

BARR1-393_MINCYS 83-87: FRKDL (#50)

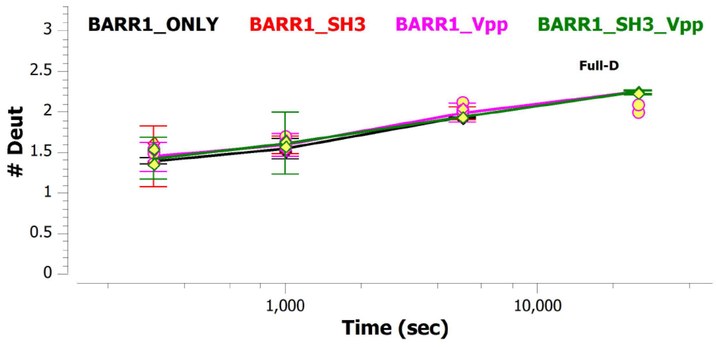

BARR1-393_MINCYS 84-102: RKDLFVANVQSFPPAPEDK (#51)

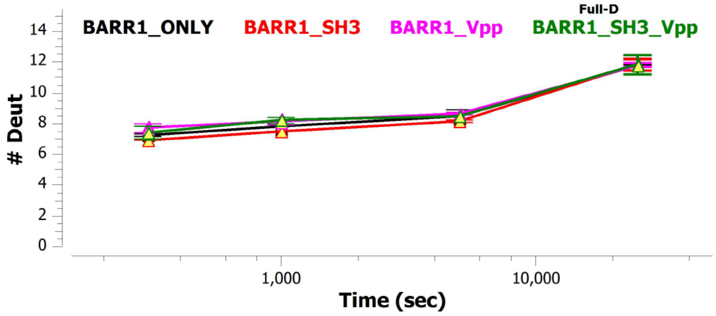

BARR1-393_MINCYS 83-88: FRKDLF (#52)

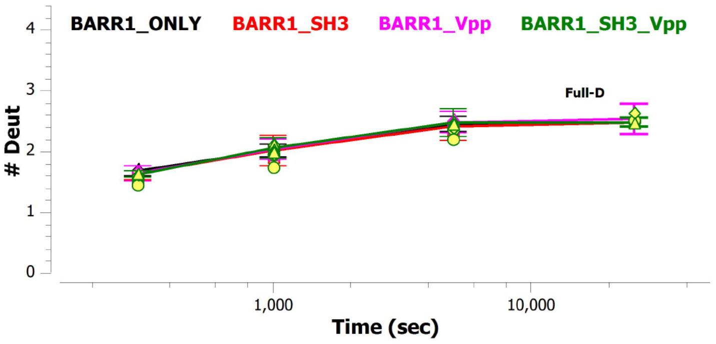

BARR1-393_MINCYS 88-108: FVANVQSFPPAPEDKKPLTRL (#53)

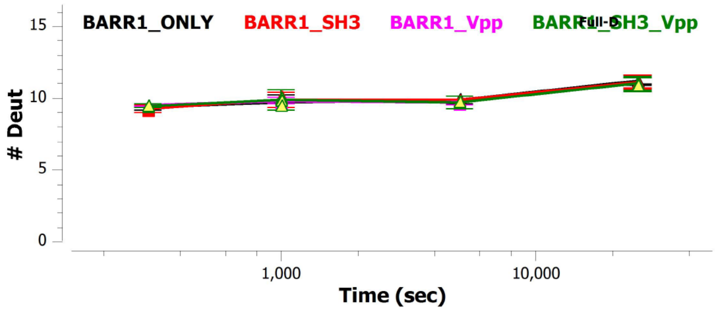

BARR1-393_MINCYS 89-108: VANVQSFPPAPEDKKPLTRL (#54)

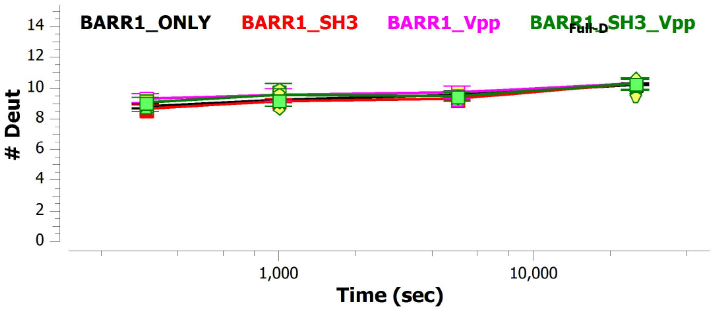

BARR1-393_MINCYS 89-112: VANVQSFPPAPEDKKPLTRLQERL (#55)

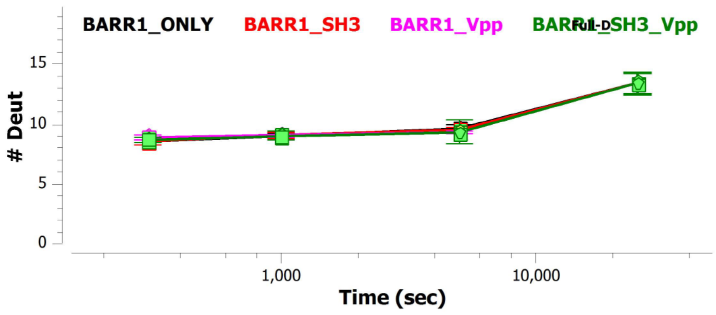

BARR1-393_MINCYS 93-111: QSFPPAPEDKKPLTRLQER (#56)

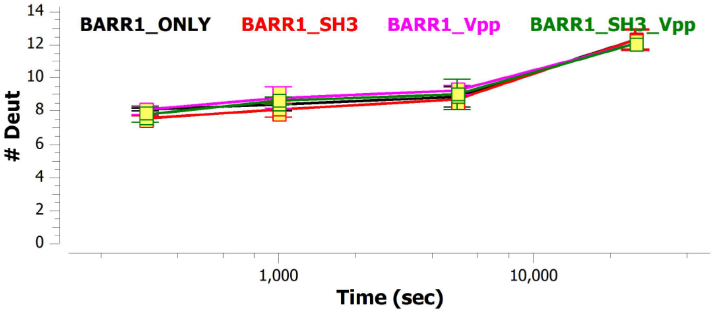

BARR1-393_MINCYS 94-109: SFPPAPEDKKPLTRLQ (#57)

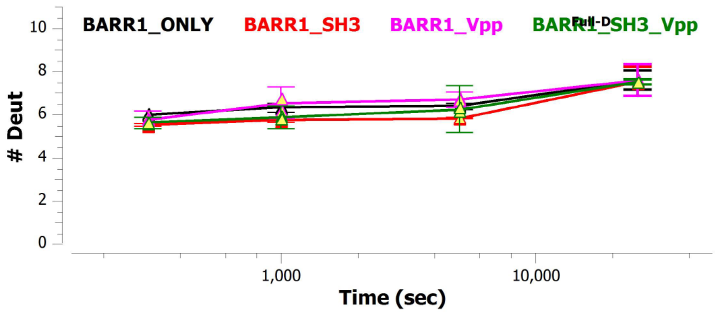

BARR1-393_MINCYS 108-111: LQER (#58)

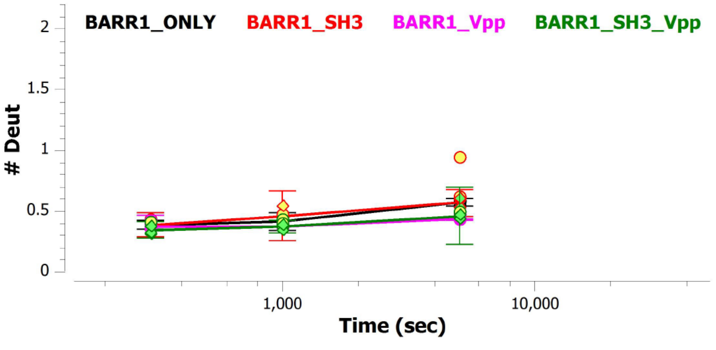

BARR1-393_MINCYS 109-124: QERLIKKLGEHAYPFT (#59)

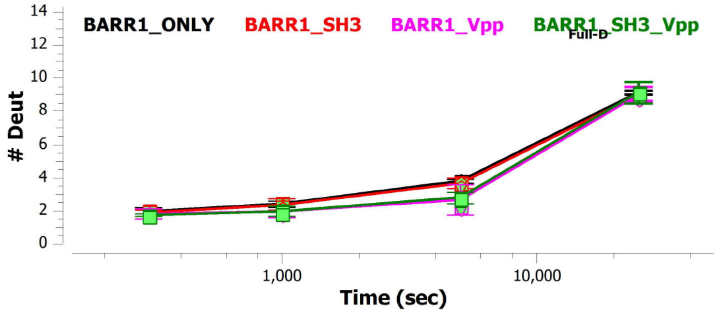

BARR1-393_MINCYS 113-124: IKKLGEHAYPFT (#60)

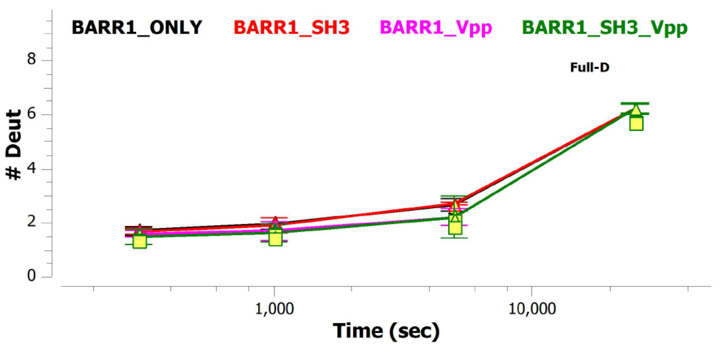

BARR1-393_MINCYS 113-134: IKKLGEHAYPFTFEIPPNLPSS (#61)

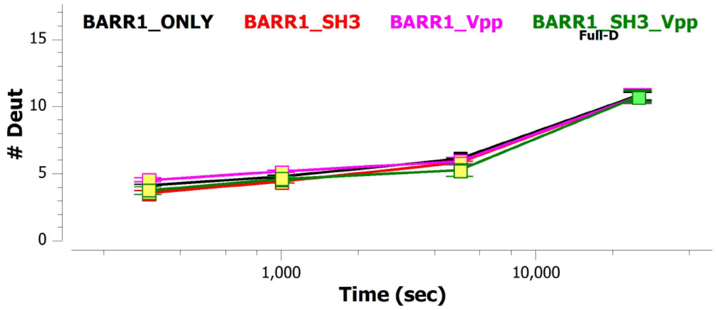

BARR1-393_MINCYS 125-134: FEIPPNLPSS (#62)

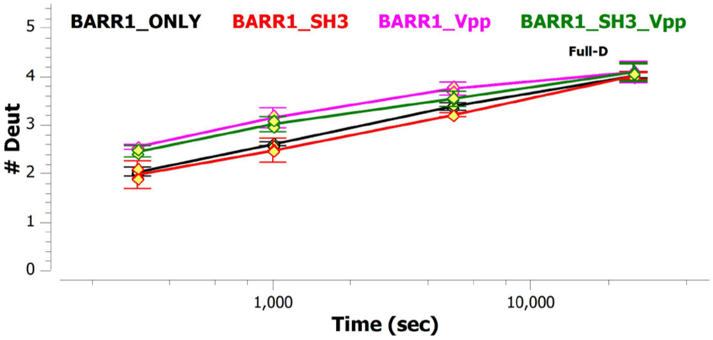

BARR1-393_MINCYS 131-145: LPSSVTLQPGPEDTG (#63)

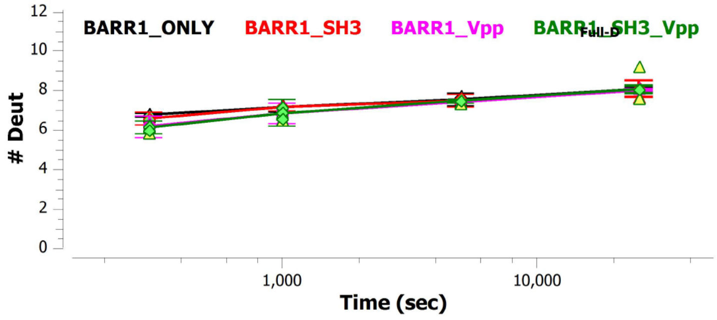

BARR1-393_MINCYS 132-148: PSSVTLQPGPEDTGKAL (#64)

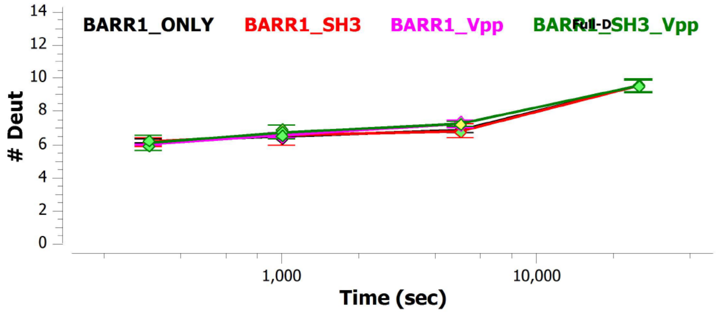

BARR1-393_MINCYS 136-145: TLQPGPEDTG (#65)

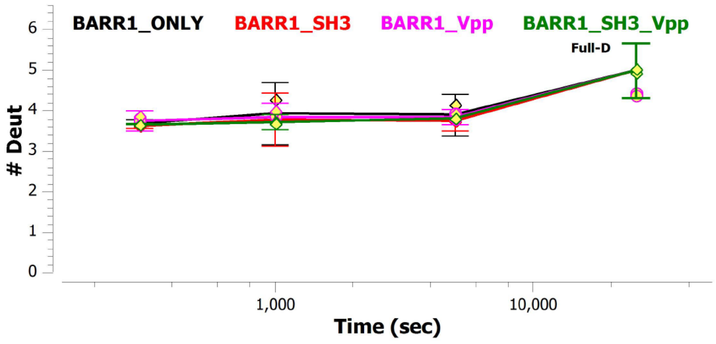

BARR1-393_MINCYS 137-149: LQPGPEDTGKALG (#66)

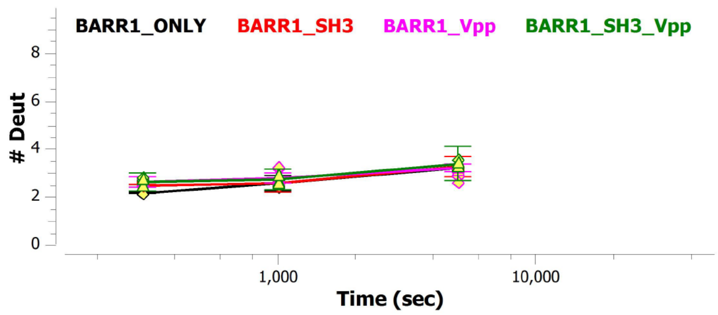

BARR1-393_MINCYS 153-156: EVKA (#67)

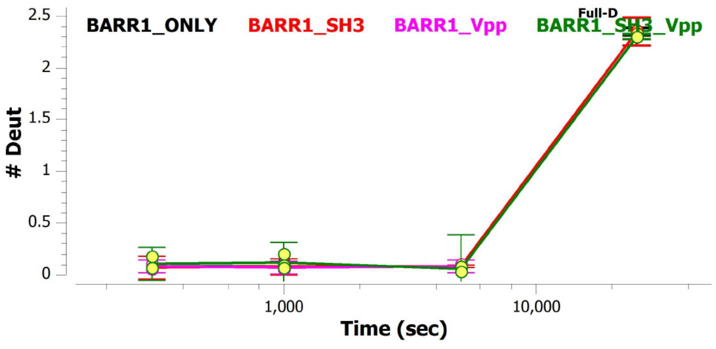

BARR1-393_MINCYS 153-157: EVKAF (#68)

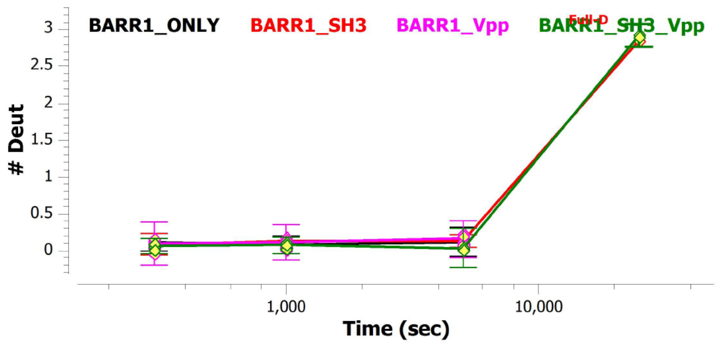

BARR1-393_MINCYS 153-161: EVKAFVAEN (#69)

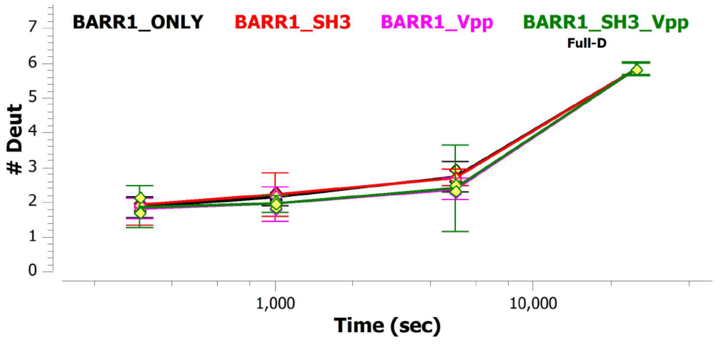

BARR1-393_MINCYS 153-162: EVKAFVAENL (#70)

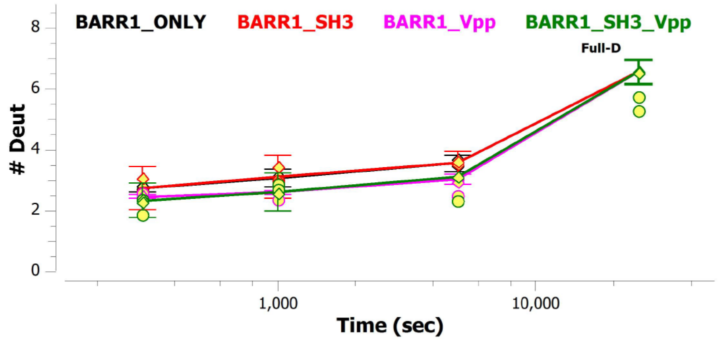

BARR1-393_MINCYS 153-169: EVKAFVAENLEEKIHKR (#71)

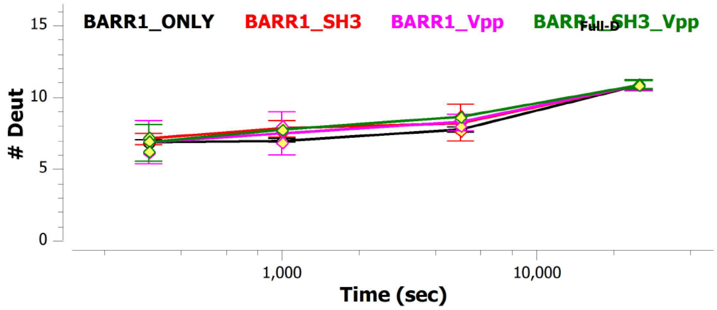

BARR1-393_MINCYS 157-162: FVAENL (#72)

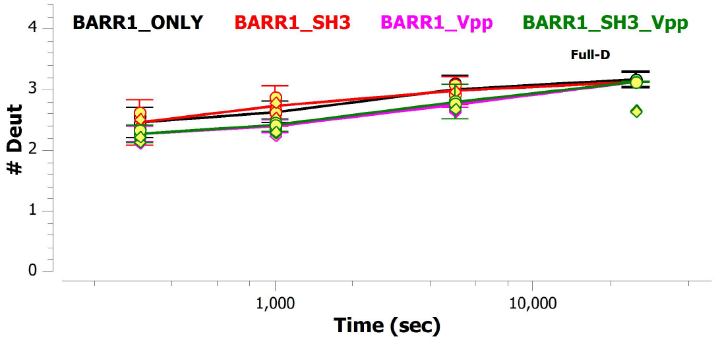

BARR1-393_MINCYS 158-162: VAENL (#73)

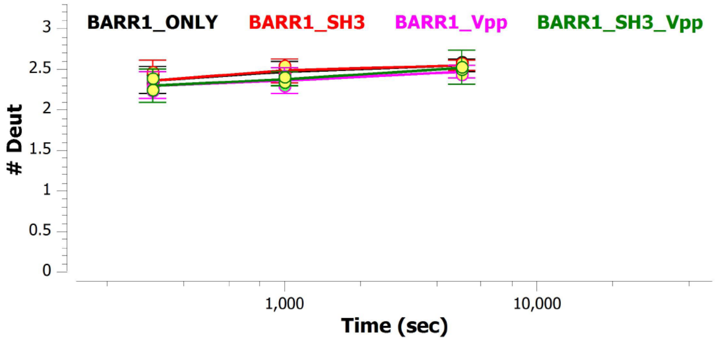

BARR1-393_MINCYS 158-174: VAENLEEKIHKRNSVRL (#74)

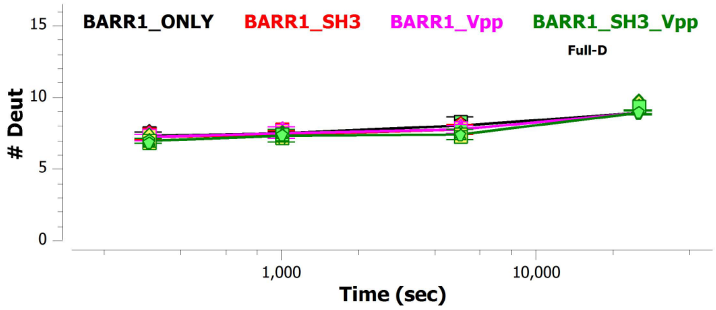

BARR1-393_MINCYS 162-173: LEEKIHKRNSVR (#75)

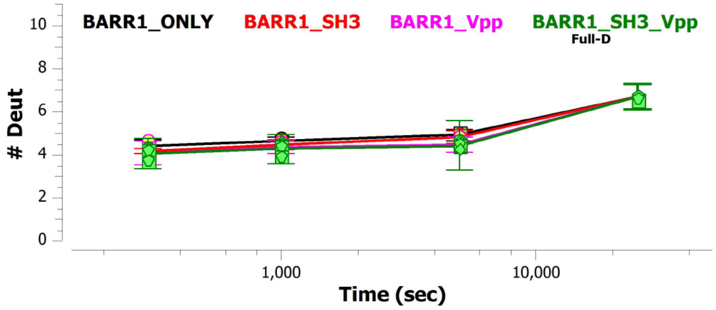

BARR1-393_MINCYS 162-174: LEEKIHKRNSVRL (#76)

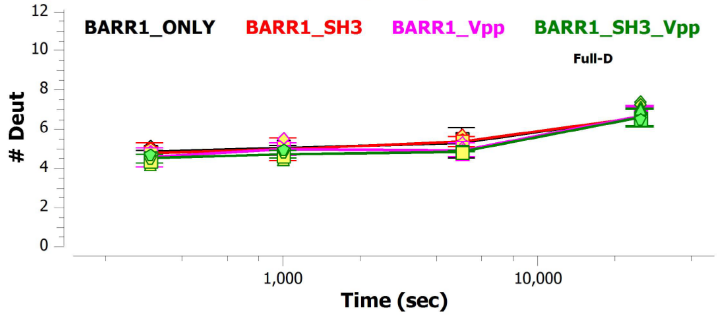

BARR1-393_MINCYS 163-173: EEKIHKRNSVR (#77)

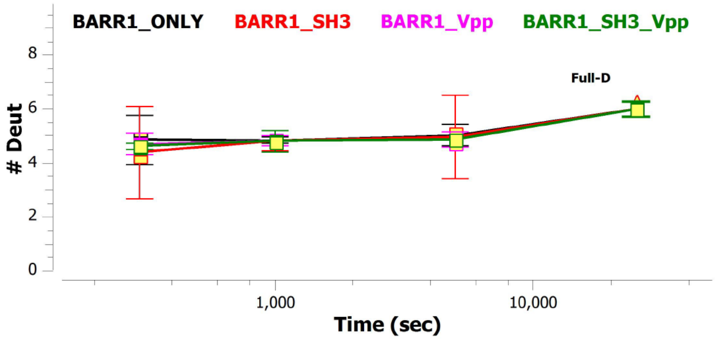

BARR1-393_MINCYS 163-194: EEKIHKRNSVRLVIRKVQYAPERPGPQPTAET (#78)

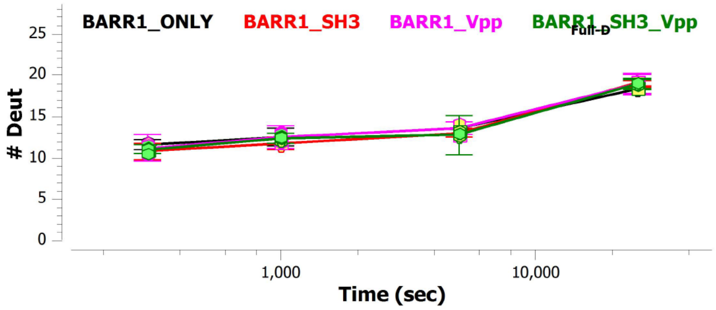

BARR1-393_MINCYS 171-191: SVRLVIRKVQYAPERPGPQPT (#79)

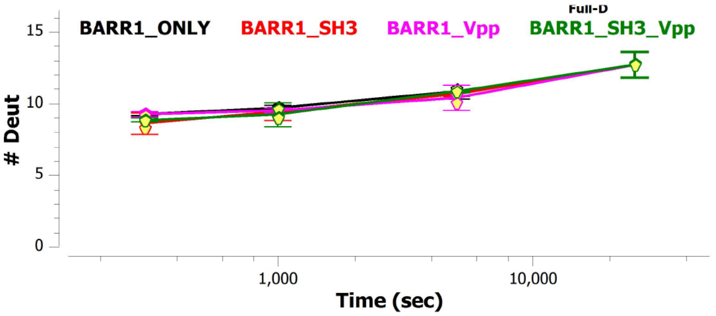

BARR1-393_MINCYS 195-199: TRQFL (#80)

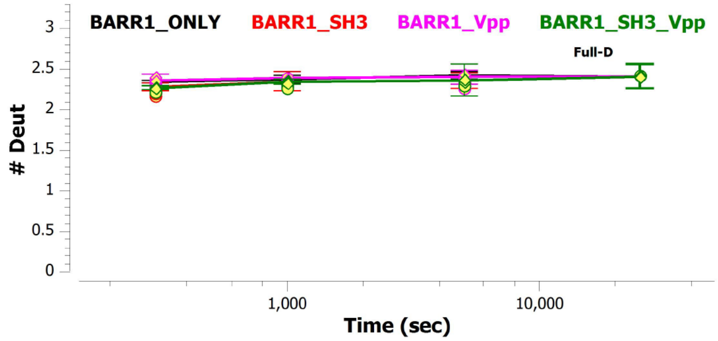

BARR1-393_MINCYS 198-207: FLMSDKPLHL (#81)

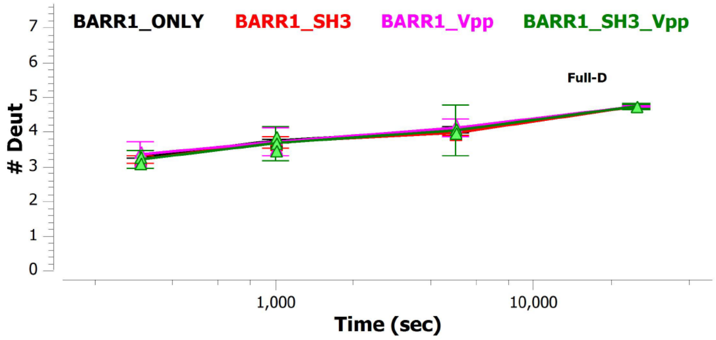

BARR1-393_MINCYS 198-209: FLMSDKPLHLEA (#82)

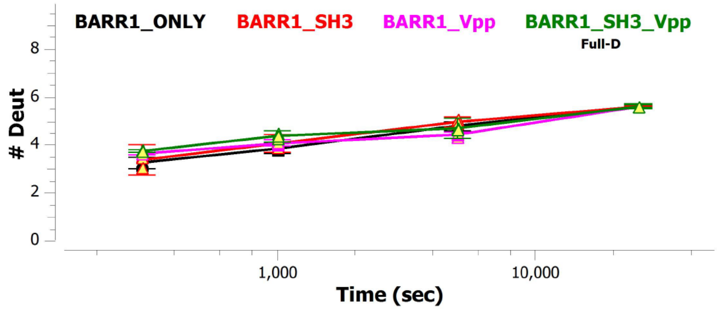

BARR1-393_MINCYS 198-211: FLMSDKPLHLEASL (#83)

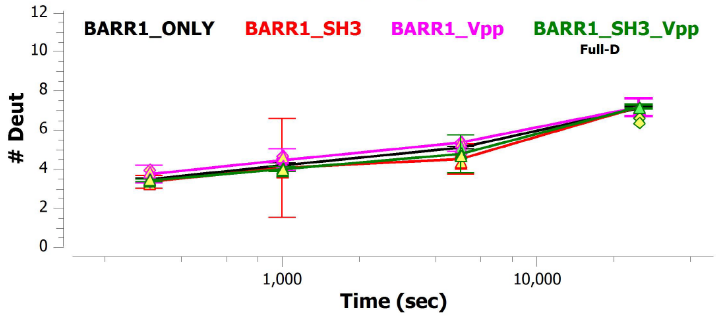

BARR1-393_MINCYS 199-206: LMSDKPLH (#84)

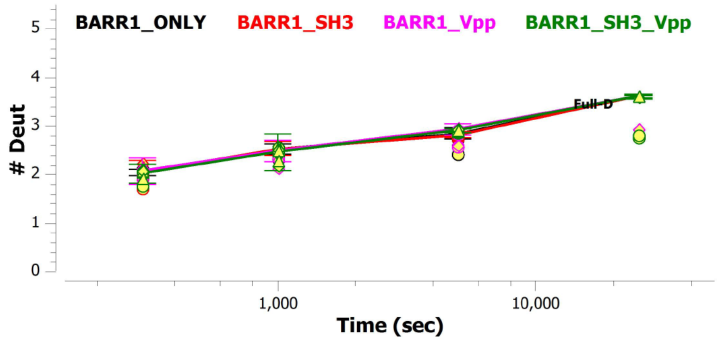

BARR1-393_MINCYS 200-209: MSDKPLHLEA (#85)

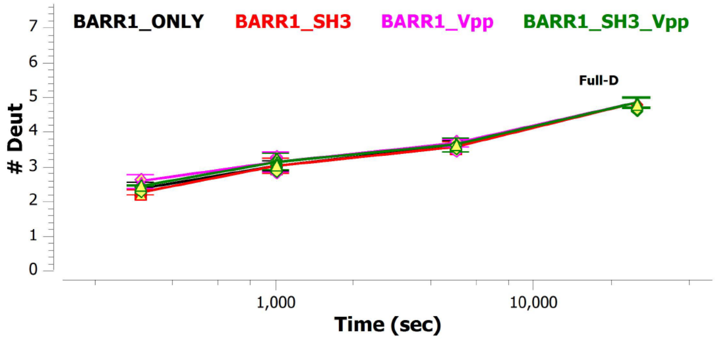

BARR1-393_MINCYS 203-225: KPLHLEASLDKEIYYHGEPISVN (#86)

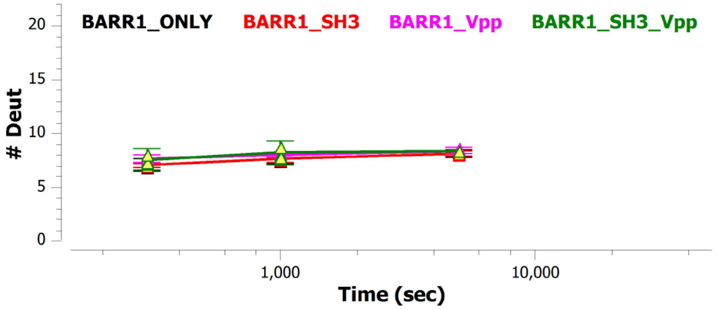

BARR1-393_MINCYS 207-210: LEAS (#87)

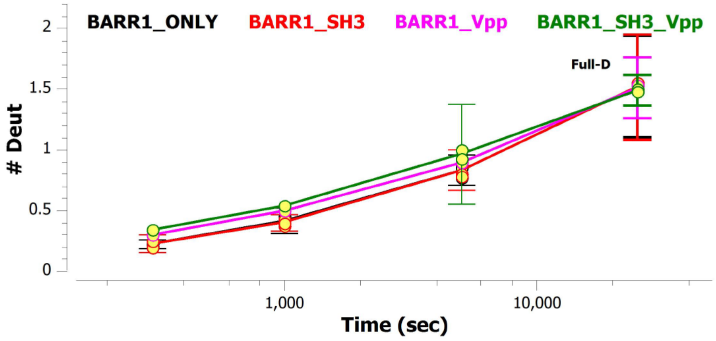

BARR1-393_MINCYS 208-224: EASLDKEIYYHGEPISV (#88)

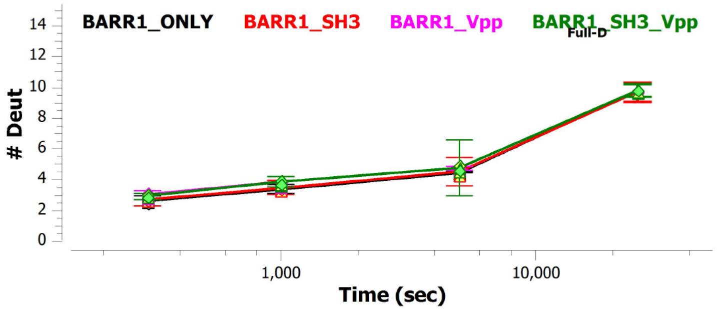

BARR1-393_MINCYS 212-223: DKEIYYHGEPIS (#89)

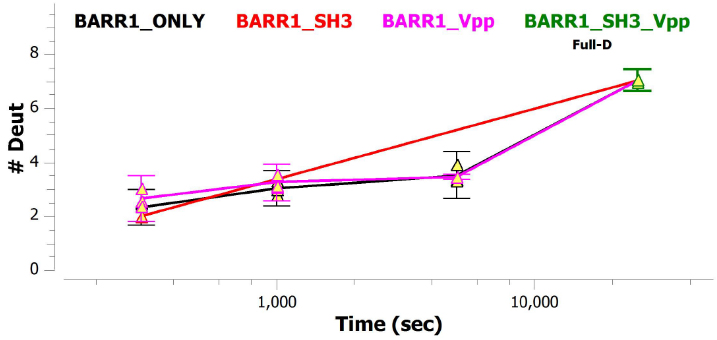

BARR1-393_MINCYS 212-224: DKEIYYHGEPISV (#90)

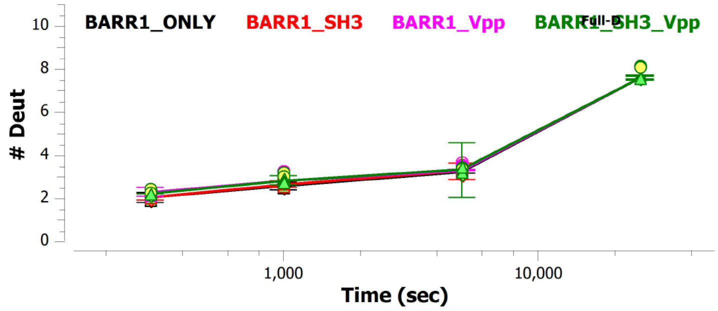

BARR1-393_MINCYS 212-226: DKEIYYHGEPISVNV (#91)

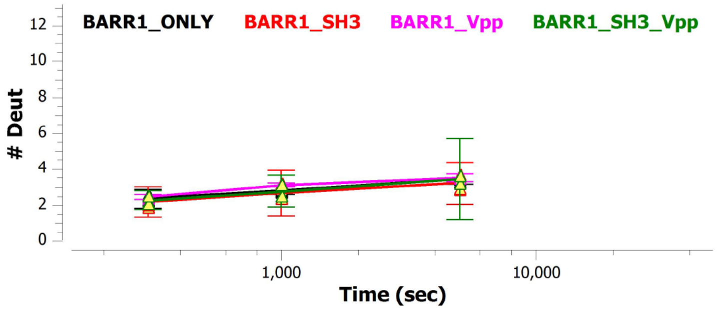

BARR1-393_MINCYS 218-224: HGEPISV (#92)

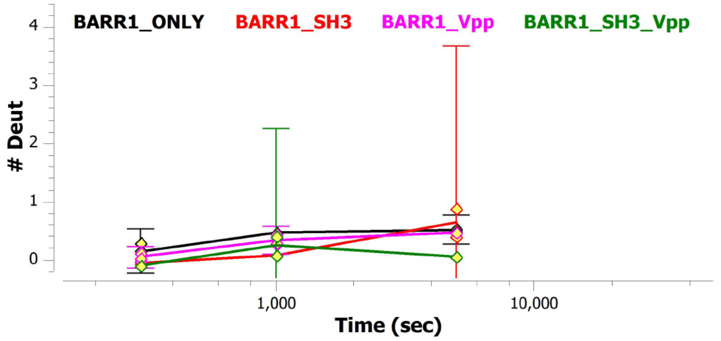

BARR1-393_MINCYS 220-225: EPISVN (#93)

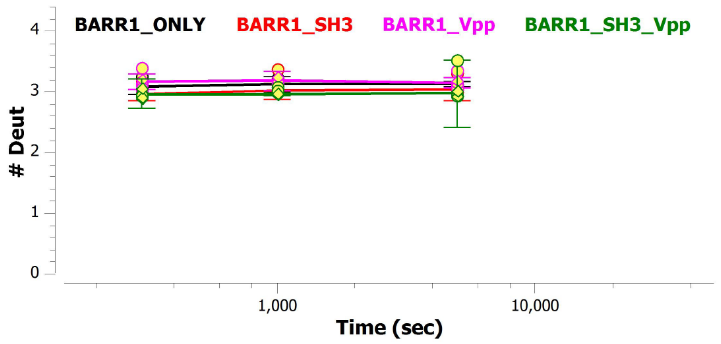

BARR1-393_MINCYS 223-234: SVNVHVTNNTNK (#94)

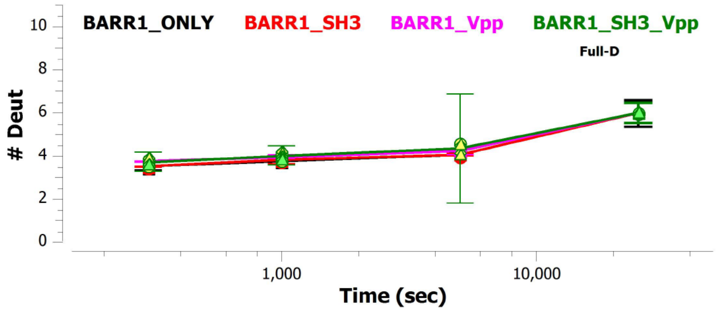

BARR1-393_MINCYS 224-235: VNVHVTNNTNKT (#95)

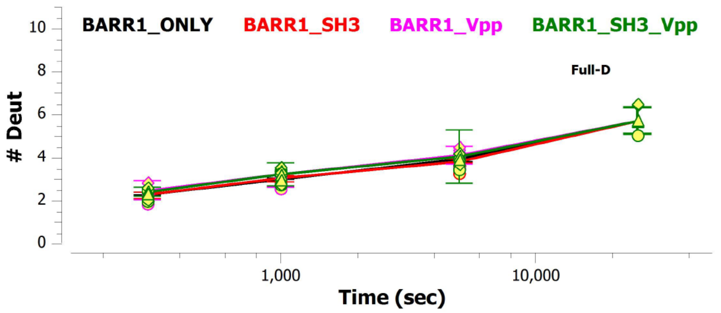

BARR1-393_MINCYS 224-236: VNVHVTNNTNKTV (#96)

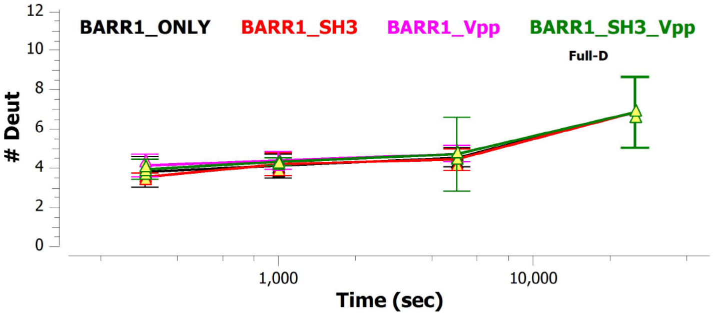

BARR1-393_MINCYS 225-234: NVHVTNNTNK (#97)

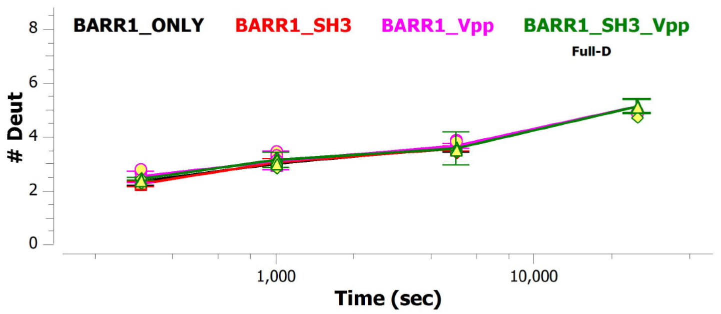

BARR1-393_MINCYS 225-248: NVHVTNNTNKTVKKIKISVRQYAD (#98)

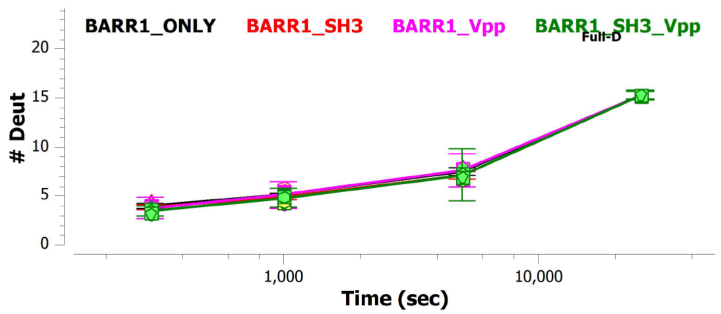

BARR1-393_MINCYS 241-246: ISVRQY (#99)

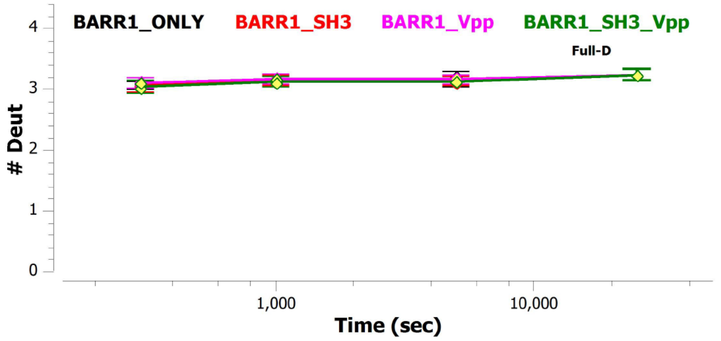

BARR1-393_MINCYS 252-262: FNTAQYKVPVA (#100)

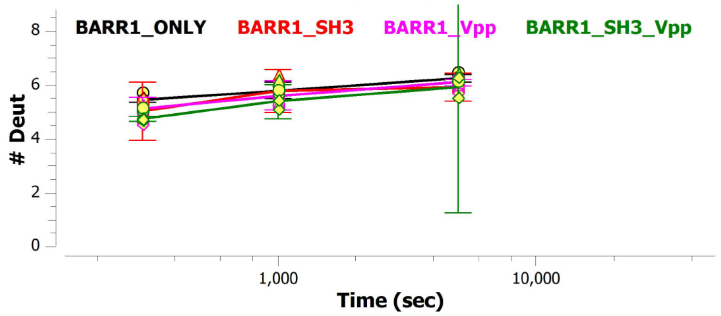

BARR1-393_MINCYS 252-263: FNTAQYKVPVAM (#101)

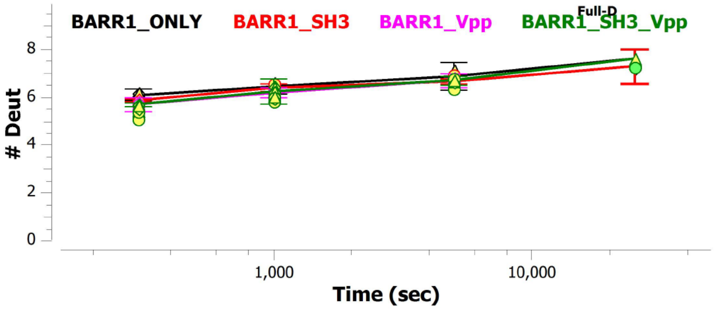

BARR1-393_MINCYS 254-261: TAQYKVPV (#102)

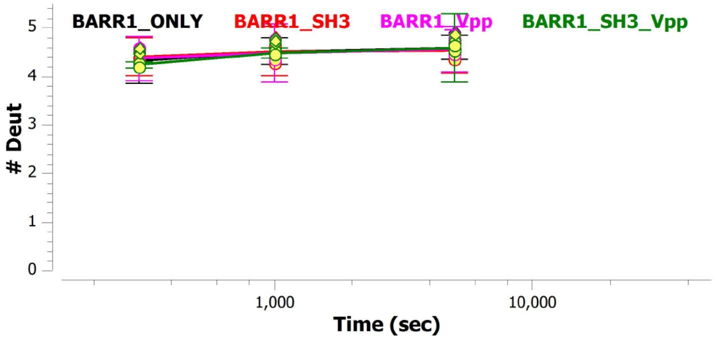

BARR1-393_MINCYS 257-262: YKVPVA (#103)

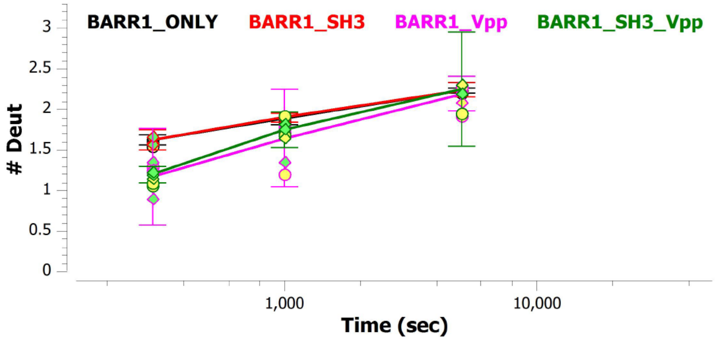

BARR1-393_MINCYS 257-263: YKVPVAM (#104)

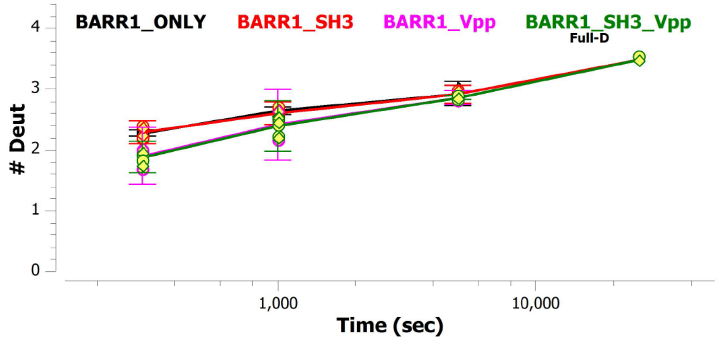

BARR1-393_MINCYS 263-275: MEEADDTVAPSST (#105)

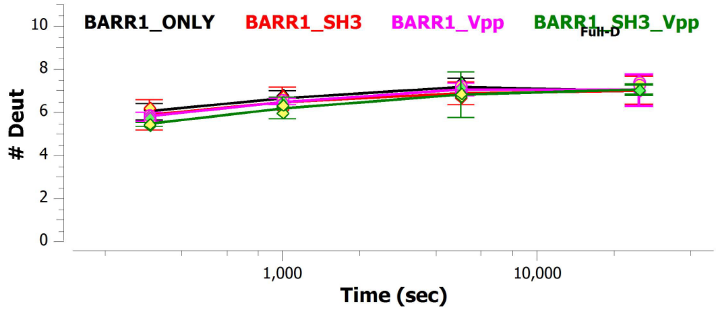

BARR1-393_MINCYS 264-275: EEADDTVAPSST (#106)

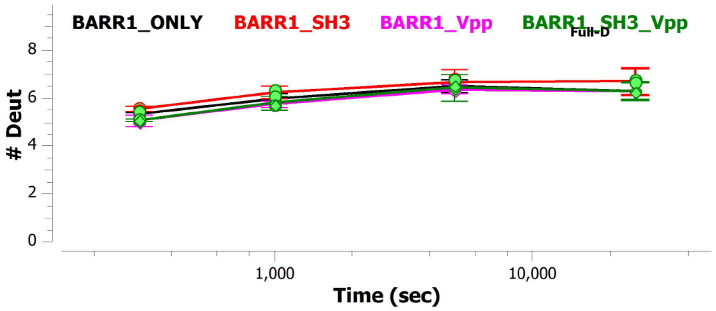

BARR1-393_MINCYS 264-276: EEADDTVAPSSTF (#107)

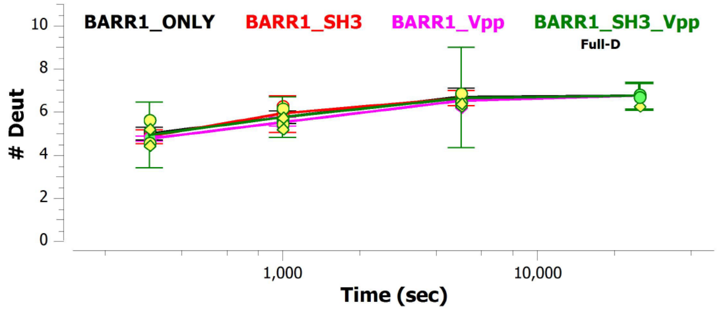

BARR1-393_MINCYS 264-279: EEADDTVAPSSTFSKV (#108)

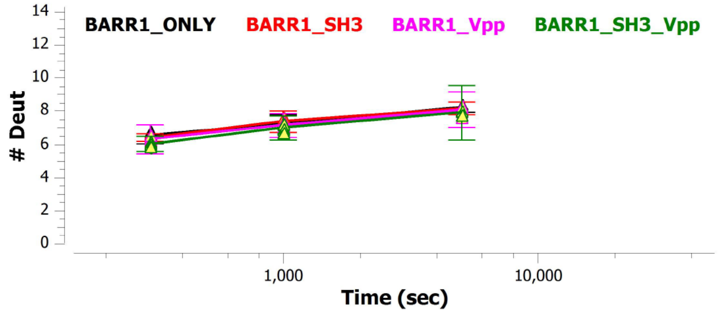

BARR1-393_MINCYS 265-308:
ADDTVAPSSTFSKVYTLTPFLANNREKRGLALDGKLKHEDT[illegible]
(#109)

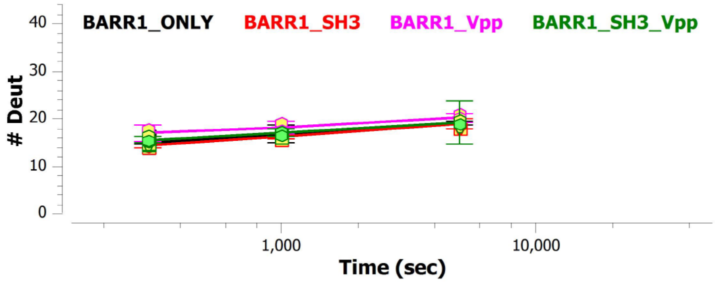

BARR1-393_MINCYS 276-279: FSKV (#110)

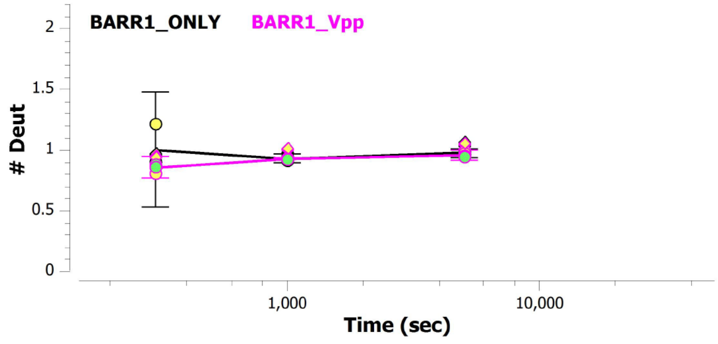

BARR1-393_MINCYS 276-286: FSKVYTLTPFL (#111)

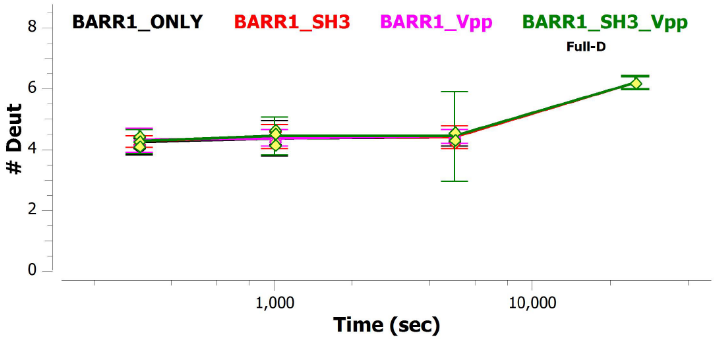

BARR1-393_MINCYS 276-289: FSKVYTLTPFLANN (#112)

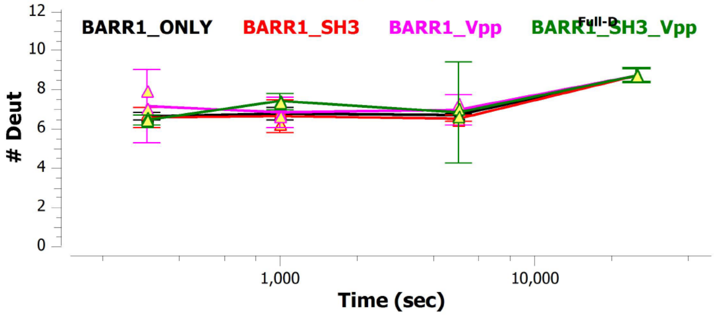

BARR1-393_MINCYS 276-308: FSKVYTLTPFLANNREKRGLALDGKLKHEDTNL (#113)

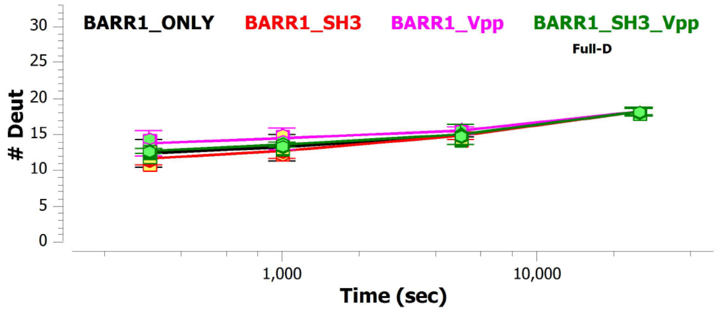

BARR1-393_MINCYS 279-290: VYTLTPFLANNR (#114)

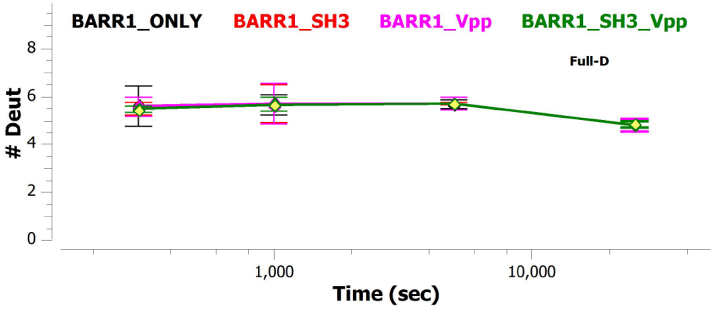

BARR1-393_MINCYS 280-286: YTLTPFL (#115)

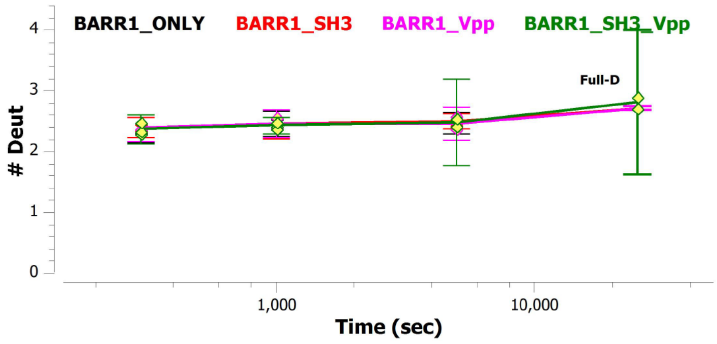

BARR1-393_MINCYS 280-289: YTLTPFLANN (#116)

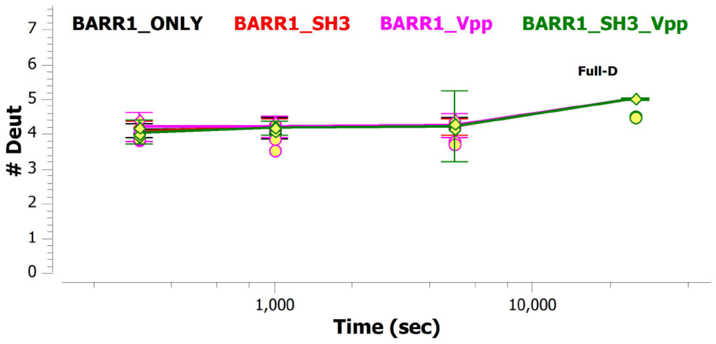

BARR1-393_MINCYS 280-297: YTLTPFLANNREKRGLAL (#117)

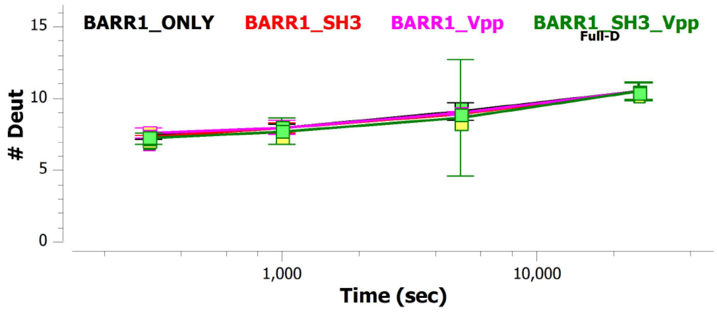

BARR1-393_MINCYS 286-300: LANNREKRGLALDGK (#118)

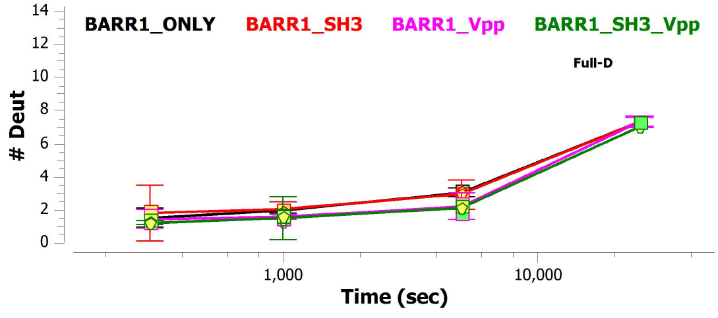

BARR1-393_MINCYS 289-297: NREKRGLAL (#119)

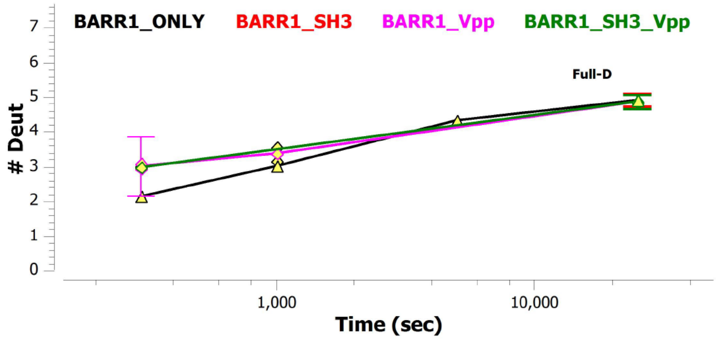

BARR1-393_MINCYS 290-308: REKRGLALDGKLKHEDTNL (#120)

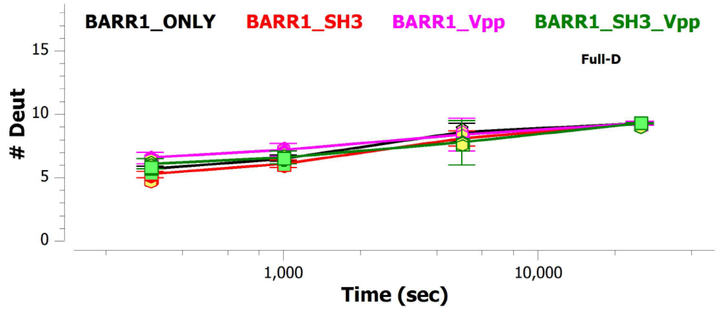

BARR1-393_MINCYS 293-318: RGLALDGKLKHEDTNLASSTLLREGA (#121)

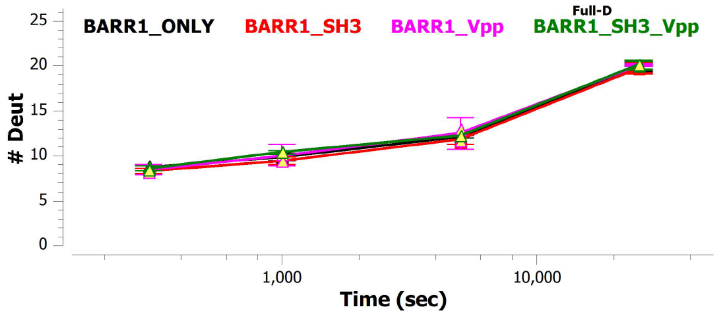

BARR1-393_MINCYS 296-307: ALDGKLKHEDTN (#122)

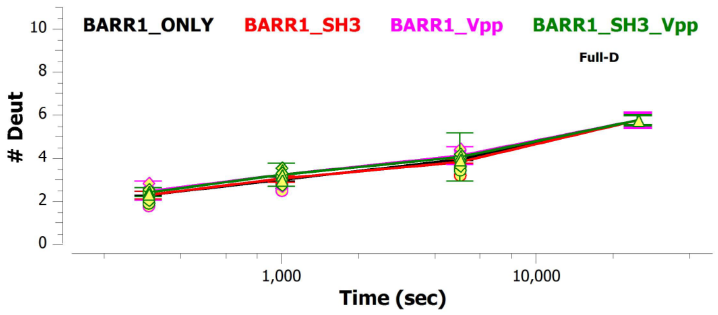

BARR1-393_MINCYS 297-307: LDGKLKHEDTN (#123)

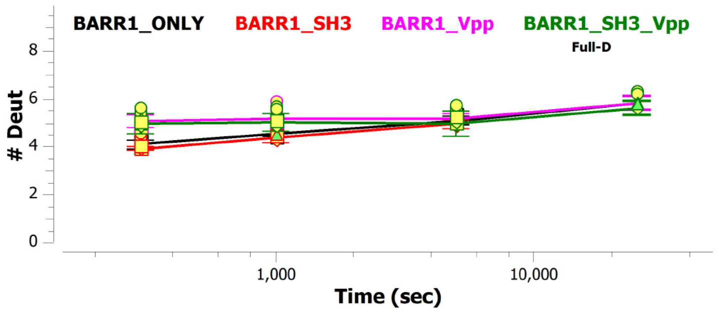

BARR1-393_MINCYS 307-314: NLASSTLL (#124)

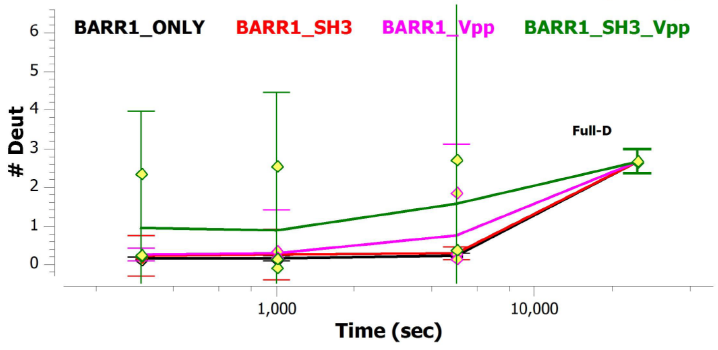

BARR1-393_MINCYS 308-312: LASST (#125)

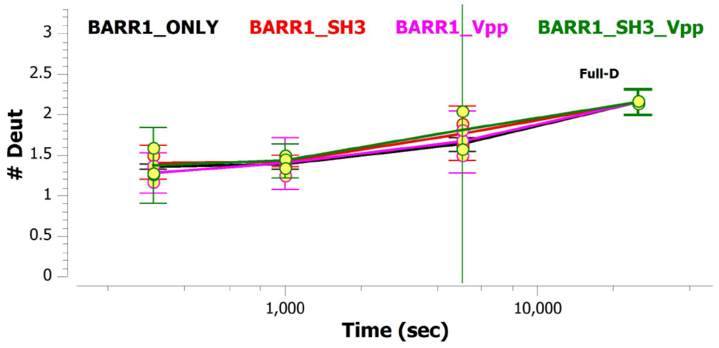

BARR1-393_MINCYS 308-313: LASSTL (#126)

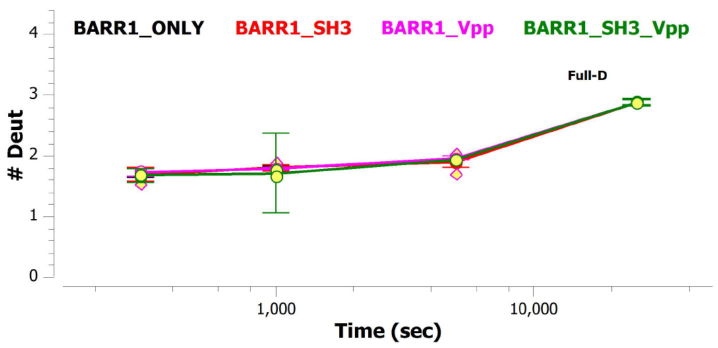

BARR1-393_MINCYS 308-322: LASSTLLREGANREI (#127)

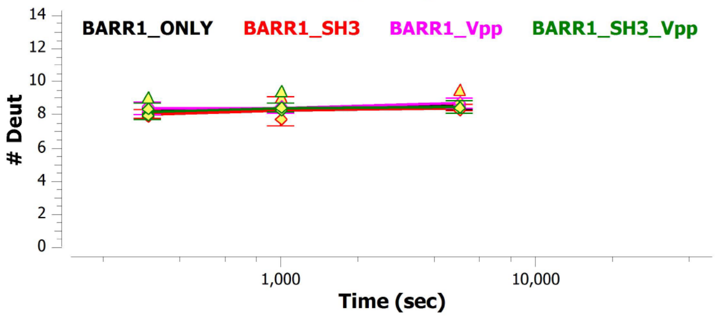

BARR1-393_MINCYS 309-322: ASSTLLREGANREI (#128)

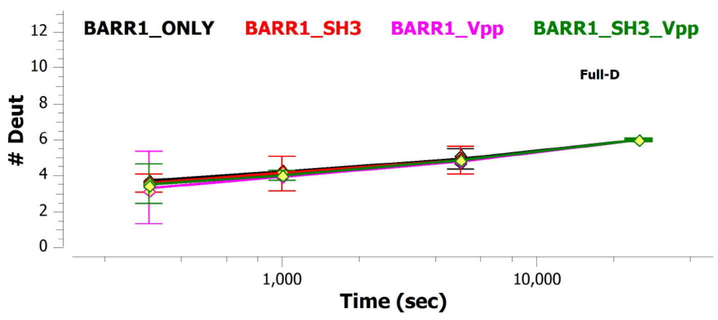

BARR1-393_MINCYS 310-315: SSTLLR (#129)

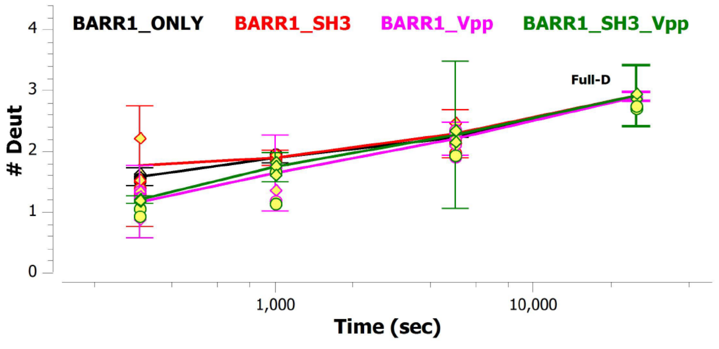

BARR1-393_MINCYS 313-317: LLREG (#130)

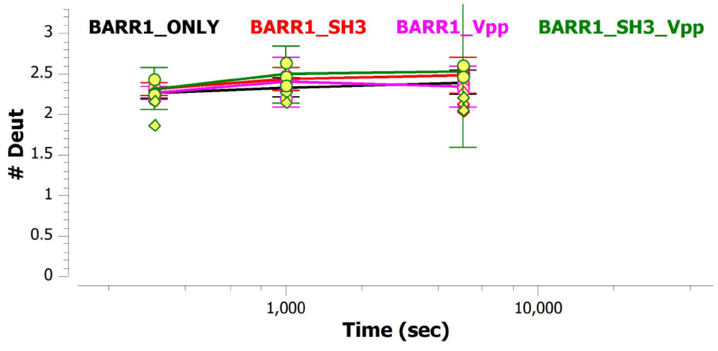

BARR1-393_MINCYS 313-322: LLREGANREI (#131)

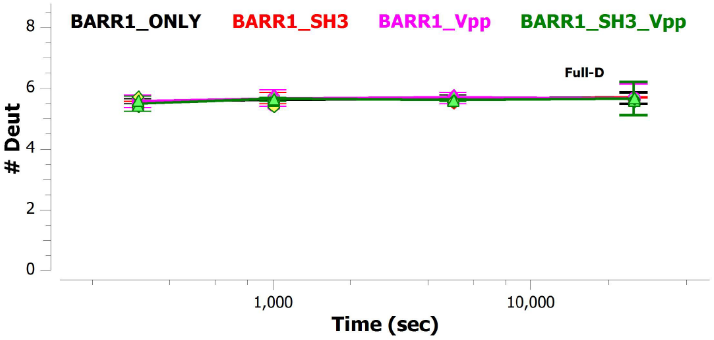

BARR1-393_MINCYS 314-322: LREGANREI (#132)

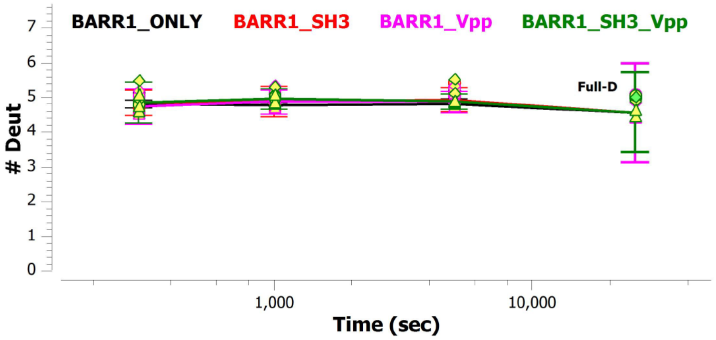

BARR1-393_MINCYS 314-324: LREGANREILG (#133)

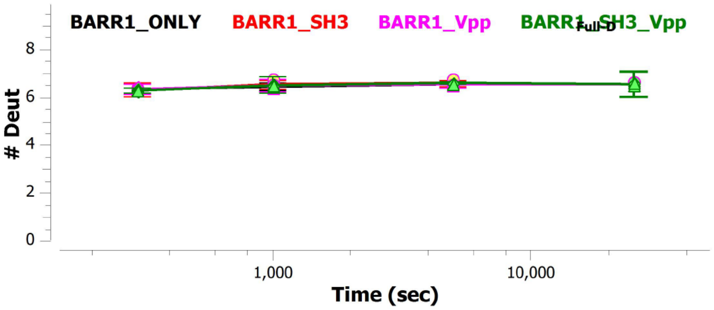

BARR1-393_MINCYS 315-324: REGANREILG (#134)

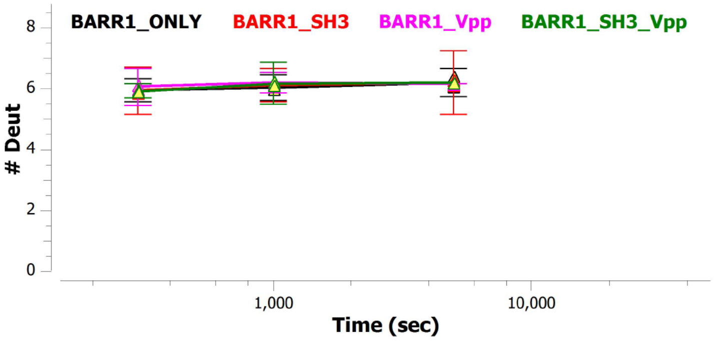

BARR1-393_MINCYS 320-323: REIL (#135)

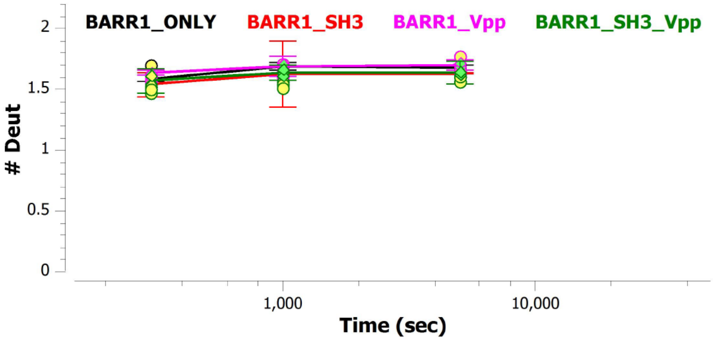

BARR1-393_MINCYS 320-326: REILGII (#136)

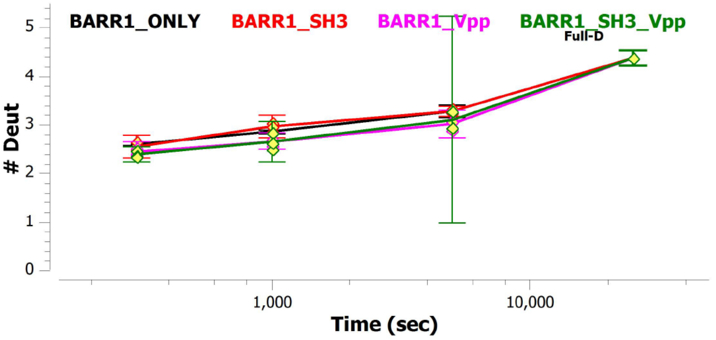

BARR1-393_MINCYS 320-328: REILGIIVS (#137)

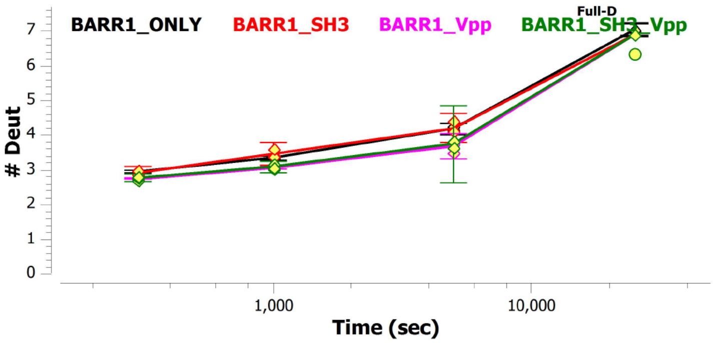

BARR1-393_MINCYS 320-329: REILGIIVSY (#138)

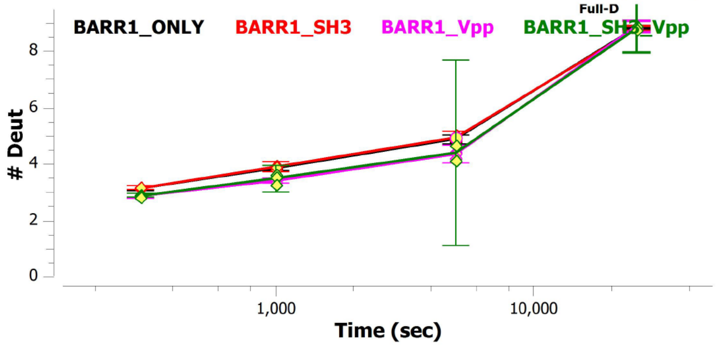

BARR1-393_MINCYS 321-325: EILGI (#139)

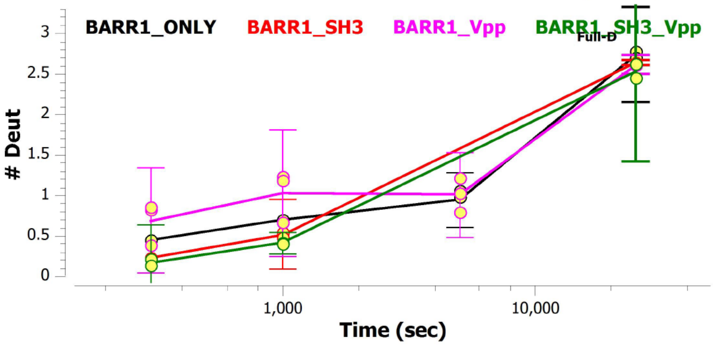

BARR1-393_MINCYS 324-328: GIIVS (#140)

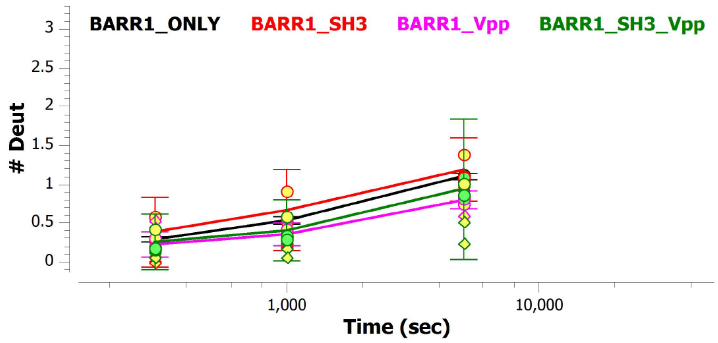

BARR1-393_MINCYS 324-329: GIIVSY (#141)

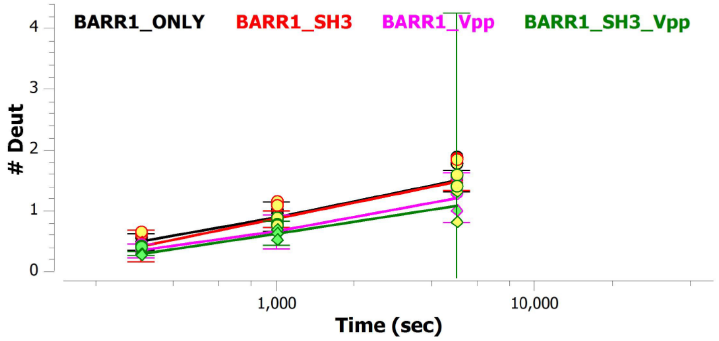

BARR1-393_MINCYS 324-335: GIIVSYKVKVKL (#142)

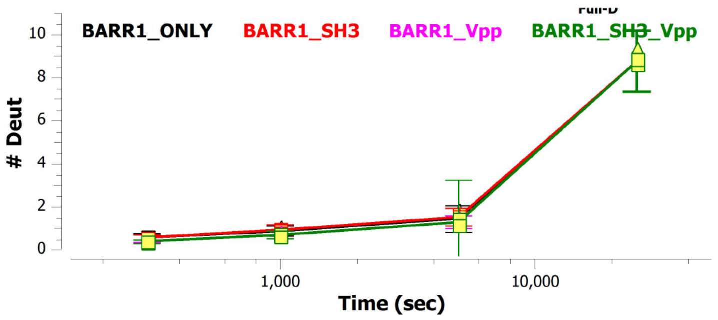

BARR1-393_MINCYS 325-328: IIVS (#143)

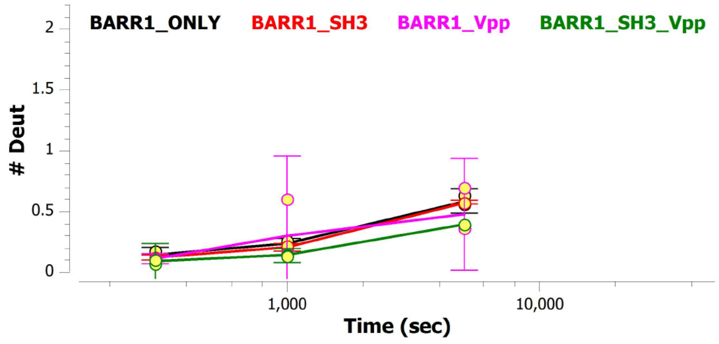

BARR1-393_MINCYS 325-329: IIVSY (#144)

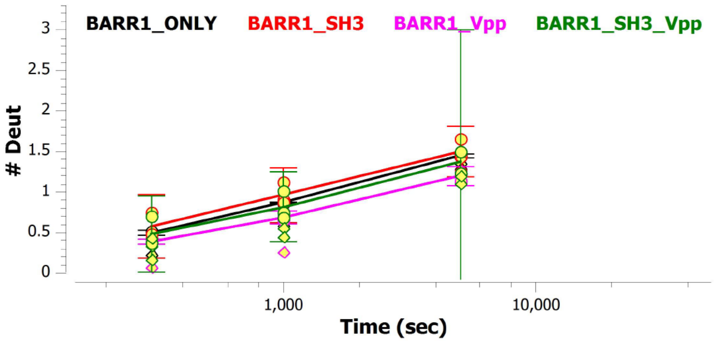

BARR1-393_MINCYS 329-335: YKVKVKL (#145)

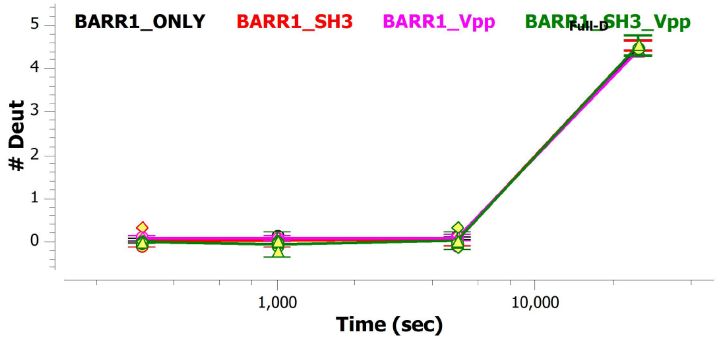

BARR1-393_MINCYS 330-335: KVKVKL (#146)

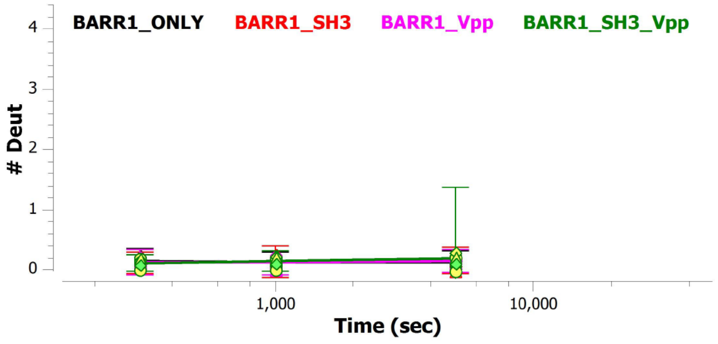

BARR1-393_MINCYS 330-346: KVKVKLVVSRGGLLGDL (#147)

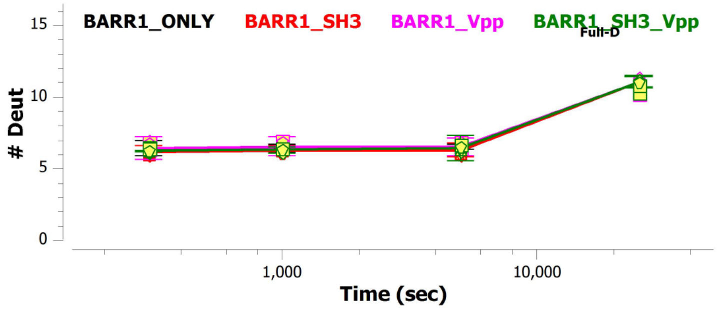

BARR1-393_MINCYS 335-341: LVVSRGG (#148)

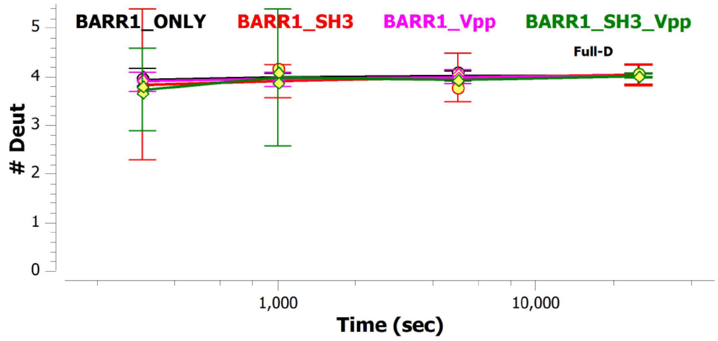

BARR1-393_MINCYS 335-342: LVVSRGGL (#149)

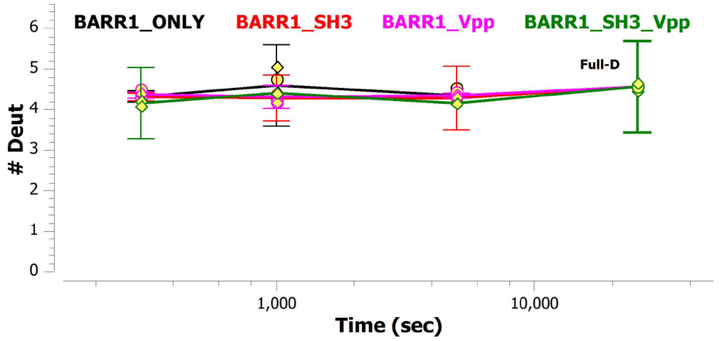

BARR1-393_MINCYS 336-346: VVSRGGLLGDL (#150)

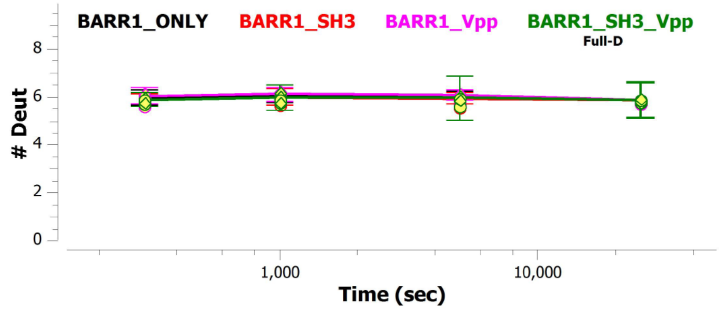

BARR1-393_MINCYS 336-351: VVSRGGLLGDLASSDV (#151)

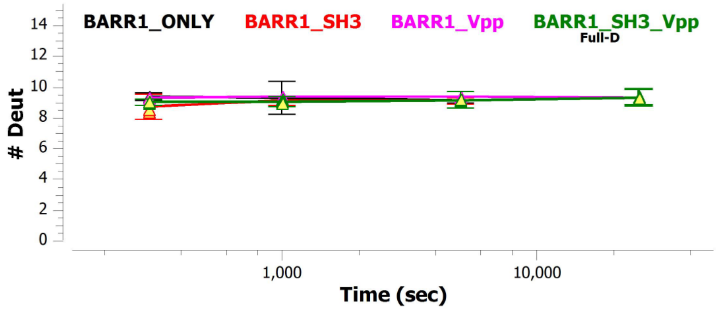

BARR1-393_MINCYS 336-352: VVSRGGLLGDLASSDVA (#152)

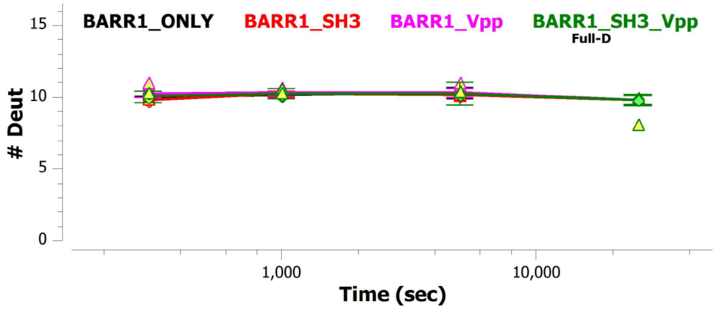

BARR1-393_MINCYS 337-351: VSRGGLLGDLASSDV (#153)

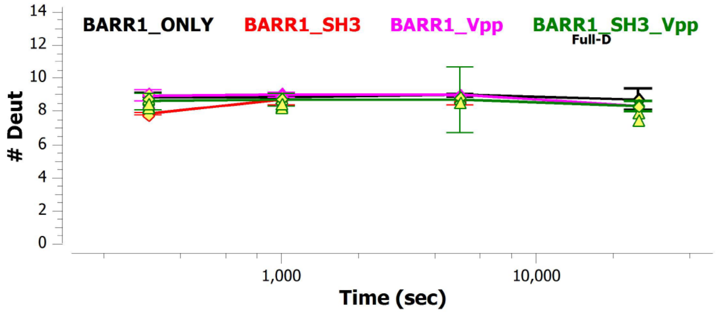

BARR1-393_MINCYS 344-352: GDLASSDVA (#154)

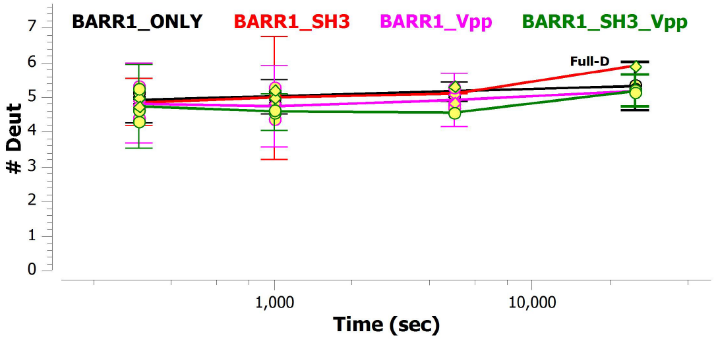

BARR1-393_MINCYS 346-383: LASSDVAVELPFTLMHPKPKEEPPHREVPESETPVDTN (#155)

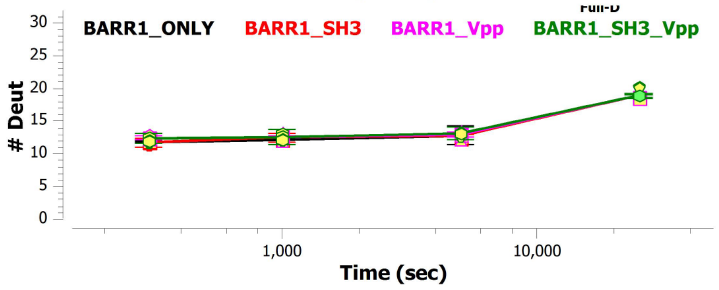

BARR1-393_MINCYS 351-384: VAVELPFTLMHPKPKEEPPHREVPESETPVDTNL (#156)

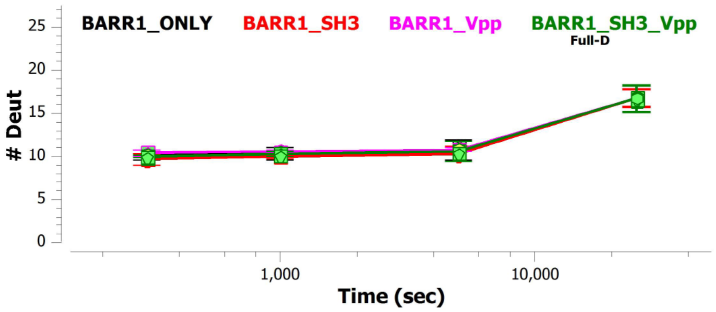

BARR1-393_MINCYS 353-378: VELPFTLMHPKPKEEPPHREVPESET (#157)

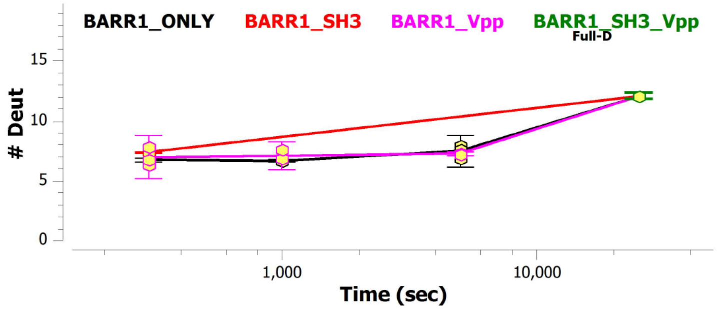

BARR1-393_MINCYS 356-384: PFTLMHPKPKEEPPHREVPESETPVDTNL (#158)

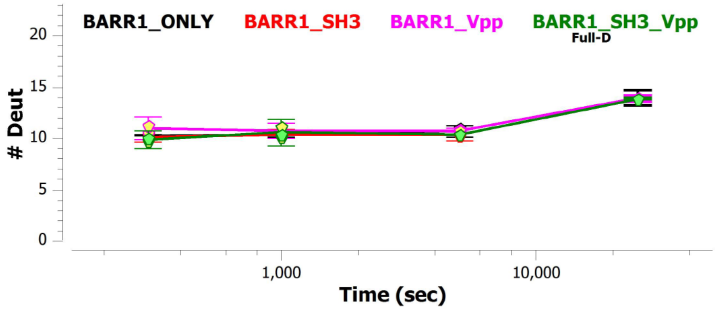

BARR1-393_MINCYS 360-385: MHPKPKEEPPHREVPESETPVDTNLI (#159)

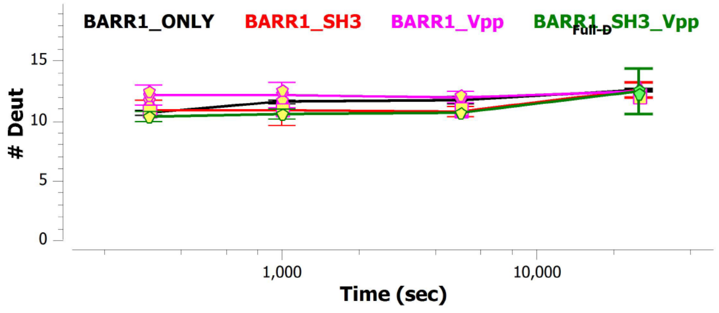

BARR1-393_MINCYS 361-384: HPKPKEEPPHREVPESETPVDTNL (#160)

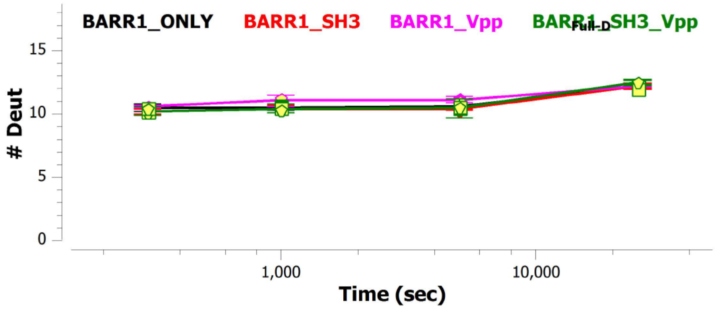

BARR1-393_MINCYS 385-396: IELDTNDDDIVF (#161)

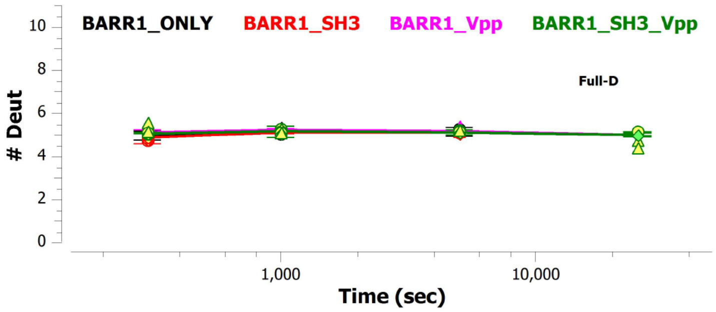

BARR1-393_MINCYS 386-394: ELDTNDDDI (#162)

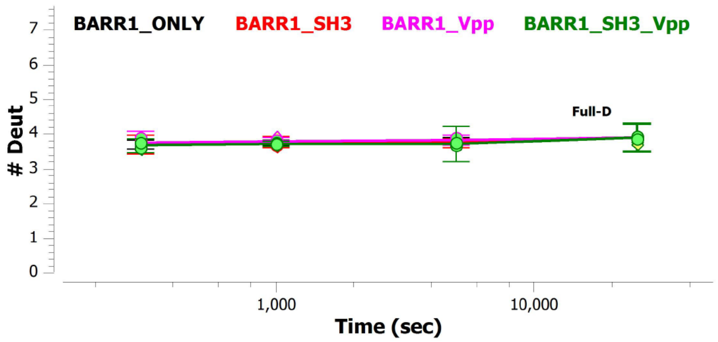

BARR1-393_MINCYS 388-396: DTNDDDIVF (#163)

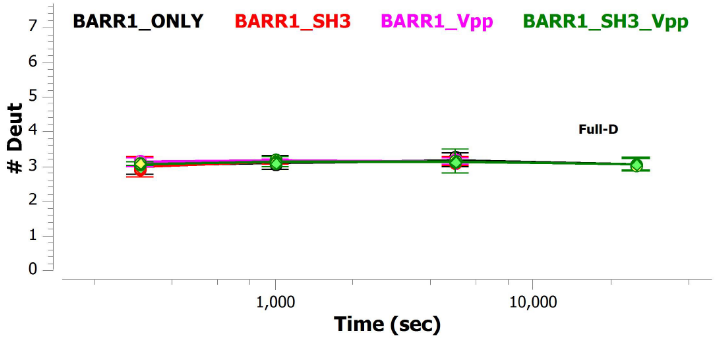

BARR1-393_MINCYS 391-399: DDDIVFEDF (#164)

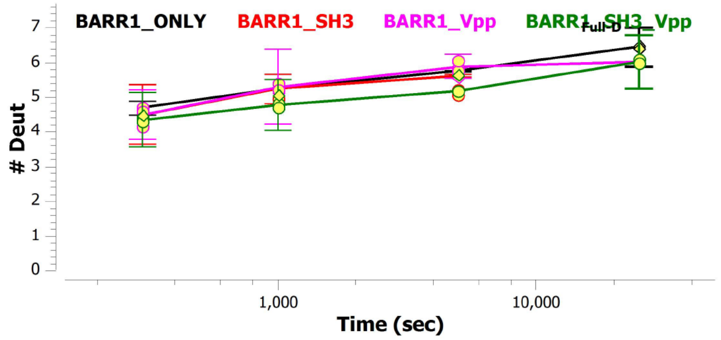

BARR1-393_MINCYS 397-401: EDFAR (#165)

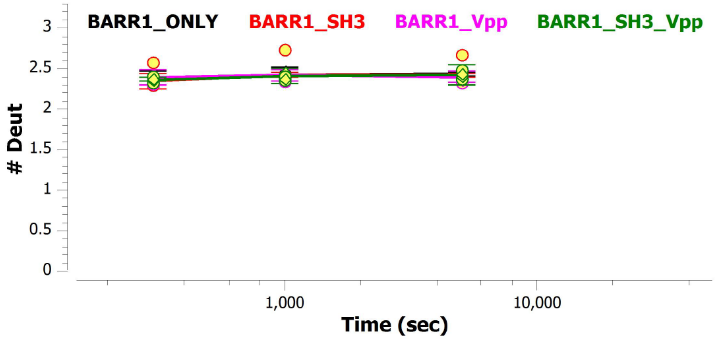

Supplement: Supplementary file 7 — Supplementary Data 5 [file 41467_2026_69884_MOESM7_ESM.pdf]
