# Supplementary material for: Mechanism of beta-arrestin 1 mediated Src activation via Src SH3 domain revealed by cryo-electron microscopy
Source: Nat Commun. 2026 Feb 20;17:2973. doi: 10.1038/s41467-026-69884-1 (PMC13035853; doi:10.1038/s41467-026-69884-1)

SH3 8-19: HHHDYDIPTTEN (#1)

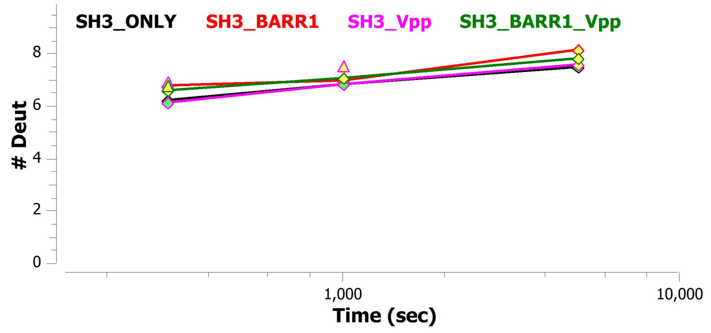

SH3 8-20: HHHDYDIPTTENL (#2)

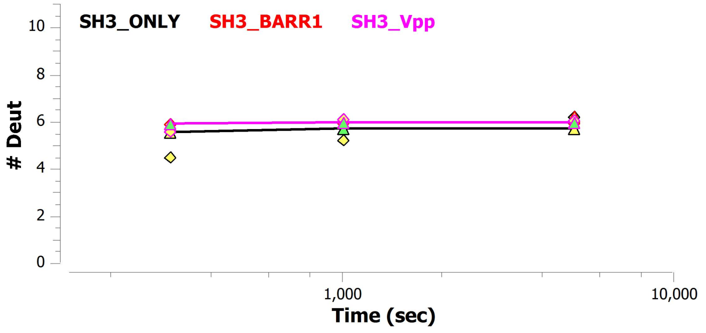

SH3 9-20: HHDYDIPTTENL (#3)

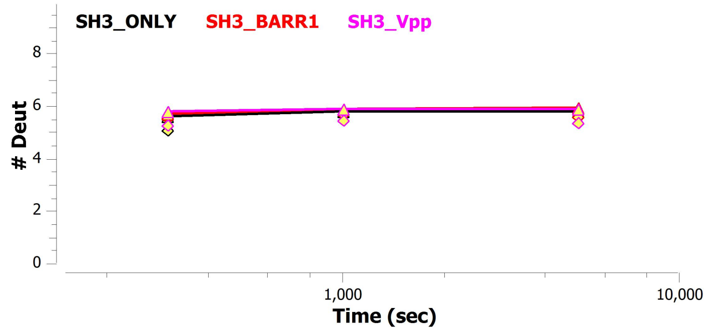

SH3 13-20: DIPTTENL (#4)

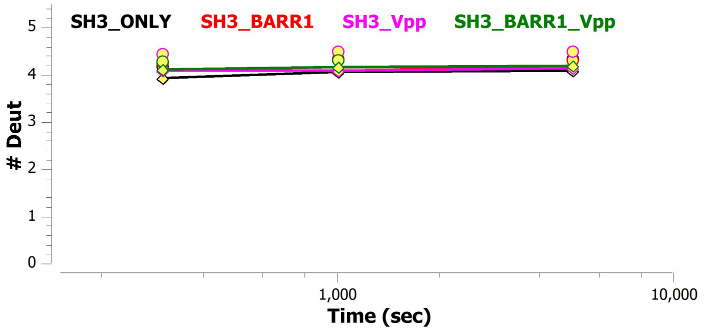

SH3 19-24: NLYFQG (#5)

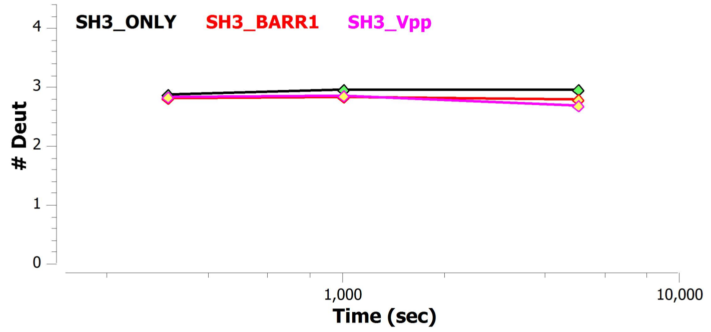

SH3 20-30: LYFQGHMVTTF (#6)

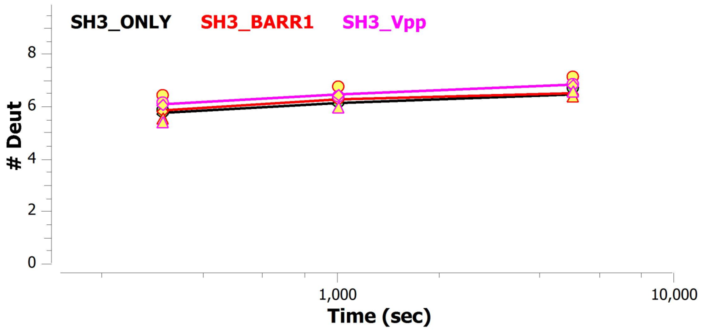

SH3 21-24: YFQG (#7)

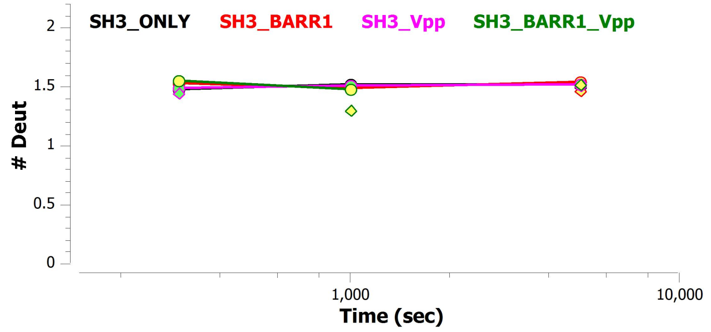

SH3 21-28: YFQGHMVT (#8)

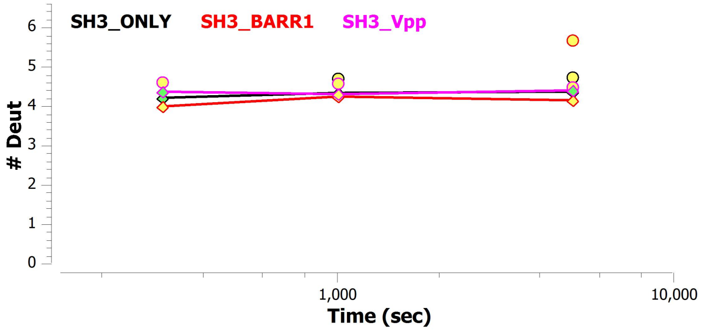

SH3 21-29: YFQGHMVTT (#9)

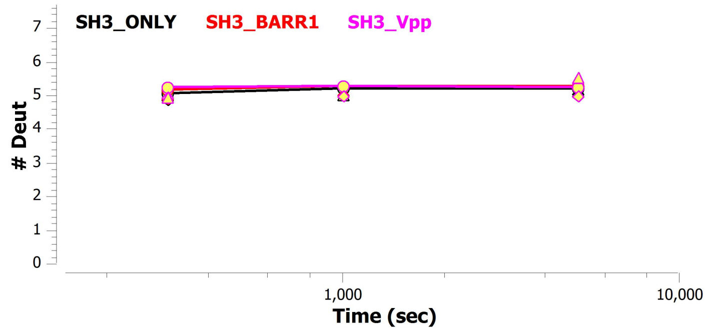

SH3 21-30: YFQGHMVTTF (#10)

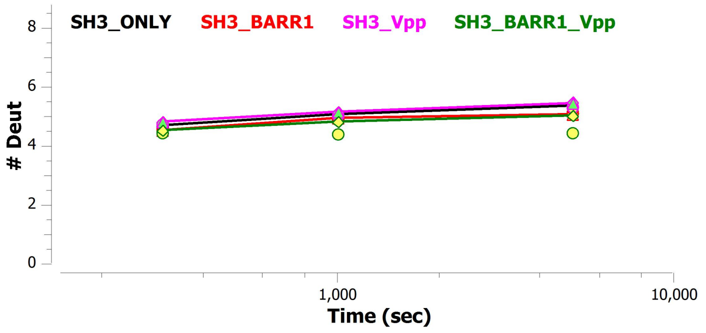

SH3 22-29: FQGHMVTT (#11)

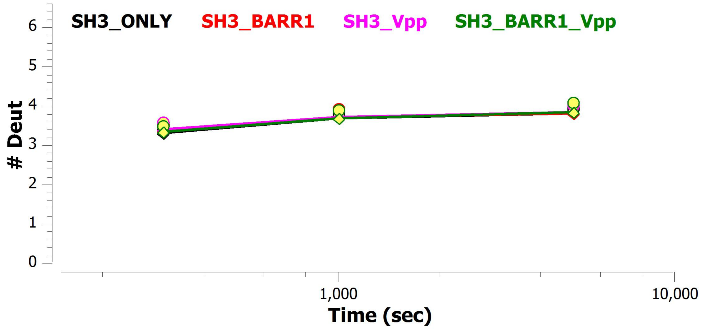

SH3 22-30: FQGHMVTTF (#12)

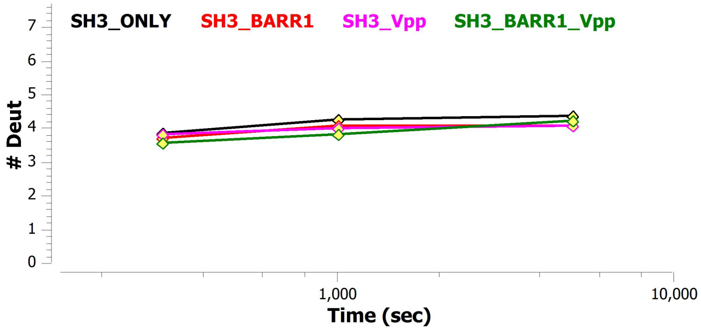

SH3 23-30: QGHMVTTF (#13)

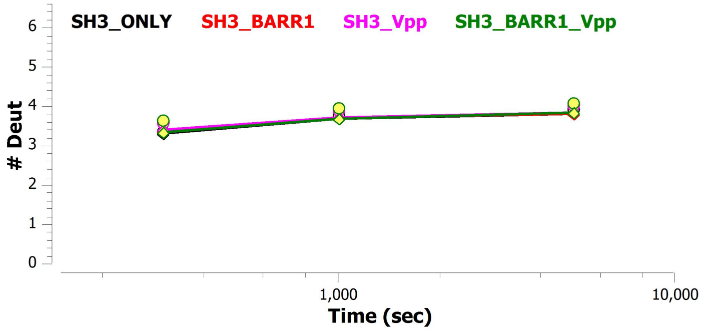

SH3 24-30: GHMVTTF (#14)

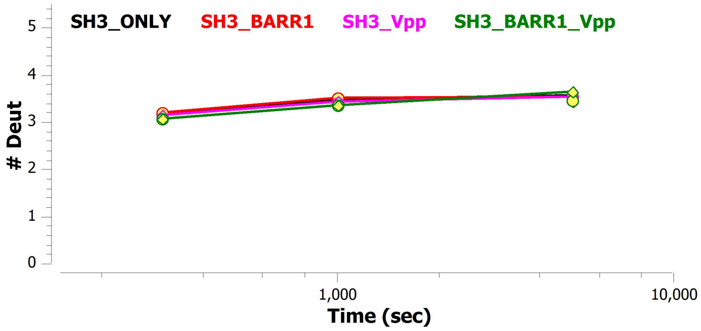

SH3 25-30: HMVTTF (#15)

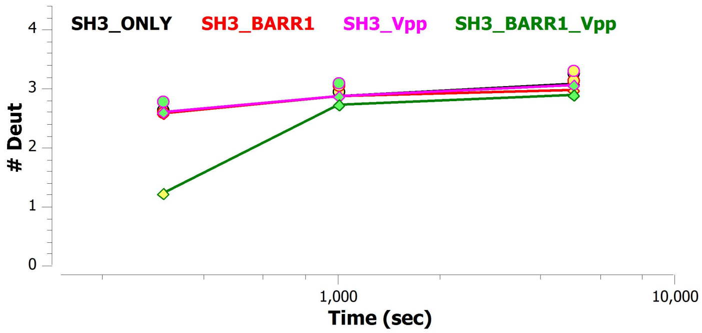

SH3 26-30: MVTTF (#16)

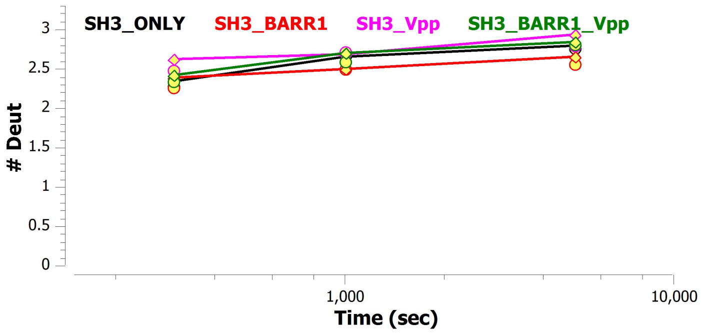

SH3 27-30: VTTF (#17)

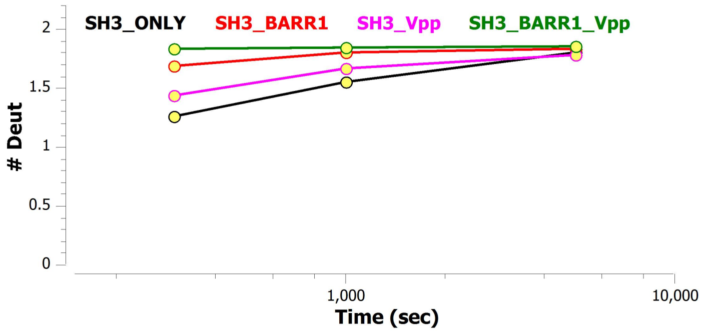

SH3 28-31: TTFV (#18)

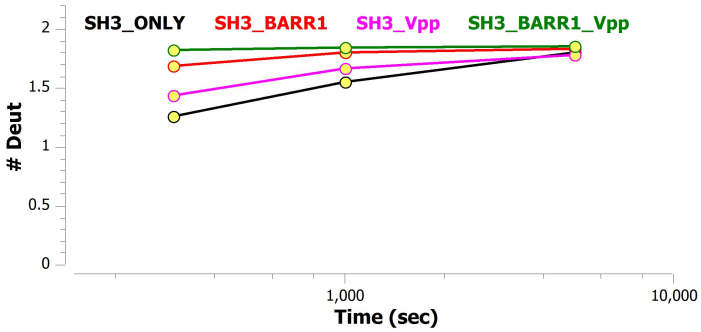

SH3 31-44: VALYDYESRTETDL (#19)

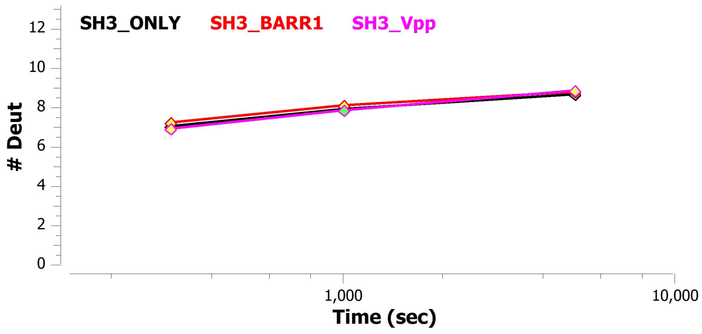

SH3 33-45: LYDYESRTETDLS (#20)

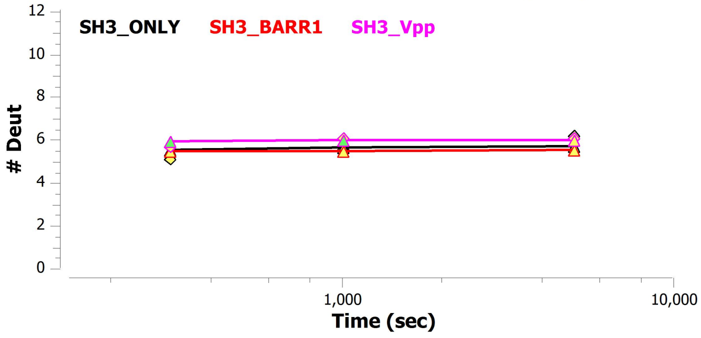

SH3 33-51: LYDYESRTETDLSFKKGER (#21)

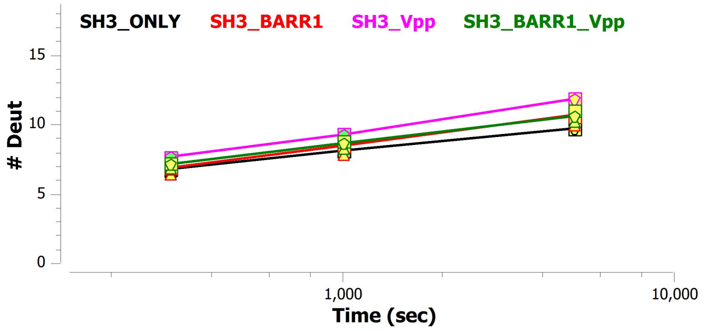

SH3 34-40: YDYESRT (#22)

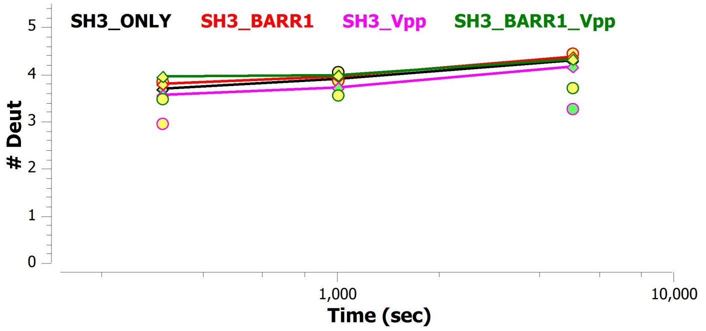

SH3 34-42: YDYESRTET (#23)

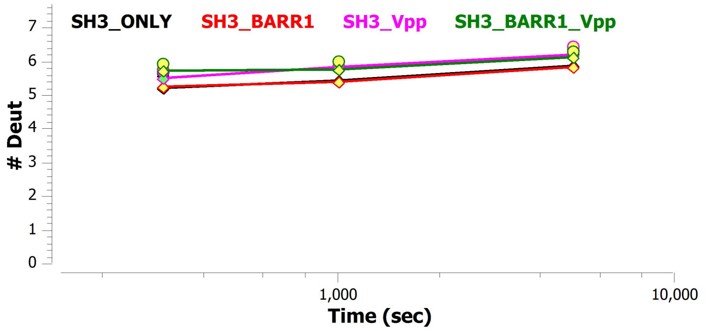

SH3 34-44: YDYESRTETDL (#24)

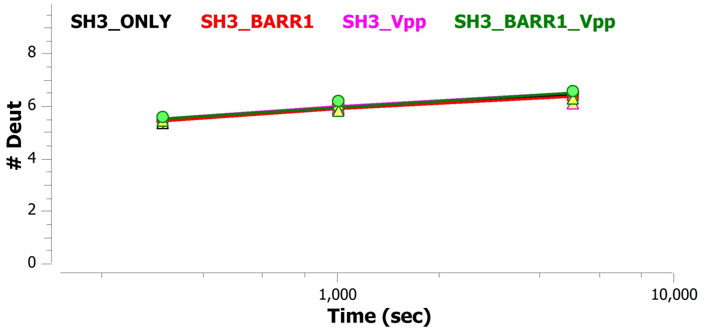

SH3 34-45: YDYESRTETDLS (#25)

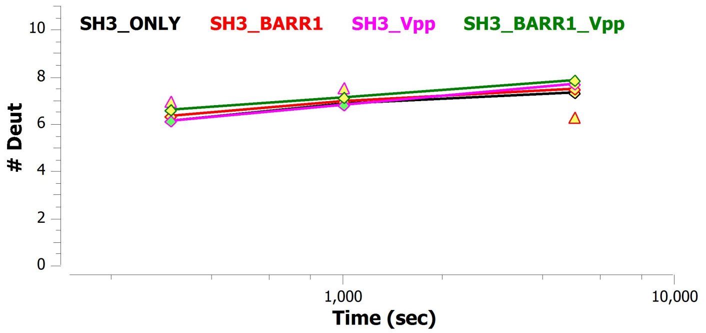

SH3 34-46: YDYESRTETDLSF (#26)

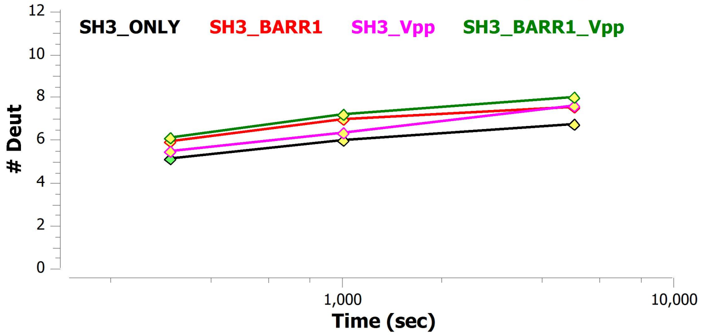

SH3 35-42: DYESRTET (#27)

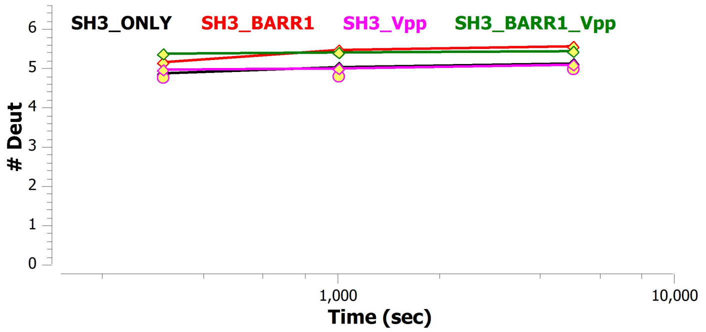

SH3 37-44: ESRTETDL (#28)

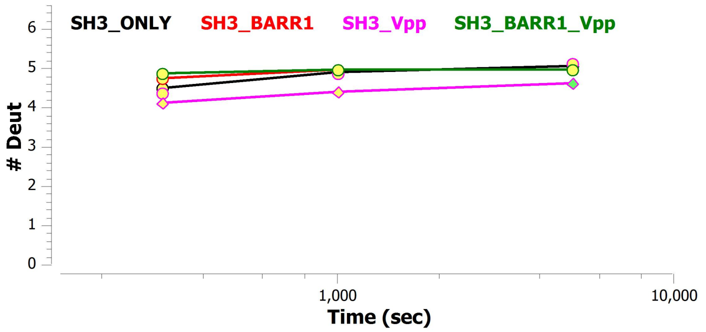

SH3 39-45: RTETDLS (#29)

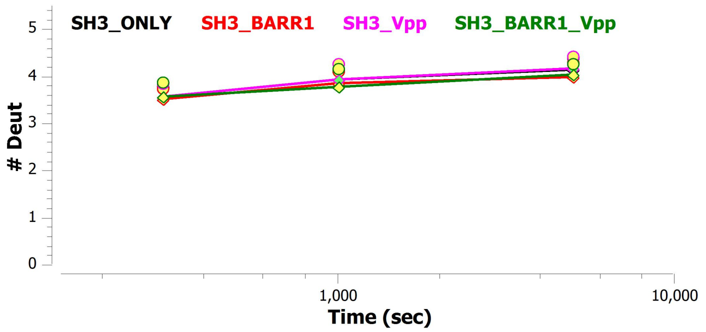

SH3 41-52: ETDLSFKKGERL (#30)

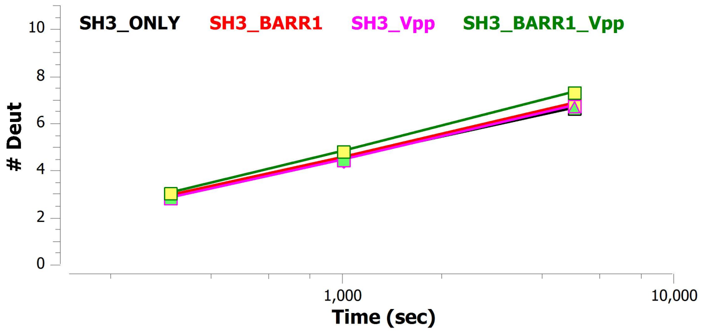

SH3 43-52: DLSFKKGERL (#31)

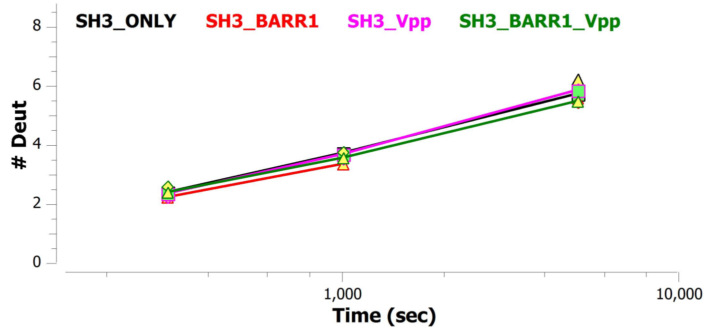

SH3 43-62: DLSFKKGERLQIVNNTEGDW (#32)

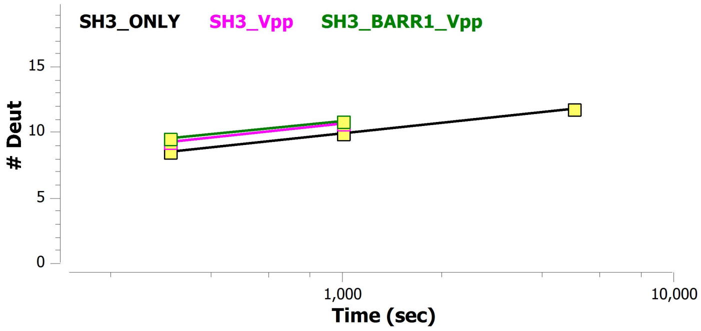

SH3 45-52: SFKKGERL (#33)

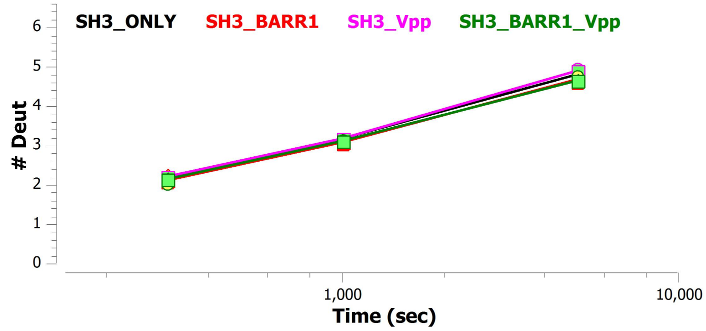

SH3 45-57: SFKKGERLQIVNN (#34)

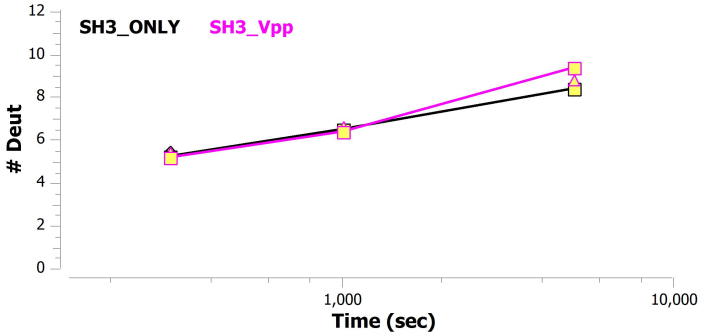

SH3 45-62: SFKKGERLQIVNNTEGDW (#35)

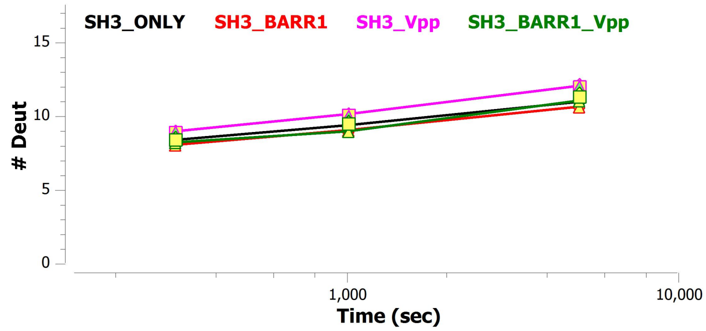

SH3 46-52: FKKGERL (#36)

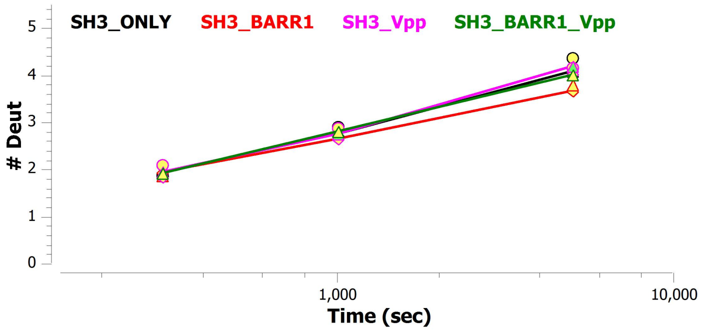

SH3 46-62: FKKGERLQIVNNTEGDW (#37)

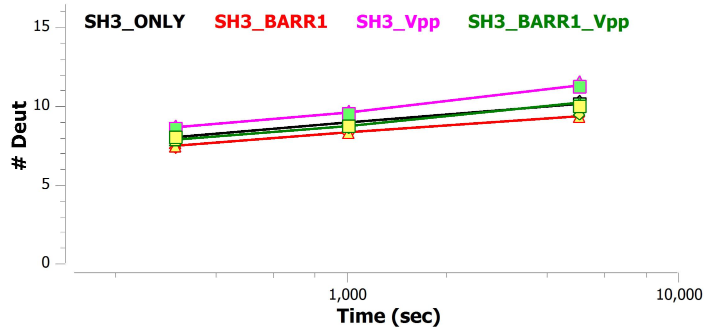

SH3 47-62: KKGERLQIVNNTEGDW (#38)

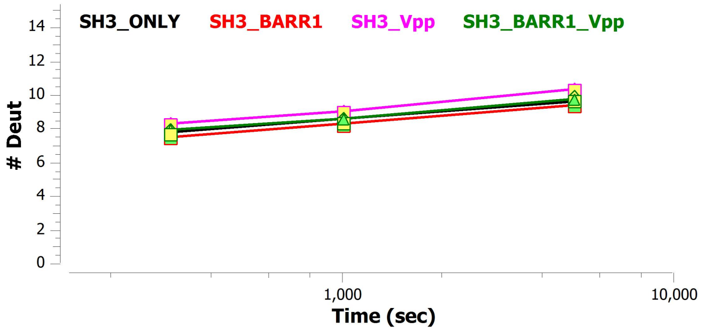

SH3 53-62: QIVNNTEGDW (#39)

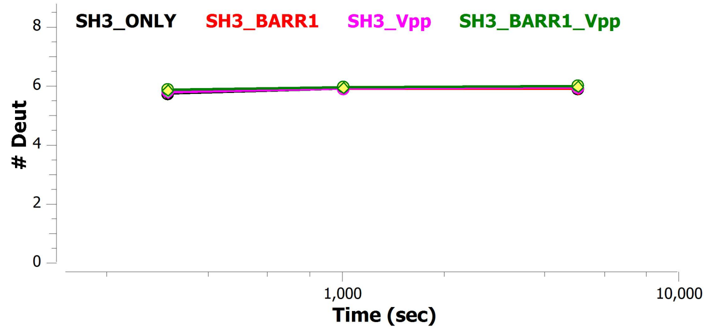

SH3 57-66: NTEGDWWLAH (#40)

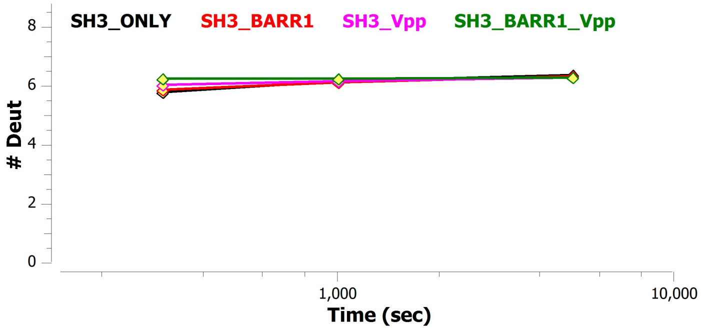

SH3 63-79: WLAHSLTTGQTGYIPSN (#41)

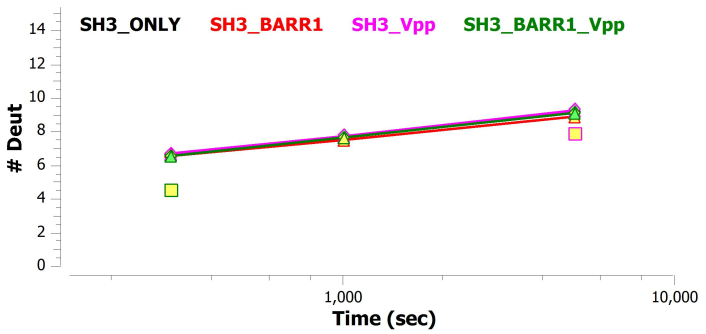

SH3 63-86: WLAHSLTTGQTGYIPSNYVAPSDD (#42)

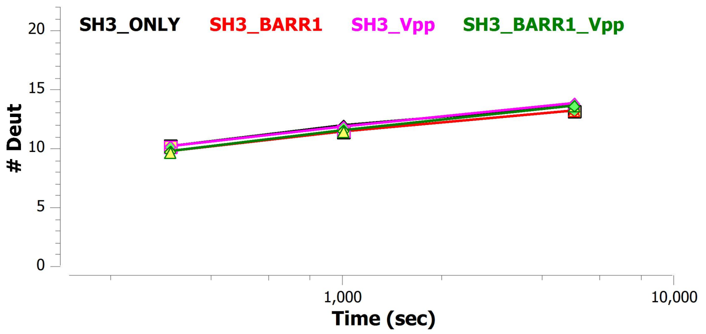

SH3 63-93: WLAHSLTTGQTGYIPSNYVAPSDDYKDDDDK (#43)

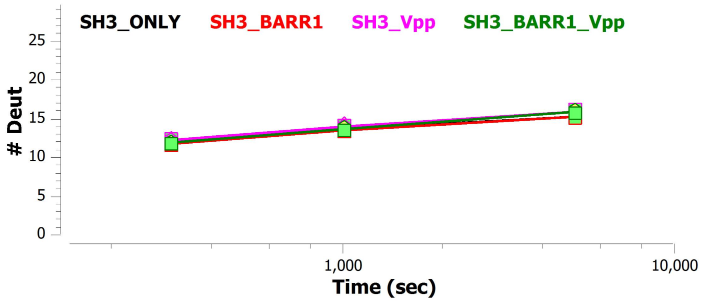

SH3 80-93: YVAPSDDYKDDDDK (#44)

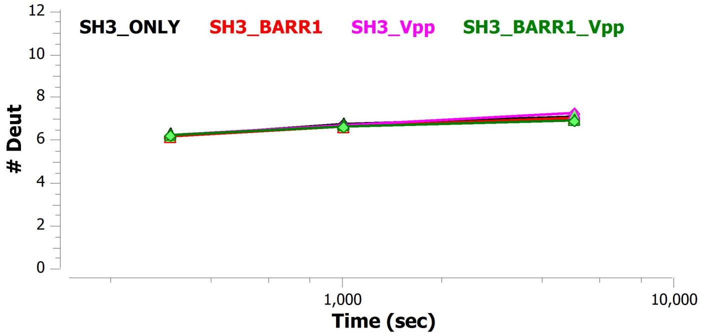

SH3 81-87: VAPSDDY (#45)

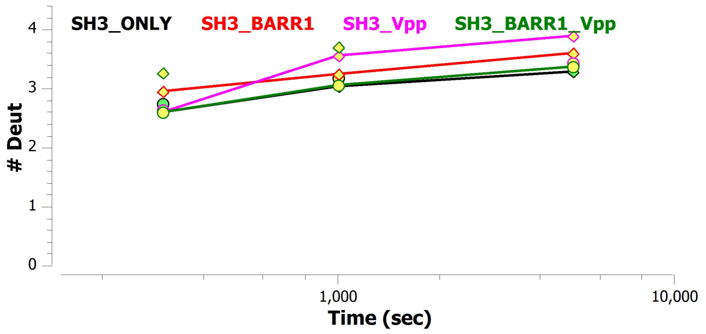

SH3 81-93: VAPSDDYKDDDDK (#46)

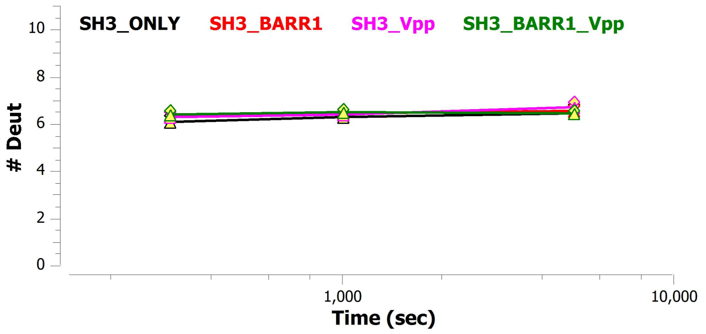

Supplement: Supplementary file 8 — Supplementary Data 6 [file 41467_2026_69884_MOESM8_ESM.pdf]
