# Supplementary material for: Mechanism of beta-arrestin 1 mediated Src activation via Src SH3 domain revealed by cryo-electron microscopy
Source: Nat Commun. 2026 Feb 20;17:2973. doi: 10.1038/s41467-026-69884-1 (PMC13035853; doi:10.1038/s41467-026-69884-1)
Supplement: Supplementary file 12 — Reporting Summary [file 41467_2026_69884_MOESM12_ESM.pdf]

Corresponding author(s): Lefkowitz R.J.

Last updated by author(s): Jan 26, 2026

Reporting Summary

Nature Portfolio wishes to improve the reproducibility of the work that we publish. This form provides structure for consistency and transparency in reporting. For further information on Nature Portfolio policies, see our Editorial Policies and the Editorial Policy Checklist.

Statistics

For all statistical analyses, confirm that the following items are present in the figure legend, table legend, main text, or Methods section.

n/a	Confirmed	
☐	☒	The exact sample size (*n*) for each experimental group/condition, given as a discrete number and unit of measurement
☐	☒	A statement on whether measurements were taken from distinct samples or whether the same sample was measured repeatedly
☐	☒	The statistical test(s) used AND whether they are one- or two-sided<br>*Only common tests should be described solely by name; describe more complex techniques in the Methods section.*
☒	☐	A description of all covariates tested
☐	☒	A description of any assumptions or corrections, such as tests of normality and adjustment for multiple comparisons
☐	☒	A full description of the statistical parameters including central tendency (e.g. means) or other basic estimates (e.g. regression coefficient) AND variation (e.g. standard deviation) or associated estimates of uncertainty (e.g. confidence intervals)
☐	☒	For null hypothesis testing, the test statistic (e.g. *F*, *t*, *r*) with confidence intervals, effect sizes, degrees of freedom and *P* value noted<br>*Give P values as exact values whenever suitable.*
☒	☐	For Bayesian analysis, information on the choice of priors and Markov chain Monte Carlo settings
☒	☐	For hierarchical and complex designs, identification of the appropriate level for tests and full reporting of outcomes
☒	☐	Estimates of effect sizes (e.g. Cohen's *d*, Pearson's *r*), indicating how they were calculated

Our web collection on statistics for biologists contains articles on many of the points above.

Software and code

Policy information about availability of computer code

Data collection	Latitude-S (Gatan) was used for automated cryo-EM data collection.
Data analysis	Cryo-EM data processing and 3D reconstruction were performed using CryoSPARC v4.0.1 and RELION v3.1. Initial particle picking was performed with Topaz v0.2.5a. The maps were postprocessed with spIsoNet and DeepEMhancer. UCSF Chimera v1.15, Coot v0.9.8.3 and ISOLDE in UCSF ChimeraX 1.6.1 were used for model building. Pymol 3.1.6.1 and UCSF ChimeraX 1.6.1 were used to make structural figures. Molecular dynamics flexible fitting was performed using Cryo fit and Namdinator. PHENIX v1.20.1-4487 and MolProbity v4.5.1 were used for model refinement and validation. The densitometry analysis of the Coomassie blue gels and Western blot images was performed by ImageJ v1.52a and ImageLab v6.1. The HDX data were analysed using Bruker Compass, Biotools software packages and HDExaminer-3. The ITC data were analysed using MicroCal Origin software package. The cross-linked peptides were identified using MaxQuant v.2.6.3.0. Statistical comparisons were performed using GraphPad Prism v9.

For manuscripts utilizing custom algorithms or software that are central to the research but not yet described in published literature, software must be made available to editors and reviewers. We strongly encourage code deposition in a community repository (e.g. GitHub). See the Nature Portfolio guidelines for submitting code & software for further information.

Data

Policy information about availability of data

All manuscripts must include a data availability statement. This statement should provide the following information, where applicable:

- Accession codes, unique identifiers, or web links for publicly available datasets
- A description of any restrictions on data availability
- For clinical datasets or third party data, please ensure that the statement adheres to our policy

The cryo-EM maps have been deposited in the EMDB under accession codes EMD-45977 (SH3–βarr1-CC complex), EMD-45982 (SH3–βarr1-N complex), and EMD-44881(Src–βarr1-CC complex). The atomic coordinates have been deposited in the Protein Data Bank under accession codes 9CX3 [https://doi.org/10.2210/pdb9CX3/pdb] (SH3–βarr1-CC complex); 9CX9 [https://doi.org/10.2210/pdb9CX9/pdb] (SH3–βarr1-N complex); 9BT8 [https://doi.org/10.2210/pdb9BT8/pdb] (Src–βarr1-CC complex). The HDX-MS data have been deposited to the ProteomeXchange Consortium (http://proteomecentral.proteomexchange.org) via the MassIVE repository (https://massive.ucsd.edu/) with the dataset identifier PXD073493 (MSV000100571). CXMS data have been deposited to the ProteomeXchange Consortium (http://proteomecentral.proteomexchange.org) via the PRIDE partner repository with the dataset identifier PXD073058. All other data generated or analyzed in this study are included in the article and its Supplementary Information. Source data underlying Figures 1c-d, 2d-e, 3b-c, 4d, 5d, 7a-c, 8b-c, 8f, and Supplementary Figures 2a, 5a, 5c-d, 7a are provided as a Source Data File.
The manuscript refers to the following previously published PDB accession codes:
4JQI [http://doi.org/10.2210/pdb4JQI/pdb] (βarr1–V2Rpp);
2PTK [http://doi.org/10.2210/pdb2PTK/pdb] (Src);
1G4M [http://doi.org/10.2210/pdb1G4M/pdb] (βarr1);
1FMK [http://doi.org/10.2210/pdb1FMK/pdb] (Src);
6TKO [http://doi.org/10.2210/pdb6TKO/pdb] (β1V2R–βarr1);
6U1N [http://doi.org/10.2210/pdb6U1N/pdb] (M2V2R–βarr1);
6UP7 [http://doi.org/10.2210/pdb6UP7/pdb] (NTSR1–βarr1);
4U5W [http://doi.org/10.2210/pdb4U5W/pdb] (Hck–Nef);
2KNB [http://doi.org/10.2210/pdb2KNB/pdb] (endophilin A1 SH3–parkin Ubl);
3A98 [http://doi.org/10.2210/pdb3A98/pdb] (DOCK2 SH3–ELMO 1);
1JT4 [http://doi.org/10.2210/pdb1JT4/pdb] (Sla1 SH3-ubiquitin);
1Y57 [http://doi.org/10.2210/pdb1Y57/pdb] (Src).

Research involving human participants, their data, or biological material

Policy information about studies with human participants or human data. See also policy information about sex, gender (identity/presentation), and sexual orientation and race, ethnicity and racism.

Reporting on sex and gender	N/A
Reporting on race, ethnicity, or other socially relevant groupings	N/A
Population characteristics	N/A
Recruitment	N/A
Ethics oversight	N/A

Note that full information on the approval of the study protocol must also be provided in the manuscript.

Field-specific reporting

Please select the one below that is the best fit for your research. If you are not sure, read the appropriate sections before making your selection.

☒ Life sciences ☐ Behavioural & social sciences ☐ Ecological, evolutionary & environmental sciences

For a reference copy of the document with all sections, see nature.com/documents/nr-reporting-summary-flat.pdf

Life sciences study design

All studies must disclose on these points even when the disclosure is negative.

Sample size	No sample size calculation was performed. Sample size was chosen according to the standards of the field (at least three independent biological replicates).
Data exclusions	No data were systematically excluded from analysis.
Replication	A minimum of three independent experiments were performed, and no data were excluded from analysis. All attempts at replication were successful.

Randomization | No randomization was performed because the study does not involve comparative treatment groups or allocation of samples to different experimental conditions. Therefore, randomization would not reduce bias or improve rigor.

Blinding | Researchers were not blinded in this study.

Reporting for specific materials, systems and methods

We require information from authors about some types of materials, experimental systems and methods used in many studies. Here, indicate whether each material, system or method listed is relevant to your study. If you are not sure if a list item applies to your research, read the appropriate section before selecting a response.

Materials & experimental systems

n/a	Involved in the study	
☐	☒	Antibodies
☐	☒	Eukaryotic cell lines
☒	☐	Palaeontology and archaeology
☒	☐	Animals and other organisms
☒	☐	Clinical data
☒	☐	Dual use research of concern
☒	☐	Plants

Methods

n/a	Involved in the study	
☒	☐	ChIP-seq
☒	☐	Flow cytometry
☒	☐	MRI-based neuroimaging

Antibodies

Antibodies used | Monoclonal ANTI-FLAG M2 peroxidase (HRP) antibody (1:2000) (A8592, Sigma-Aldrich, RRID: AB_439702) for β2V2 and SH3, polyclonal A1CT antibody generated in Lefkowitz lab (1:5000) for wild-type and mutant βarr1, anti-Src monoclonal antibody (1:1000) (MA5-15214, Thermo Fisher Scientific, RRID: AB_10980540) for total Src, anti-Src polyclonal Y418 for overexpressed Src (1:5000) (ab4816, Abcam, RRID: AB_304652), and Phospho-Src family polyclonal Y416 for endogenous Src (1:10000) (2101, Cell Signaling, RRID: AB_331697) were used. Secondary antibody included HRP-conjugated ECL Rabbit IgG (1:5000) (NA9340, Amersham, RRID:AB_772191) and HRP-conjugated ECL Mouse IgG (1:5000) (NA9310, Amersham, RRID:AB_772193).

Validation | The polyclonal A1CT antibody generated in Lefkowitz lab was reported and validated in Attramadal et al. 1992, Journal of Biological Chemistry 267(17882). All other primary antibodies were validated by the manufacturer for Western blot application.

Eukaryotic cell lines

Policy information about cell lines and Sex and Gender in Research

Cell line source(s) | Human embryonic kidney CRISPR-Cas9-based βarr1/βarr2 double knock-out cell line was characterized and reported in O'Hayre et al. 2017, Science Signaling 10(484).
Tetracycline-inducible Expi293F cells (Invitrogen, A39241).
Expi-293F cells (Invitrogen, A14527).

Authentication | No additional authentication of cell lines was performed.

Mycoplasma contamination | Cells were not tested for mycoplasma contamination.

Commonly misidentified lines (See ICLAC register) | No commonly misidentified cell lines were used.

Plants

Seed stocks | N/A

Novel plant genotypes | N/A

Authentication | N/A
